# The transcriptomic responses of Atlantic salmon (*Salmo salar*) to high temperature stress alone, and in combination with moderate hypoxia

**DOI:** 10.1186/s12864-021-07464-x

**Published:** 2021-04-12

**Authors:** Anne Beemelmanns, Fábio S. Zanuzzo, Xi Xue, Rebeccah M. Sandrelli, Matthew L. Rise, A. Kurt Gamperl

**Affiliations:** 1 Department of Ocean Sciences, Memorial University, St. John’s, NL A1C 5S7 Canada; 2 Current Address: Département de Biologie, Institut de Biologie Intégrative et des Systèmes (IBIS), Université Laval, Québec City, QC G1V 0A6 Canada

**Keywords:** Climate change, Increasing temperature, Hypoxia, Transcriptomics, Biomarker genes, Aquaculture

## Abstract

**Background:**

Increases in ocean temperatures and in the frequency and severity of hypoxic events are expected with climate change, and may become a challenge for cultured Atlantic salmon and negatively affect their growth, immunology and welfare. Thus, we examined how an incremental temperature increase alone (Warm & Normoxic-WN: 12 → 20 °C; 1 °C week^− 1^), and in combination with moderate hypoxia (Warm & Hypoxic-WH: ~ 70% air saturation), impacted the salmon’s hepatic transcriptome expr\ession compared to control fish (CT: 12 °C, normoxic) using 44 K microarrays and qPCR.

**Results:**

Overall, we identified 2894 differentially expressed probes (DEPs, FDR < 5%), that included 1111 shared DEPs, while 789 and 994 DEPs were specific to WN and WH fish, respectively. Pathway analysis indicated that the cellular mechanisms affected by the two experimental conditions were quite similar, with up-regulated genes functionally associated with the heat shock response, ER-stress, apoptosis and immune defence, while genes connected with general metabolic processes, proteolysis and oxidation-reduction were largely suppressed. The qPCR assessment of 41 microarray-identified genes validated that the heat shock response (*hsp90aa1, serpinh1*), apoptosis (*casp8, jund, jak2*) and immune responses (*apod, c1ql2, epx*) were up-regulated in WN and WH fish, while oxidative stress and hypoxia sensitive genes were down-regulated (*cirbp, cyp1a1, egln2, gstt1, hif1α, prdx6, rraga, ucp2*). However, the additional challenge of hypoxia resulted in more pronounced effects on heat shock and immune-related processes, including a stronger influence on the expression of 14 immune-related genes. Finally, robust correlations between the transcription of 19 genes and several phenotypic traits in WH fish suggest that changes in gene expression were related to impaired physiological and growth performance.

**Conclusion:**

Increasing temperature to 20 °C alone, and in combination with hypoxia, resulted in the differential expression of genes involved in similar pathways in Atlantic salmon. However, the expression responses of heat shock and immune-relevant genes in fish exposed to 20 °C and hypoxia were more affected, and strongly related to phenotypic characteristics (e.g., growth). This study provides valuable information on how these two environmental challenges affect the expression of stress-, metabolic- and immune-related genes and pathways, and identifies potential biomarker genes for improving our understanding of fish health and welfare.

**Supplementary Information:**

The online version contains supplementary material available at 10.1186/s12864-021-07464-x.

## Background

Temperature and oxygen are key environmental factors that influence the physiology, metabolism and survival of marine organisms, including fish [1–6]. Aquatic environments are characterized by short- (i.e., heat waves) and long-term (i.e., seasonal) fluctuations in water temperatures, which may become a further challenge for marine fish species with global warming [7–9]. For example, global ocean temperatures are projected to increase by an additional 1–3 °C by the end of the century [9], and this will be associated with more widespread and severe periods of hypoxia (low dissolved oxygen [DO]) in coastal regions [10, 11].

Thermal stress responses in fish have been widely investigated, and involve the expression of evolutionarily conserved genes [12]. A number of studies have demonstrated that acute and/or long-term exposure to high temperatures results in extensive changes in gene transcription in different salmonid tissues [13–20]. These molecular responses include alterations in the expression of genes related to the heat shock response, protein folding and repair, stress-induced cell death/apoptosis, signal transduction, oxidative stress, the inflammatory response and a diversity of metabolic processes [13–17, 20–23]. Similarly, hypoxia has profound effects on a broad range of biochemical, physiological and behavioural processes in fishes, and has deleterious impacts on growth and reproduction that eventually influence health, welfare and survival [2–4, 24]. Under hypoxic conditions, fish suppress energy-requiring processes like protein synthesis, aerobic metabolism and mitochondrial energy production [25–28]. On the contrary, hypoxia stimulates anaerobic ATP production, lysosomal lipid trafficking/degradation, the antioxidant system, the cellular heat shock response and immune-related pathways [25–27, 29–31]. In previous experiments fish have generally been exposed to an acute temperature increase or constant high temperatures [13–15, 17, 19, 22, 32], rather than the long-term incremental rise in temperature that is seen at aquaculture sites in coastal regions in temperate zones [33, 34]. Given the predicted increase in these two environmental stressors with global climate change [7, 9–11], it is of great importance that we assess their combined effects in experiments that simulate real-world conditions [35].

The Atlantic salmon is the most important commercially farmed salmonid species in the world. Juvenile and adult salmon reared in sea-cages in the North Atlantic are facing surface water temperatures up to 18–20 °C for extended periods in the summer [33, 34], while in Tasmania water temperatures inside the cages have already reached ~ 23 °C [36]. Yet, Atlantic salmon have an optimal growth performance at water temperatures between 10 and 14 °C [37, 38]. In addition, water oxygen levels within the cages fluctuate substantially due to temperature, fish density, feeding and low water exchange [39–41], and hypoxic events (~ 60–70% air saturation) often occur in late summer [33, 34, 36]. These suboptimal conditions may negatively affect the salmon’s physiological and growth performance [1, 42], and recently led to mass mortalities at cage-sites in Newfoundland, Canada [33]. Consequently, these conditions are raising concerns worldwide about the profitability of the industry and salmon welfare and survival [1, 36]. Nevertheless, we have limited knowledge about the capacity of Atlantic salmon to tolerate heat stress in combination with hypoxia, and whether these stressors interact synergistically or antagonistically, or impose additive effects [4, 35].

**Fig. 1:**
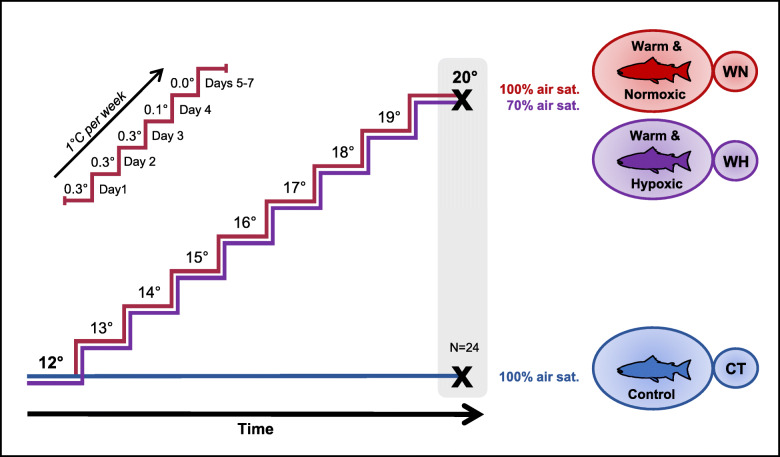
Schematic diagram of the experimental design. Post-smolt Atlantic salmon were either subjected to: *i*) a constant water temperature of 12 °C and normoxia (100% air saturation) (Control, CT); *ii*) a temperature increase from 12 to 20 °C and normoxia (100% air sat.) (Warm & Normoxic, WN); or *iii*) a temperature increase from 12 to 20 °C and moderate hypoxia (~ 70% air sat.) (Warm & Hypoxic, WH). The temperature was increased by 1 °C per week, following the temperature regimen shown in the upper left portion of the figure. Three days after the temperature increase to 20 °C liver samples were collected (*n* = 8 per treatment, *N* = 24 total) for transcriptomic screening (44 K Agilient Microarray) and qPCR (Fluidigm Biomark™) assessment/validation of transcript expression

In this study, we explored the hepatic transcriptional response of post-smolt salmon exposed to an incremental increase in temperature (12 → 20 °C at 1 °C week^− 1^) and normoxia (∼100% air saturation) (Warm & Normoxic-WN) or in combination with moderate hypoxia (~ 70% air sat.) (Warm & Hypoxic-WH), as compared to control conditions (12 °C, normoxia) (Control-CT) (Fig. 1). The WH condition simulates the environmental challenges that farmed Atlantic salmon can experience during the late summer/fall in sea-cages in the North Atlantic [33, 34, 41]. The liver was chosen to study due to its roles in a number of biological processes including the stress response, nutrient metabolism and immunity [24, 43], and because it has previously been shown to be an excellent organ for characterizing temperature and hypoxia stress responses in this species [14, 16]. An Agilent 44 K salmonid oligonucleotide microarray platform [44] was employed to initially assess hepatic transcriptome changes, and to elucidate the processes and mechanisms involved in the liver’s response once temperature reached 20 °C. Further, we performed real-time quantitative polymerase chain reaction (qPCR, Fluidigm Biomark™) on 41 target genes to: *i*) validate the microarray results; *ii*) examine the salmon’s molecular stress and immune responses to these environmental stressors; *iii*) correlate gene expression responses to physiological and growth parameters; and *iv*) identify genes that have potential as biomarkers for improving our understanding of Atlantic salmon health and welfare under sea-cage conditions predicted to accompany climate change, and for potential incorporation into broodstock selection programs.

## Results

### Significance analysis of microarrays (SAM)

**Fig. 2:**
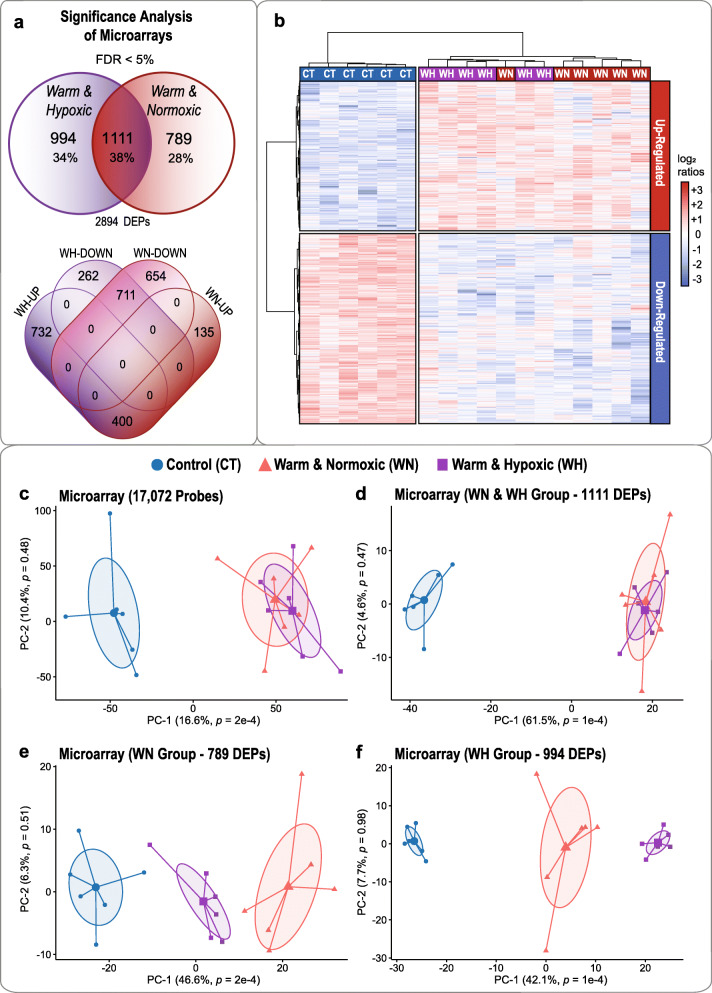
Hepatic transcriptome responses of Atlantic salmon exposed high temperature stress alone, or combined with hypoxia. **a** Results of Significance Analysis of Microarrays (SAM) with a False Discovery Rate (FDR) of < 5% (see Additional file 1). The top Venn diagram illustrates the total number of differentially expressed probes (DEPs) in salmon exposed to Warm & Normoxic (WN: 20 °C, 100% air saturation) or Warm & Hypoxic (WH: 20 °C, ~ 70% air sat.) conditions as compared to the Control group (CT: 12 °C, 100% air sat.) (*n* = 6 per treatment, *N* = 18 total). The bottom Venn diagram shows the corresponding number of DEPs that were up- or down-regulated. The overlapping areas represent shared DEPs between the WN and WH treatment groups. **b** Hierarchically clustered heat map based on 2894 DEPs (FDR < 5%) using Ward’s minimum variance method. Heatmap and dendrograms illustrate the clustering structure of 2894 SAM-identified DEPs for fish subjected to control conditions (CT), high temperature and normoxia (WN), or high temperature and hypoxia (WH). The integrated colour code shows up-regulated DEPs in red and down-regulated DEPs in blue, and represents normalized log_2_ ratios. **c** Principal Component Analysis (PCA) based on all detected 17,072 microarray probes of fish exposed to CT, WN and WH conditions; **d** PCA based on a common set of 1111 DEPs shared between the WN and WH treatment groups; **e** PCA based on 789 DEPs in the WN treatment group; **f** PCA based on 994 DEPs in the WH treatment group. Each PCA plot includes the three different treatment groups (CT, WN and WH), and is based on normalized log_2_ ratios. Ellipses denote the dispersion of variance with 95% confidence intervals around the the center of the distribution for each treatment group. The variance explained by the main Principal Components (PC-1 and PC-2) are indicated in percentage values next to the axes

Based on 17,072 detected microarray probes, the pairwise comparisons computed within SAM recognized 1900 differentially expressed probes (DEPs) for WN challenged fish and 2105 DEPs for WH treated fish as compared to CT fish (Fig. 2a). The complete annotation of SAM-identified DEPs for the WN and WH treatment groups is listed in Additional file 1. When comparing the ‘WH vs CT’ and ‘WN vs CT’ DEP lists, we found that 1111 DEPs (38%) were overlapping (i.e., were in common), that 789 DEPs (28%) were WN-specific, and that 994 DEPs (34%) were WH-specific (Fig. 2a). In summary, we identified a unique set of 2894 DEPs when considering WN and WH conditions together, out of which 1267 DEPs were up-regulated (WH = 732, WN = 135; shared for WH and WN = 400) and 1627 DEPs were down-regulated (WH = 262, WN = 654; shared for WH and WN = 711) (Fig. 2a; Additional file 1). A hierarchically clustered heat map of the 2894 DEPs displayed a robust cluster containing all CT samples that grouped separately from the cluster containing both treatment groups, with distinctive opposing patterns of up- and down-regulation (Fig. 2b). According to Ward’s cluster algorithm, the profiles of WN and WH fish created a uniform cluster with a similar magnitude of expression (Fig. 2b).

The Principal Component Analysis (PCA) based on 17,072 detected microarray features displayed a similar global transcription profile for fish exposed to WN or WH conditions in comparison to CT fish that was separated along the first Principal Component (PC-1: *p* = 2e-4, explaining 16.6% of the variance; CT vs WN *p* = 2e-4, CT vs WH *p* = 0.001, WN vs WH *p* = 0.489; Fig. 2c and Table 1). Similarly, we found a comparable global transcript expression response for the shared 1111 DEPs, as shown by equal cluster distribution for the WN and WH treatment groups as compared to the CT group (PC-1: *p* = 1e-4, explaining 61.5% of the variance; CT vs WN *p* < 0.0001, CT vs WH *p* = 2e-4, WN vs WH *p* = 0.841; Fig. 2d and Table 1). However, the 789 WN-specific DEPs displayed stronger differential expression in WN fish as compared to WH fish, as indicated by a clear cluster separation between all three treatment groups (PC-1: *p* = 2e-4, explaining 46.6% of the variance; CT vs WN *p* = 6e-4, CT vs WH *p* = 2e-4, WN vs WH *p* = 0.012; Fig. 2e and Table 1); and the 994 WH-specific DEPs displayed stronger differential expression in WH fish, as evidenced by an extreme shift away from the CT and WN groups (PC-1: *p* = 1e-4, explaining 42.1% of the variance; CT vs WN *p* = 0.002, CT vs WH *p* < 0.0001, WN vs WH *p* = 0.007; Fig. 2f and Table 1).

**Table 1:** Temperature and hypoxia treatment effects on the extracted scores of the first two principal components

Linear Mixed-Effect Models For Each Principal Component (PC-1 and PC-2)^**a**^										
Microarray^**b**^	PC	Var %	Factors	Num DF	Den DF	F-value	***p***-value	CT vs WN	CT vs WH	WN vs WH
**All Probes (17,072)**	**PC-1**	**16.6**	Intercept	1	12	20.6	**7e-4**			
			Treatment	2	3	384.6	**2e-4**	**2e-4**	**0.001**	0.489
	**PC-2**	**10.4**	Intercept	1	12	0.4	0.531			
			Treatment	2	3	1.0	0.477			
**DEPs WN & WH (1111)**	**PC-1**	**61.5**	Intercept	1	12	19.1	**9e-4**			
			Treatment	2	3	687.6	**1e-4**	**< 0.0001**	**2e-4**	0.841
	**PC-2**	**4.6**	Intercept	1	12	0.3	0.623			
			Treatment	2	3	1.0	0.470			
**DEPs WN (789)**	**PC-1**	**46.6**	Intercept	1	12	819.3	**< 0.0001**			
			Treatment	2	3	506.0	**2e-4**	**6e-4**	**2e-4**	**0.012**
	**PC-2**	**6.3**	Intercept	1	12	1.8	0.209			
			Treatment	2	3	0.9	0.506			
**DEPs WH (994)**	**PC-1**	**42.1**	Intercept	1	12	252.3	**< 0.0001**			
			Treatment	2	3	875.0	**1e-4**	**0.002**	**< 0.0001**	**0.007**
	**PC-2**	**7.7**	Intercept	1	12	0.6	0.441			
			Treatment	2	3	0.0	0.982			
**qPCR**^**c**^										
**All Target Genes (41)**	**PC-1**	**39.4**	Intercept	1	18	147.5	**< 0.0001**			
			Treatment	2	3	318.4	**3e-4**	**3e-4**	**6e-4**	0.747
	**PC-2**	**18.2**	Intercept	1	18	46.9	**< 0.0001**			
			Treatment	2	3	1.2	0.416			
**Stress-Related Genes (24)**	**PC-1**	**45.9**	Intercept	1	18	713.9	**< 0.0001**			
			Treatment	2	3	488.3	**2e-4**	**1e-4**	**2e-4**	0.537
	**PC-2**	**19.6**	Intercept	1	18	16.9	**7e-4**			
			Treatment	2	3	1.2	0.423			
**Immune-Related Genes (14)**	**PC-1**	**30.7**	Intercept	1	18	1.5	0.237			
			Treatment	2	3	32.8	**0.009**	**0.011**	**0.012**	0.405
	**PC-2**	**23.6**	Intercept	1	18	0.0	0.870			
			Treatment	2	3	1.2	0.417			

^a^ Linear mixed-effect models (LMEs) were performed for the first two principal components (PC-1 and PC-2) individually to assess the effect of the fixed factor ‘treatment’ and included the random term ‘tank’. The variance explained by each PC is indicated in percentage (%) values. For the pairwise comparisons between the Control (CT), Warm & Normoxic (WN) and Warm & Hypoxic (WH) treatment groups, *lsmeans* post-hoc tests with Tukey’s multiple comparisons adjustment of *p-*values were applied. Significant *p*-values are marked in bold letters (*p* < 0.05)

^b^ Statistical approach based on normalized log_2_ ratios of 17,072 detected microarray probes, 1111 WN & WH-specific differentially expressed probes (DEPs), 789 WN-specific DEPs and 994 WH-specific DEPs

^c^ Statistical approach based on the relative expression values (RQ) of 41 target genes, 24 stress-related genes and 14 immune-related genes measured with qPCR (Fluidigm Biomark™) (see Table 2)

### GO/pathway term network analysis

**Fig. 3:**
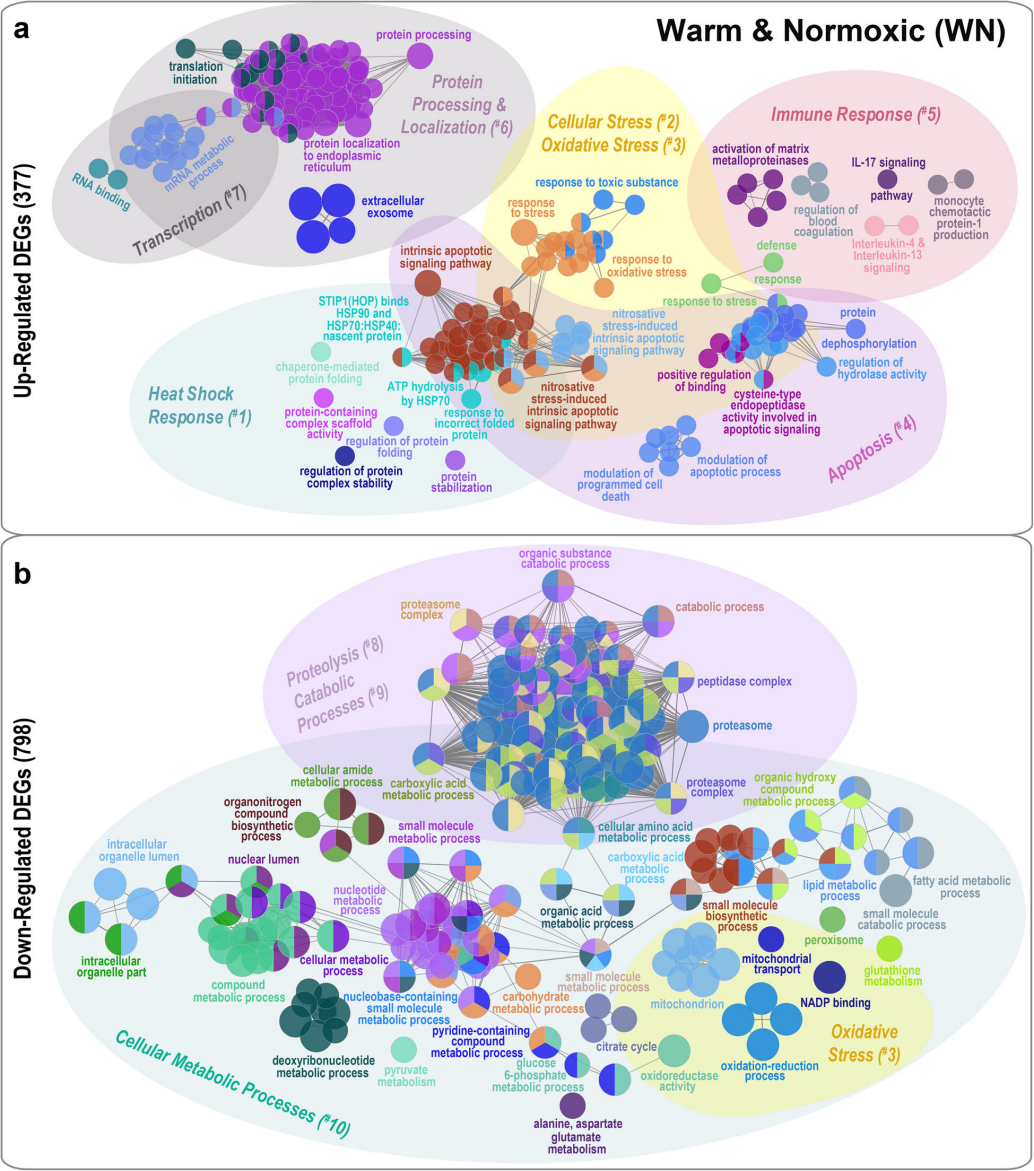
Functionally grouped gene network analysis for Atlantic salmon exposed to Warm & Normoxic conditions. Network based on **a** 377 up-regulated and **b** 798 down-regulated differentially expressed genes (DEGs) in salmon exposed to Warm & Normoxic (WN: 20 °C, 100% air saturation) conditions as compared to the Control group (CT: 12 °C, 100% air sat.) (n = 6 per treatment, *N* = 12 total). GO-terms and pathways were obtained through a functional enrichment analysis using the ClueGO plugin in Cytoscape (v3.5.1) [45]. Each node represents a significantly enriched Gene Ontology (GO), KEGG or Reactome pathway (hypergeometric test *p <* 0.05 with Benjamini-Hochberg correction). The node colour regime visualizes functional groups and processes that share similar genes. The most significant enriched terms of each functional group are illustrated as a summary label. The node circle diameter corresponds to the significance of the enriched pathway (i.e., larger circles correspond to higher significance). The gating and numbering represent the classification of ten functional themes: Heat Shock Response (^#^1), Cellular Stress (^#^2), Oxidative Stress (^#^3), Apoptosis (^#^4), Immune Response (^#^5), Protein Processing & Localization (^#^6), Transcription (^#^7), Proteolysis (^#^8), Catabolic Processes (^#^9) and Cellular Metabolic Processes (^#^10). GO/pathway term annotations were obtained using the GO database for biological process, cellular component, molecular function and immune processes, and the KEGG and Reactome pathway databases. Abbreviations of GO/pathway terms were used for simplification, and the complete list of terms is represented in Additional files 3 and 5

**Fig. 4:**
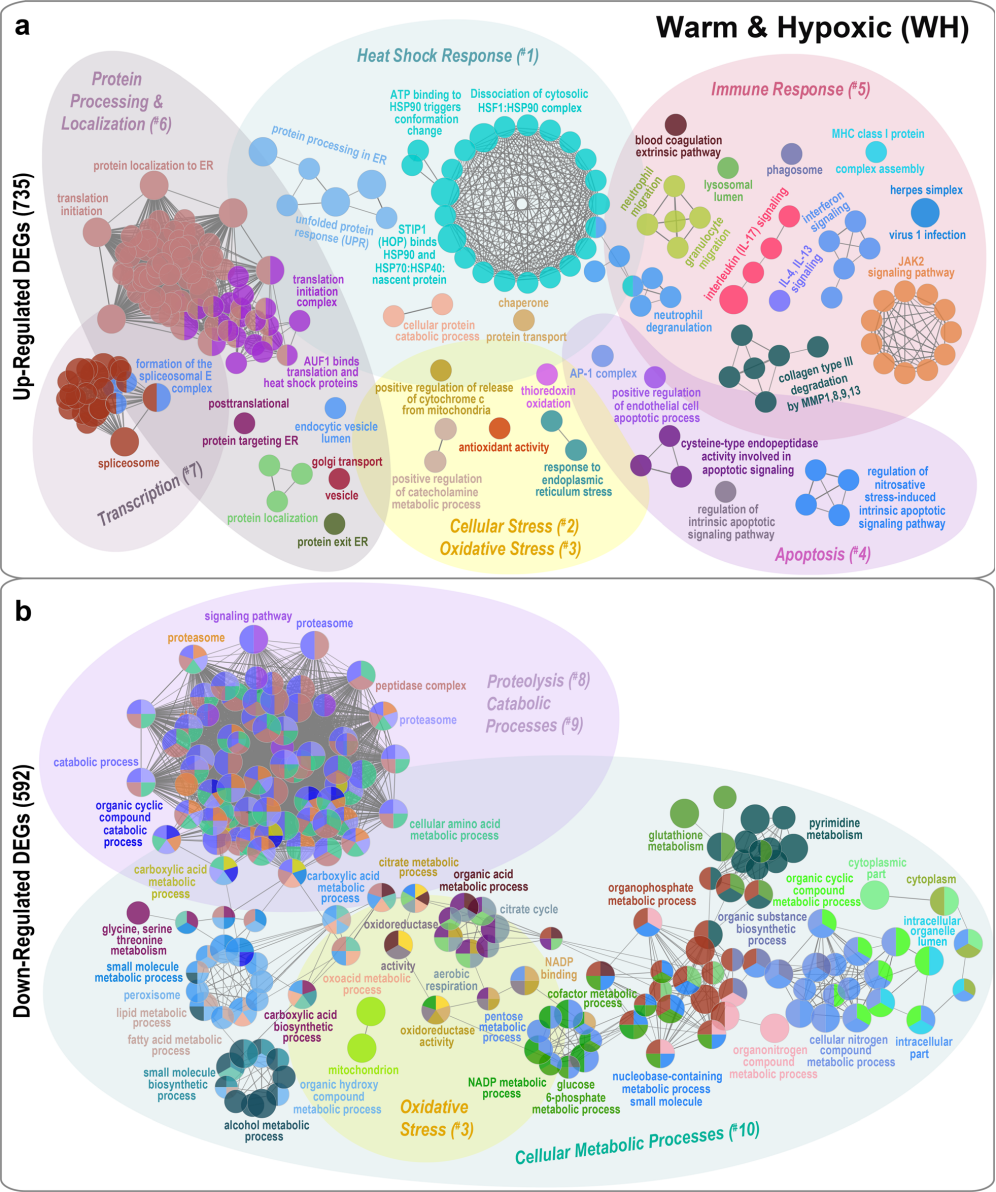
Functionally grouped gene network analysis for Atlantic salmon subjected to Warm & Hypoxic conditions. Network based on **a** 735 up-regulated and **b** 592 down-regulated differentially expressed genes (DEGs) in salmon exposed to Warm & Hypoxic (WH: 20 °C, ~ 70% air saturation) conditions as compared to the Control group (CT: 12 °C, 100% air sat.) (n = 6 per treatment, N = 12 total). GO-terms and pathways were obtained through a functional enrichment analysis using the ClueGO plugin in Cytoscape (v3.5.1) [45]. Each node represents a significantly enriched Gene Ontology (GO), KEGG or Reactome pathway (hypergeometric test *p <* 0.05 with Benjamini-Hochberg correction). The node colour regime visualizes functional groups and processes that share similar genes. The most significant enriched terms of each functional group are illustrated as a summary label. The node circle diameter corresponds to the significance of the enriched pathway (i.e., larger circles correspond to higher significance). The gating and numbering represent the classification of ten functional themes: Heat Shock Response (^#^1), Cellular Stress (^#^2), Oxidative Stress (^#^3), Apoptosis (^#^4), Immune Response (^#^5), Protein Processing & Localization (^#^6), Transcription (^#^7), Proteolysis (^#^8), Catabolic Processes (^#^9) and Cellular Metabolic Processes (^#^10). GO/pathway term annotations were obtained using the GO database for biological process, cellular component, molecular function and immune processes, and the KEGG and Reactome pathway databases. Abbreviations of GO/pathway terms were used for simplification, and the complete list of terms is represented in Additional files 4 and 6

Salmon exposed to either the WN or WH treatments had similar patterns for enriched ‘Gene Ontology’ (GO) terms, ‘Kyoto Encyclopedia of Genes and Genomes’ (KEGG) and ‘Reactome’ pathways (hereinafter referred to as ‘GO/pathway terms') for up- and down-regulated differentially expressed genes (DEGs) (Figs. 3 and 4; Additional files 2-6). For both treatment groups, the up-regulated DEGs were functionally associated with significantly enriched GO/pathway terms (*p* = 1e-2 to *p* = 1e-4) connected to Heat Shock Response (^#^1), Cellular Stress (^#^2), Oxidative Stress (^#^3), Apoptosis (^#^4), Immune Response (^#^5), Protein Processing & Localization (^#^6) and Transcription (^#^7) (Figs. 3a and 4a; Additional files 2a-b, 3 and 4). On the contrary, the down-regulated DEGs for WN and WH fish were associated, and with a higher significance, to enriched GO/pathway terms (*p* = 1e-5 to *p* = 1e-27) related to Oxidative Stress (^#^3), Proteolysis (^#^8), Catabolic Processes (^#^9) and Cellular Metabolic Processes (^#^10), and formed complex and interconnected functional clusters (Figs. 3b and 4b; Additional files 2c-d, 5 and 6). Below we list the shared and dissimilar most significantly enriched GO/pathway terms (with identifiers) for WN and WH fish as visualized in the functional ordered networks (Figs. 3 and 4). However, some terms were abbreviated for simplification purposes, and the complete lists of all terms are given in Additional files 3-6.

### GO/pathway term networks associated with up-regulated DEGs

#### Heat shock response (^#^1)

In the functionally grouped network analyses, WN and WH fish had up-regulated DEGs associated with enriched GO/pathway terms related to Heat Shock Response (^#^1) (Figs. 3a and 4a). WN fish had one large cluster (~ 8 terms) with the following enriched GO/pathway terms: ‘STIP1(HOP) binds HSP90 and HSP70:HSP40:nascent protein’ (R-HSA:3371503), ‘ATP hydrolysis by HSP70’ (R-HSA:3371422); and ‘response to topologically incorrect protein’ (GO:0035966) (Fig. 3a; Additional file 3). On the contrary, WH fish had two large and highly interconnected clusters (~ 30 terms) associated with the chaperone-mediated heat shock response that included the following enriched Reactome/KEGG pathways: ‘ATP binding to HSP90 triggers conformation change’ (R-HSA:5618107), ‘dissociation of cytosolic HSF1:HSP90 complex’ (R-HSA:5324632), ‘STIP1(HOP) binds HSP90 and HSP70:HSP40:nascent protein’ (R-HSA:3371503), ‘protein processing in endoplasmic reticulum’ (KEGG:04141), and ‘unfolded protein response (UPR)’ (R-HSA:381119) (Fig. 4a; Additional file 4). Also, similar GO-terms were significantly enriched in WN and WH fish related to ‘chaperone-mediated autophagy processes’ (WN: GO:0061684; WH: GO:0061741), ‘chaperone and protein folding responses’ (WN: GO:0061077; WH: GO:0030968), and ‘regulation of tau-protein kinase activity’ (WN + WH: GO:1902947, GO:1902949) (Additional files 2a-b).

#### Cellular stress (^#^2) and oxidative stress (^#^3)

Fish exposed to WN conditions had larger clusters of up-regulated DEGs associated with Cellular Stress (^#^2) and Oxidative Stress (^#^3), and contained the following significantly enriched GO-terms: ‘response to stress’ (GO:0006950), ‘response to oxidative stress’ (GO:0006979), ‘response to toxic substance’ (GO:0009636) and ‘regulation of response to stress’ (GO:0080134) (Fig. 3a; Additional file 3). Whereas, for fish subjected to WH conditions, we identified the following unique enriched oxidative stress-related GO-terms: ‘antioxidant activity’ (GO:0016209), ‘positive regulation of release of cytochrome c from mitochondria’ (GO:0090200), ‘positive regulation of catecholamine metabolic process’ (GO:0045915), ‘positive regulation of transcription in response to endoplasmic reticulum stress’ (GO:199044) (Fig. 4a; Additional file 4).

#### Apoptosis (^#^4)

For WN fish, we found up-regulated DEGs associated with six large clusters containing ~ 92 highly interconnected enriched GO/pathway terms related to Apoptosis (^#^4), such as ‘intrinsic apoptotic signaling pathway in response to nitrosative stress’ (GO:1990442), ‘regulation of nitrosative stress-induced intrinsic apoptotic signaling’ (GO:1905258), ‘modulation of programmed cell death/apoptotic process in other organism’ (GO:0044531, GO:0044532), and ‘regulation of hydrolase activity’ (GO:0051336) (Fig. 3a; Additional file 3). In contrast, WH fish had five smaller clusters containing ~ 10 enriched GO/pathway terms associated with apoptotic processes like ‘regulation of intrinsic apoptotic signaling pathway’ (GO:2001242), ‘positive regulation of endothelial cell apoptotic process’ (GO:2000353), and ‘transcription factor AP-1 complex’ (GO:0035976) (Fig. 4a; Additional file 4). Also, similar GO-terms related to ‘cysteine-type endopeptidase activity involved in apoptotic signaling pathway’ (GO:2001267, GO:0097199) and ‘regulation of nitrosative stress-induced intrinsic apoptotic signaling pathway’ (GO:1905258, GO:1990442, GO:1905259) were enriched in WN and WH fish (Figs. 3a and 4a; Additional files 2a-b, 3 and 4).

#### Immune response (^#^5)

Pronounced differences between the WH and WN groups were found for pathways related to immune response (^#^5) (Figs. 3a and 4a; Additional files 3 and 4). Up-regulated DEGs in the WN group were related to enriched immune-related GO/pathway terms (five groups, ~ 13 terms) such as ‘IL-17 signaling pathway’ (KEGG:04657), ‘Interleukin-4 and Interleukin-13 signaling’ (R-HSA:6785807), ‘monocyte chemotactic protein-1 production’ (GO:0071605), ‘activation of matrix metalloproteinases’ (R-HSA:1592389), and ‘regulation of blood coagulation’ (GO:0030193) (Fig. 3a; Additional file 3). In comparison, the up-regulated DEGs in the WH group formed more complex immune-related functional clusters (12 groups, ~ 43 terms) that were associated with ‘neutrophil/granulocyte migration and granulation’ (GO:0097530, GO:0071621, GO:1990266, GO:0030593), ‘phagosome’ (KEGG:04145), ‘JAK2 growth hormone receptor signaling’ (R-HSA:6784189) and with interleukin signaling pathways such as ‘IL-17 signaling pathway’ (KEGG:04657) and ‘IL-4 and IL-13 signaling’ (R-HSA:6785807) (Fig. 4a; Additional files 2b and 4). GO/pathway terms connected to anti-viral responses were also activated such as ‘interferon signaling’ (R-HSA:913531), ‘herpes simplex virus 1 infection’ (KEGG:05168) and ‘MHC class I protein complex assembly’ (GO:0002397). Further, one cluster connected to extracellular matrix processing such as ‘collagen type III degradation by MMP1,8,9,13’ (R-HSA:1474213) and also ‘blood coagulation extrinsic pathways’ (GO:0007598) was enriched in the WH group (Fig. 4a; Additional files 2b and 4).

#### Protein processing & localization (^#^6) and transcription (^#^7)

Both experimental groups had complex functional network clusters linked to Protein Processing & Localization (^#^6) and Transcription (^#^7) (Figs. 3a and 4a) including ‘translation initiation’ (GO:0006413, R-HSA:72613), ‘protein localization to endoplasmic reticulum’ (WN: GO:0070972, WH: GO:0072599), ‘mRNA metabolic process’ (WN: GO:0016071, WH: GO:0000184) and ‘spliceosome’ or ‘RNA metabolic processes’ (WN: GO:0000184, WH: GO:0043484, KEGG:03040) (Figs. 3a and 4a; Additional files 2a-b, 3 and 4).

### GO/pathway term networks associated with down-regulated DEGs

#### Proteolysis (^#^8), catabolic processes (^#^9) and cellular metabolic processes (^#^10)

The most highly enriched GO/pathway terms were related to ‘small molecule metabolic process’ (GO:0044281,GO:0044283; WN: *p* = 1e-27; WH: *p* = 3e-10), ‘carboxylic acid metabolic process’ (GO:0019752; WN *p* = 1e-21, WH *p* = 1e-15), ‘organic acid metabolic process’ (GO:0006082; WN *p* = 2e-20, WH *p* = 3e-15) and ‘proteasome’ (KEGG:03050; WN *p* = 9e-20, WH *p* = 2e-13) (Figs. 3b and 4b; Additional files 5 and 6). For both treatment groups, we found large clusters with functional GO/pathway terms linked to Proteolysis (^#^8) and Catabolic Processes (^#^9) such as ‘proteasome’ (KEGG:03050, GO:0000502) and ‘peptidase complex’ (GO:1905368) (Figs. 3b and 4b; Additional files 5 and 6). The majority of enriched metabolic pathways can broadly be categorized as Cellular Metabolic Processes (^#^10) [e.g., ‘cellular amino acid metabolic process’ (GO:0006520), ‘carboxylic acid metabolic process’ (GO:0019752), ‘lipid metabolic process’ (GO:0006629), ‘fatty acid metabolic process’ (GO:0006631), ‘nucleobase-containing small molecule metabolic process’ (GO:0055086), ‘glucose 6-phosphate metabolic process’ (GO:0051156), and ‘citrate cycle’ (TCA cycle) (KEGG:00020, GO:0006101) (Figs. 3b and 4b; Additional files 2c-d, 5 and 6). Interestingly, the down-regulated DEGs of WH fish formed four interconnected clusters associated with Oxidative Stress (^#^3), which included ‘oxidoreductase activity’ (GO:0016491), ‘oxidoreductase activity, acting on CH-OH group of donors’ (GO:0016614) and ‘mitochondrion’ (GO:0005739) (Fig. 4b; Additional file 6), while WN fish had two smaller clusters connected to ‘oxidation-reduction processes’ (GO:0055114, GO:0016614) and ‘mitochondrion’ (GO:0005739) (Fig. 3b; Additional file 5).

### qPCR validation of microarray approach

**Fig. 5:**
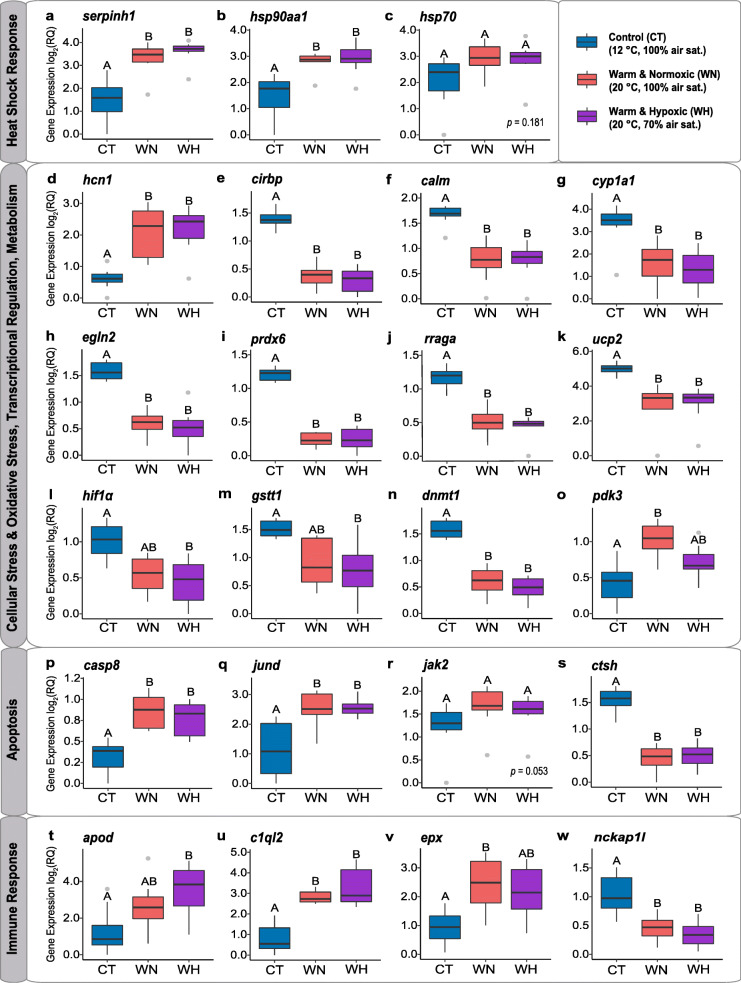
Hepatic transcript expression changes (qPCR-Fluidigm Biomark) for 23 target genes. Relative expression values [i.e., relative quantity (RQ)] per individual gene are plotted for Warm & Normoxic (WN: 20 °C, 100% air saturation) and Warm & Hypoxic (WH: 20 °C, ~ 70% air sat.) fish as compared to fish of the Control group (CT: 12 °C, 100% air sat.) (n = 8 per treatment, N = 24 total). The horizontal line within the box plots indicates the median value, the top and bottom limits of the bar indicate the 25th and 75th quartiles, the vertical lines indicate the maximum and minimum values, and the gray dots indicate outliers. Significant differences between groups are indicated by different capital letters (*lsmeans* post-hoc test, *p* < 0.05) and non-significant differences are shown by a *p*-value (*p* > 0.05) (see Table 3). The plots are sorted according to six functional themes. Heat Shock Response: **a** *serpinh1,* **b** *hsp90aa1,* **c** *hsp70*; Cellular and Oxidative Stress Response: **d** *hcn1,* **e** *cirbp,* **f** *calm,* **g** *cyp1a1,* **h** *egln2,* **i** *prdx6,* **j** *rraga,* **k** *ucp2,* **l** *hif1α* and **m** *gstt1*; Transcription: **n** *dnmt1*; Metabolism: **o** *pdk3*; Apoptosis: **p** *casp8,* **q** *jund,* **r** *jak2* and **s** *ctsh*; and Immune Response: **t** *apod,* **u** *c1ql2,* **v** *epx* and **w** *nckap1l*

We chose a subset of 41 microarray-identified genes of interest (GOIs) to confirm the microarray results using a gene-targeted qPCR approach (Table 2). A significant positive correlation (R = 0.87; *p <* 1.7e-13) between the mean log_2_ fold change (FC) values of the 41 GOIs measured via the 44 K microarray and qPCR methods indicates that the microarray results were validated with great confidence (Additional file 7). In a direct comparison between mean FC values of the microarray and qPCR approaches, 34 genes (83%) showed similar transcription patterns and FC values (Additional file 8). Minor differences were found for the genes *camp-a, cat* and *cldn3,* while *igfbp2b1, irf2, nckap1l* and *tapbp* had a difference in the direction of expression change (Additional file 8). Out of the 41 GOIs, we found 23 with a significant ‘treatment’ effect in the linear mixed-effect model (LME), while the other 18 genes were differentially expressed in the SAM analysis but not validated using qPCR (Table 3). This discrepancy may have been caused by: *i*) using two different technologies; *ii*) a different sample size; *iii*) two different statistical approaches (SAM vs LME); or *iv*) paralog cross-hybridization on the 44 K array. The relative expression values [i.e., relative quantity (RQ)] for a selection of GOIs are shown in Fig. 5.

**Table 2:** Functional annotation and fold change (FC) transcript expression for 41 qPCR genes

Gene Symbol^**a**^	Gene Name^**a**^	Functional Theme^**b**^	Key Function (UniProt)^**c**^	FC WN^d^	FC WH^e^
**Genes Related to Heat Shock Response**					
***hsp70***	**Heat shock protein HSP 70**	Heat Shock Response (^#^1)	**MF**: Chaperone, Receptor; **BP**: Stress response	2.0 ± 0.37	2.0 ± 0.38
***hsp90aa1***	**Heat shock protein HSP 90-alpha**	Heat Shock Response (^#^1)	**MF**: Chaperone; **BP**: Stress response	3.1 ± 0.31	3.7 ± 0.67
***hsp90ab1***	**Heat shock protein HSP 90-beta**	Heat Shock Response (^#^1)	**MF**: Chaperone; **BP**: Stress response	1.3 ± 0.19	1.4 ± 0.22
***hspd1***	**60 kDa heat shock protein**	Heat Shock Response (^#^1)	**MF**: Chaperone, Isomerase; **BP**: Stress response	0.3 ± 0.02	0.4 ± 0.04
***serpinh1 (hsp47)***	**Serpin H1**	Heat Shock Response (^#^1)	**MF**: Chaperone; **BP**: Stress response, Collagen biosynthesis	5.5 ± 0.89	6.3 ± 0.78
**Genes Related to Stress Response**					
***calm (cam)***	**Calmodulin**	Cellular Stress (^#^2), Oxidative Stress (^#^3)	**MF**: Calcium ion binding; **BP**: Calcium-mediated signaling	0.4 ± 0.06	0.4 ± 0.04
*** cat***	**Catalase**	Cellular Stress (^#^2), Oxidative Stress (^#^3) Apoptosis (^#^4)	**MF**: Oxidoreductase, Peroxidase; **BP**: Hydrogen peroxide	1 ± 0.14	1 ± 0.11
*** cirbp***	**Cold-inducible RNA-binding protein**	Heat Shock Response (^#^1) Cellular Stress (^#^2), Oxidative Stress (^#^3)	**MF**: Activator, Repressor, RNA-binding; **BP**: Stress response	0.4 ± 0.03	0.3 ± 0.03
*** cldn3***	**Claudin 3**	Cellular Stress (^#^2),	**MF**: Transmembrane signaling; **BP**: Tight junction, Protein binding	0.8 ± 0.07	1 ± 0.1
*** cul3***	**Cullin 3**	Cellular Stress (^#^2), Apoptosis (^#^4)	**MF**: Protein ubiquitination; **BP**: ER-Golgi transport, Transport	1.2 ± 0.08	1 ± 0.06
***cyp1a1***	**Cytochrome P450 1A1**	Cellular Stress (^#^2), Oxidative Stress (^#^3)	**MF**: Oxidoreductase; **BP**: Oxidation-reduction process	0.2 ± 0.05	0.2 ± 0.04
***egln2 (phd1)***	**Egl nine homolog 2**	Cellular Stress (^#^2), Oxidative Stress (^#^3), Apoptosis (^#^4)	**MF**: Oxidoreductase, Oxygen sensor activity; **BP**: Cell redox homeostasis, Response to hypoxia	0.4 ± 0.03	0.4 ± 0.05
***gstt1***	**Glutathione S-transferase theta-1**	Cellular Stress (^#^2), Oxidative Stress (^#^3)	**MF**: Transferase; **BP**: Glutathione metabolic process	0.6 ± 0.09	0.5 ± 0.09
*** hcn1***	**Hyperpolarization-activated cyclic nucleotide-gated channel 1**	Cellular Stress (^#^2), Oxidative Stress (^#^3)	**MF**: Ion channel; **BP**: Ion transport, Transport	5.4 ± 1.27	5.7 ± 1.06
*** hif1α***	**Hypoxia-inducible factor 1 alpha**	Cellular Stress (^#^2), Oxidative Stress (^#^3), Immune (^#^5)	**MF**: DNA-binding; Oxygen sensor activity; **BP**: Transcription regulation, Response to hypoxia	0.60 ± 0.06	0.57 ± 0.06
***igfbp2b1***	**Insulin-like growth factor-binding protein 2 - paralog b1**	Oxidative Stress (^#^3)	**MF**: Growth factor binding; **BP**: Growth regulation	0.6 ± 0.14	0.6 ± 0.15
*** jak2***	**Tyrosine-protein kinase JAK2-like**	Cellular Stress (^#^2), Oxidative Stress (^#^3), Apoptosis (#4), Immune (^#^5)	**MF**: Chromatin regulator, Tyrosine-protein kinase; **BP**: Apoptosis, Immunity	1.7 ± 0.22	1.5 ± 0.16
*** jund***	**Transcription factor Jun-D-like**	Cellular Stress (^#^2), Apoptosis (^#^4), Immune (^#^5)	**MF**: Activator, DNA-binding; **BP**: Transcription, Transcription regulation	3.2 ± 0.53	3 ± 0.35
***ndufa1***	**NADH dehydrogenase 1 alpha subcomplex subunit 1**	Cellular Stress (^#^2), Oxidative Stress (^#^3)	**MF**: NADH dehydrogenase; **BP**: Electron transport, Respiratory chain	1 ± 0.05	1 ± 0.02
***ndufa4***	**Cytochrome c oxidase subunit NDUFA4**	Cellular Stress (^#^2), Oxidative Stress (^#^3)	**MF**: NADH dehydrogenase; **BP**: Electron transport, Respiratory chain	2.2 ± 0.43	1.3 ± 0.45
*** prdx6***	**Peroxiredoxin 6**	Cellular Stress (^#^2), Oxidative Stress (^#^3)	**MF**: Antioxidant, Peroxidase; **BP**: Cellular response to oxidative stress	0.4 ± 0.01	0.4 ± 0.02
*** rraga***	**Ras-related GTP-binding protein A**	Cellular Stress (^#^2), Oxidative Stress (^#^3), Apoptosis (^#^4)	**MF**: GTP-binding, Hydrolase; **BP**: Apoptosis	0.5 ± 0.04	0.5 ± 0.03
*** txn***	**Thioredoxin**	Cellular Stress (^#^2), Oxidative Stress (^#^3) Apoptosis (^#^4)	**MF**: Activator; **BP**: Oxidation-reduction process	1.9 ± 0.37	1.5 ± 0.26
*** ucp2***	**Mitochondrial uncoupling protein 2**	Cellular Stress (^#^2), Oxidative Stress (^#^3), Apoptosis (^#^4)	**MF**: Oxidative phosphorylation; **BP**: Transport	0.2 ± 0.04	0.2 ± 0.03
**Genes Related to Immune Response**					
*** apod***	**Apolipoprotein D-like**	Immune (^#^5), Apoptosis (^#^4), Oxidative Stress (^#^3)	**MF**: Lipid-binding, Lipid transport; **BP**: Transport, Immunity	4.4 ± 2.73	7.7 ± 2.55
*** c1ql2***	**Complement C1q-like protein 2**	Immune (^#^5), Apoptosis (^#^4)	**MF**: Protein binding; **BP**: Defence response to bacterium	6.4 ± 0.78	12.3 ± 4.47
*** c3***	**Complement C3-like**	Immune (^#^5)	**MF**: Signaling receptor binding; **BP**: Complement alternate pathway, Inflammatory response, Innate immunity	0.6 ± 0.11	0.6 ± 0.12
***camp-a***	**Cathelicidin - paralog a**	Immune (^#^5)	**MF**: Antimicrobial peptide; **BP**: Bacterial defence	0.9 ± 0.2	3 ± 0.89
***casp8***	**Caspase 8**	Immune (^#^5), Apoptosis (^#^4)	**MF**: Hydrolase, Protease, Thiol-protease; **BP**: Apoptosis, Host-virus interaction	1.7 ± 0.12	1.6 ± 0.11
*** ctsh***	**Cathepsin H precursor**	Immune (^#^5), Apoptosis (^#^4)	**MF**: Hydrolase, Protease, Thiol-protease; **BP**: Proteolysis	0.3 ± 0.03	0.3 ± 0.03
*** epx***	**Eosinophil peroxidase-like**	Immune (^#^5), Oxidative Stress (^#^3)	**MF**: Oxidoreductase, Peroxidase; **BP**: Bacterial defence, Oxidative stress response	3.1 ± 0.85	2.3 ± 0.57
***il8 (cxcl8)***	**Interleukin 8 (Chemokine CXC)**	Immune (^#^5)	**MF**: Cytokine; **BP**: Chemotaxis, Inflammatory response	1.4 ± 0.33	2.6 ± 0.73
***irf2***	**Interferon regulatory factor 2**	Immune (^#^5)	**MF**: Activator, DNA-binding, Repressor; **BP**: Transcription regulation	0.8 ± 0.15	0.8 ± 0.17
***mhcii (hla-dra)***	**MHC class ii antigen alpha chain**	Immune (^#^5)	**MF**: Peptide antigen binding; **BP**: Adaptive immunity, Immunity	1 ± 0.16	1.5 ± 0.32
*** mmp9***	**Matrix metalloproteinase 9**	Immune (^#^5), Apoptosis (^#^4)	**MF**: Hydrolase, Metalloprotease, **BP**: Collagen degradation	2 ± 0.54	1.4 ± 0.25
***nckap1l***	**Nck-associated protein 1-like**	Immune (^#^5), Apoptosis (^#^4)	**MF**: Protein kinase activator; **BP**: Apoptosis	0.6 ± 0.05	0.6 ± 0.04
*** tapbp***	**Tapasin**	Immune (^#^5)	**MF**: Peptide antigen binding; **BP**: Immune response, Antigen presentation	0.8 ± 0.17	0.9 ± 0.21
***tnfrsf6b***	**Tumor necrosis factor receptor superfamily member 6b**	Immune (^#^5), Apoptosis (^#^4)	**MF**: Signaling Receptor; **BP**: Apoptosis	1.6 ± 0.45	2.8 ± 0.71
**Genes Related to Cellular Metabolism**					
***gck***	**Glucokinase**	Cellular Metabolic Processes (^#^10)	**MF**: Allosteric enzyme, Kinase, Transferase; **BP**: Glycolysis	0.5 ± 0.25	0.7 ± 0.35
*** pdk3***	**Pyruvate dehydrogenase [lipoamide] kinase isozyme 3**	Cellular Metabolic Processes (^#^10)	**MF**: Kinase, Transferase; **BP**: Carbohydrate metabolism, Glucose metabolism	1.8 ± 0.15	1.3 ± 0.11
**Gene Related to Transcriptional Regulation**					
*** dnmt1***	**DNA (cytosine-5)-methyltransferase 1**	Transcription (^#^7)	**MF**: Chromatin regulator, DNA-binding, Methyltransferase; **BP**: DNA methylation	0.5 ± 0.02	0.5 ± 0.04

^a^ Refers to the identity of the 44 K microarray-identified genes selected for qPCR validation. Gene abbreviations are according to UniProt terminology. Further details about primer sequences and BLASTn hits for gene identification purposes are given in Additional files 10 and 11

^b^ Categories of functional themes as identified in the GO/pathway term network analysis: Heat Shock Response (^#^1), Cellular Stress (^#^2), Oxidative Stress (^#^3), Apoptosis (^#^4), Immune Response (^#^5), Protein Processing & Localization (^#^6), Transcription (^#^7), Proteolysis (^#^8), Catabolic Processes (^#^9) and Cellular Metabolic Processes (^#^10) (see Figs. 3 and 4)

^c^ Key molecular function (MF) and biological process (BP) terms according to UniProt database for each gene

^d^ Mean fold change (FC) ± S.E.M for the Warm & Normoxic (WN) group based on Fluidigm Biomark™ qPCR validation

^e^ Mean fold change (FC) ± S.E.M for the Warm & Hypoxic (WH) group based on Fluidigm Biomark™ qPCR validation

**Table 3:** Temperature and hypoxia treatment effects on the transcript expression of 41 target genes (qPCR validation)

Linear Mixed-Effect Models For Each Target Gene^**a**^					
Gene	Intercept		Treatment		Post-hoc Test (*lsmeans*)
	F-value	***p***-value	F-value	***p***-value	
***apod***	165.5	**< 0.0001**	15.46	**0.026**	CT vs WH
***c1ql2***	1954.6	**< 0.0001**	48.87	**0.005**	CT vs WN, CT vs WH
***c3***	132.1	**< 0.0001**	2.53	0.227	
***calm***	1875.4	**< 0.0001**	84.92	**0.002**	CT vs WN, CT vs WH
***camp-a***	429.4	**< 0.0001**	6.02	*0.089*	
***casp8***	249.8	**< 0.0001**	16.97	**0.023**	CT vs WN, CT vs WH
***cat***	19,704.0	**< 0.0001**	4.54	0.124	
***cirbp***	356.3	**< 0.0001**	84.34	**0.002**	CT vs WN, CT vs WH
***cldn3***	739.8	**< 0.0001**	13.75	**0.031**	WN vs WH
***ctsh***	509.5	**< 0.0001**	76.82	**0.003**	CT vs WN, CT vs WH
***cul3***	1087.0	**< 0.0001**	2.28	0.250	
***cyp1a1***	362.9	**< 0.0001**	30.48	**0.010**	CT vs WN, CT vs WH
***dnmt1***	1240.3	**< 0.0001**	40.65	**0.007**	CT vs WN, CT vs WH
***egln2***	5750.5	**< 0.0001**	76.76	**0.003**	CT vs WN, CT vs WH
***epx***	245.1	**< 0.0001**	19.68	**0.019**	CT vs WN, (*CT vs WH*)
***gck***	189.4	**< 0.0001**	2.47	0.232	
***gstt1***	802.0	**< 0.0001**	18.43	**0.021**	(*CT vs WN*), CT vs WH
***hcn1***	225.1	**< 0.0001**	41.89	**0.006**	CT vs WN, CT vs WH
***hif1α***	394.9	**< 0.0001**	10.88	**0.042**	(*CT vs WN*), CT vs WH
***hsp70***	61.6	**< 0.0001**	3.18	0.181	
***hsp90aa1***	291.6	**< 0.0001**	45.56	**0.006**	CT vs WN, CT vs WH
***hsp90ab1***	255.8	**< 0.0001**	2.05	0.275	
***hspd1***	1795.4	**< 0.0001**	167.12	**0.001**	CT vs WN, CT vs WH
***igfbp2b1***	81.7	**< 0.0001**	1.68	0.323	
***il8***	124.1	**< 0.0001**	3.72	0.154	
***irf2***	403.4	**< 0.0001**	0.89	0.499	
***jak2***	318.8	**< 0.0001**	8.01	*0.053*	
***jund***	166.4	**< 0.0001**	16.66	**0.024**	CT vs WN, CT vs WH
***mhcii***	93.8	**< 0.0001**	1.69	0.322	
***mmp9***	76.1	**< 0.0001**	9.23	*0.052*	
***nckap1l***	126.7	**< 0.0001**	17.66	**0.022**	CT vs WN, CT vs WH
***ndufa1***	4303.5	**< 0.0001**	0.58	0.611	
***ndufa4***	93.1	**< 0.0001**	8.26	*0.060*	
***pdk3***	1163.6	**< 0.0001**	16.22	**0.025**	CT vs WN, (*CT vs WH*)
***prdx6***	1814.2	**< 0.0001**	158.74	**0.001**	CT vs WN, CT vs WH
***rraga***	1624.3	**< 0.0001**	46.90	**0.006**	CT vs WN, CT vs WH
***serpinh1***	54,283.9	**< 0.0001**	31.67	**0.010**	CT vs WN, CT vs WH
***tapbp***	69.2	**< 0.0001**	0.14	0.878	
***tnfrsf6b***	69.6	**< 0.0001**	4.61	0.122	
***txn***	82.9	**< 0.0001**	3.21	0.180	
***ucp2***	409.9	**< 0.0001**	147.53	**0.001**	CT vs WN, CT vs WH
	**Num DF = 1, Den DF = 18**		**Num DF = 2, Den DF = 3**		

^a^ Linear mixed-effect models (LMEs) were performed for each gene individually to assess the effect of the fixed factor ‘treatment’ and included the random term ‘tank’. To investigate pairwise comparisons between the Control (CT), Warm & Normoxic (WN) and Warm & Hypoxic (WH) treatment groups the significant models were followed by *lsmeans* post-hoc tests with Tukey’s multiple comparisons adjustment of *p*-values. Significant values are marked in bold letters (*p* < 0.05) and trends are indicated in italics and/or in brackets (0.05 < *p* < 0.1)

### Gene-by-gene analysis

#### Genes related to heat shock response (^#^1)

Expression of the heat shock response-related genes *serpinh1* (*p* = 0.010; Fig. 5a) and *hsp90aa1* (*p* = 0.006; Fig. 5b, Table 3) was higher in fish challenged with the WN and WH conditions in comparison to the CT group. On the contrary, while the gene *hsp70* was slightly elevated in both treatment groups, this effect was not significant (*p* = 0.181; Fig. 5c, Table 3).

### Genes related to cellular stress (^#^2), oxidative stress (^#^3), transcription (^#^7) and cellular metabolic processes (^#^10)

The stress-related gene *hcn1* was significantly up-regulated in WN and WH fish as compared to CT fish (*p* = 0.006; Fig. 5d, Table 3). In contrast, seven other stress-related genes were significantly down-regulated in both treatment groups as compared to the CT group: *cirbp* (*p* = 0.002; Fig. 5e), *calm* (*p* = 0.002; Fig. 5f), *cyp1a1* (*p* = 0.010; Fig. 5g), *egln2* (*p* = 0.003; Fig. 5h), *prdx6* (*p* = 0.001; Fig. 5i), *rraga* (*p* = 0.006; Fig. 5j) and *ucp2* (*p* = 0.001; Fig. 5k) (Table 3). Two stress- and hypoxia-related genes, *hif1α* (*p* = 0.042; Fig. 5l) and *gstt1* (*p* = 0.021; Fig. 5m), were only significantly down-regulated in the WH group as compared to the CT group (Table 3). The gene *dnmt1*, which is involved in transcriptional regulation, was significantly down-regulated in WN and WH fish in comparison to CT fish (*p* = 0.007; Fig. 5n, Table 3). Finally, the gene *pdk3*, which plays a key role in regulating glucose metabolism, was significantly up-regulated in the WN group (*p* = 0.025; Fig. 5o, Table 3), but did not quite reach significance (*p* = 0.051) in the WH group.

### Genes related to apoptosis (^#^4)

Two genes related to apoptotic processes [*casp8* (*p* = 0.023; Fig. 5p) and *jund* (*p* = 0.024; Fig. 5q)] were more highly expressed in fish challenged with the WN and WH conditions in comparison the CT group, while *jak2* (*p* = 0.053; Fig. 5r) showed a similar trend (Table 3). The gene *ctsh* was significantly down-regulated in WN and WH fish in comparison to CT fish (*p* = 0.003; Fig. 5s, Table 3).

### Genes related to immune response (^#^5)

Three immune-related genes [*apod* (*p* = 0.026; Fig. 5t), *c1ql2* (*p* = 0.005; Fig. 5u) and *epx* (*p* = 0.019; Fig. 5v)] had higher expression levels in the treatment groups as compared to the CT group. However, these genes had different expression profiles (Table 3). The gene *c1ql2* was significantly up-regulated in both experimental groups as compared to the CT group (Table 3). Whereas the expression of *apod* was only significantly higher in the WH group, and the expression of *epx* was only higher in the WN group as compared to the CT group (Table 3). The expression of *nckap1l* was significantly down-regulated in the WN and WH groups as compared to the CT group (*p* = 0.022; Fig. 5w), and this result was contrary to the up-regulation found for the microarray probe (Additional file 8).

### PCA analysis on 24 stress-related (^#^1–4) and 14 immune-related (^#^5) genes

**Fig. 6:**
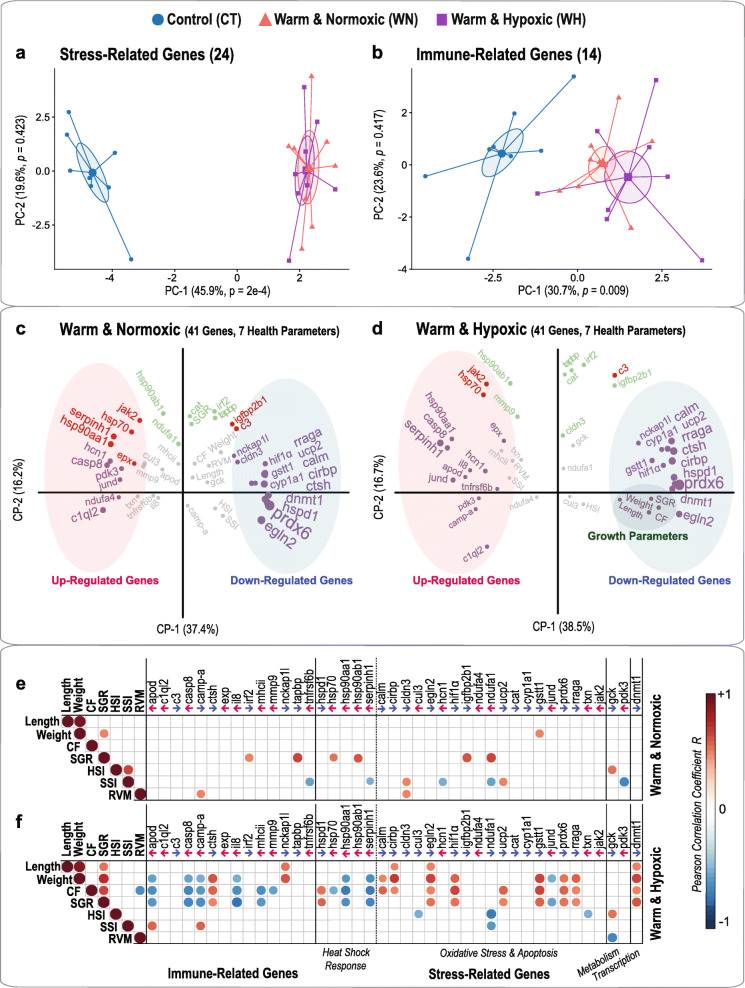
Cluster and correlation analyses between gene expression and phenotypic traits. **a** Principal Component Analysis (PCA) based on 24 stress-related target genes and **b** 14 immune-related target genes. PCAs illustrate the differential gene expression profiles for salmon subjected to Control (CT: 12 °C, 100% air saturation), Warm & Normoxic (WN: 20 °C, 100% air sat.) or Warm & Hypoxic (WH: 20 °C, ~ 70% air sat.) conditions (n = 8 per treatment, N = 24 total). The ellipses denote the dispersion of variance with 95% confidence intervals around the center of the distribution for each treatment group. The variances explained by the Principal Components (PC-1 and PC-2) are indicated in percentage values (%). **c** Component maps for the Warm & Normoxic and **d** Warm & Hypoxic treatments based on 41 target genes and seven phenotypic traits [fork length (length), body mass (weight), condition factor (CF), specific growth rate (SGR), hepato-somatic index (HSI), spleen-somatic index (SSI) and relative ventricular mass (RVM)]. The diameter of the dots and label size reflect the contribution of the response variables (i.e., larger circles and labels indicate a greater contribution). Variables coloured in grey do not contribute significantly to either visualized component. Variables coloured in purple significantly contribute to the first component (CP-1) and variables in green significantly contribute to the second component (CP-2). Variables coloured in red significantly contribute to both CP-1 and CP-2. The variance explained by CP-1 and CP-2 are indicated in percentage values (%). **e** Ordered Pearson correlation matrix for Warm & Normoxic and **f** Warm & Hypoxic exposed fish between the gene expression (RQ) of 41 target genes and seven phenotypic traits. Positive correlations are in red (+ 1) and negative correlations are in blue (− 1), while the intensity of the colour and the size of the dots are proportional to the correlation coefficient. Non-significant correlations (*p* > 0.05) are not shown. The arrows proceeding a gene name indicate whether the gene was up-regulated () or down-regulated () based on the qPCR analysis

The PCA analysis based on the 24 stress-related genes revealed similar transcript expression profiles for salmon exposed to WN and WH conditions in comparison to the CT condition (PC-1: *p* = 2e-4, explaining 45.9% of the variance; CT vs WN *p* = 1e-4, CT vs WH *p* = 2e-4, WN vs WH *p* = 0.537; Fig. 6a and Table 1). A similar pattern was found for the global expression of 14 immune-related genes, even though the WH group displayed a slightly wider dispersion of variance as compared to the WN group (PC-1: *p* = 0.009, explaining 30.7% of the variance; CT vs WN *p* = 0.011, CT vs WH *p* = 0.012, WN vs WH *p* = 0.405; Fig. 6b and Table 1).

### Correlation between gene expression and phenotypic traits

The following seven phenotypic characteristics reported in Gamperl et al. [42] were used for correlation analyses with the qPCR gene expression data (RQs): weight, fork length, condition factor (CF), specific growth rate (SGR), hepato-somatic index (HSI), spleen-somatic index (SSI) and relative ventricular mass (RVM).

### Correlation analysis for WN fish

In the component map based on the response variables obtained for WN fish (Fig. 6c), the target genes segregated according to their responses into 20 up-regulated and 21 down-regulated genes on opposing directions along Component-1 (CP-1: explaining 37.4% of the variance) (Fig. 6c). The following up-regulated genes contributed significantly to the variance explained by CP-1: *c1ql2* (CP-1 = − 6.3), *hsp90aa1* (CP-1 = − 5.3), *casp8* (CP-1 = − 5.2), *serpinh1* (CP-1 = − 5.0), *pdk3* (CP-1 = − 4.3), *jund* (CP-1 = − 3.6), *hcn1* (CP-1 = − 3.6), *jak2* (CP-1 = − 3.1), *hsp70* (CP-1 = − 2.6), *ndufa4* (CP-1 = − 2.3) and *epx* (CP-1 = − 2.3) (Fig. 6c; Additional file 9). On the contrary, the down-regulated genes that had a significant contribution to the variance of CP-1 were: *prdx6* (CP-1 = 11.6), *egln2* (CP-1 = 8.9), *cirbp* (CP-1 = 8.5), *hspd1* (CP-1 = 8.5), *ctsh* (CP-1 = 8.3), *dnmt1* (CP-1 = 7.8), *rraga* (CP-1 = 7.2), *calm* (CP-1 = 6.9), *ucp2* (CP-1 = 5.7), *cyp1a1* (CP-1 = 5.1), *hif1α* (CP-1 = 4.3) and *gstt1* (CP-1 = 4.3) (Fig. 6c; Additional file 9). Since the main treatment effect was explained by PC-1 (Table 1), all of the genes with significant associations to CP-1 are likely to be key drivers of the phenotypic changes in WN fish. Out of the seven phenotypic characteristics for the WN group, only SGR contributed significantly (Fig. 6c; Additional file 9), and six genes showed a positive correlation (R between 0.5 and 0.65) with SGR (*irf2, tapbp, hsp70, hsp90ab1, igfbp2b1* and *ndufa1*; Fig. 6e).

### Correlation analysis for WH fish

In the component map based on the response variables obtained for WH fish (Fig. 6d), 19 up-regulated and 22 down-regulated genes were segregated on opposing directions along CP-1 (CP-1: explaining 38.5% of the variance). For the WH group, we found similar up-regulated genes as compared to the WN group with a significant contribution to the variance explained by CP-1: *serpinh1* (CP-1 = − 6.8), *casp8* (CP-1 = − 4.6), *jund* (CP-1 = − 4.5), *hcn1* (CP-1 = − 4.0), *hsp90aa1* (CP-1 = − 3.2), *hsp70* (CP-1 = − 3.0), *jak2* (CP-1 = − 2.9), *c1ql2* (CP-1 = − 2.7), *epx* (CP-1 = − 2.3) and *pdk3* (CP-1 = − 2.1) (Fig. 6d; Additional file 9). Interestingly, only in WH fish the four up-regulated immune-related genes *apod* (CP-1 = − 2.5), *tnfrsf6b* (CP-1 = − 2.3), *il8* (CP-1 = − 2.1) and *camp-a* (CP-1 = − 2.0) contributed significantly (Fig. 6d; Additional file 9). We identified the same set of high contributing down-regulated genes for WH fish as for WN fish: *prdx6* (CP-1 = 10.3), *rraga* (CP-1 = 8.6), *ctsh* (CP-1 = 8.0), *cirbp* (CP-1 = 7.9), *calm* (CP-1 = 7.4), *hspd1* (CP-1 = 7.0), *egln2* (CP-1 = 7.0), *ucp2* (CP-1 = 6.1)*, dnmt1* (CP-1 = 5.9), *cyp1a1* (CP-1 = 5.2), *gstt1* (CP-1 = 4.6) and *hif1α* (CP-1 = 4.1) (Fig. 6d; Additional file 9). The growth parameters weight (CP-1 = 3.1), CF (CP-1 = 3.1), SGR (CP-1 = 2.7) and length (CP-1 = 2.0) contributed significantly to CP-1 and showed a strong association with down-regulated genes (Fig. 6d; Additional file 9). When using Pearson correlation coefficients, the expression of 12 down-regulated genes (*ctsh, nckap1, hspd1, calm, cirbp1, egln2, hif1α, ucp2, gstt1, prdx6, rraga* and *dnmt1*) had a strong positive correlation (R between 0.51 to 0.81) with growth performance (i.e., weight, length, CF or SGR): the lower the RQ (i.e., the more expression was down-regulated) the greater the reduction in growth (Fig. 6f). On the other hand, seven up-regulated genes (*apod, casp8, camp-a, il8, hsp90aa1, serpinh1* and *jund*) correlated negatively (R between − 0.52 to − 0.73) with these phenotypic characteristics (Fig. 6f). Finally, the expression of the immune-related genes *apod* (R = 0.59) and *camp-a* (R = 0.57) correlated positively with SSI (Fig. 6f).

## Discussion

Seasonal fluctuations in water temperature and oxygen content are unavoidable at aquaculture cage-sites and have profound impacts on the growth, survival and welfare of farmed fish [1, 3, 33, 36, 40, 42]. With global warming, it is predicted that suboptimal temperatures (both high and low) and low water oxygen levels (hypoxia) may become more frequent and severe in coastal areas [7–11], and ultimately more challenging for aquaculture species [46]. However, we still have limited knowledge about the capacity of salmon to tolerate high temperatures in combination with hypoxia, and need to understand how their physiology and immune function are affected. Thus, in this study, Atlantic salmon were subjected to an incremental increase in water temperature (12 → 20 °C; at 1 °C week^− 1^) under normoxia or moderate hypoxia (~ 70% of air sat.), conditions that realistically simulate summer conditions in salmon aquaculture sea-cages [33, 34, 41]. Then, functional genomic approaches were employed (Agilent 44 K salmonid oligonucleotide microarray and Fluidigm Biomark™ qPCR) to assess the hepatic transcriptomic responses to these environmental stressors due to the important role of the liver in numerous biological processes [24, 43]. We report that salmon exposed to incremental warming up to 20 °C, alone and in combination with moderate hypoxia, showed somewhat similar global gene expression responses that were very distinctive from the control (12 °C and normoxic) fish. Overall, we identified a set of 2894 DEPs, out of which 1111 DEPs (38%) were shared between the WH and WN treatment groups. Biological pathway analysis suggested that both treatments increased gene expression with regards to the heat shock response, the unfolded protein response (UPR), endoplasmic reticulum (ER) stress, apoptosis and immune defence. In contrast, a variety of general metabolic processes, proteolysis and oxidation-reduction processes were suppressed. Interestingly, even though the combination of high temperature and moderate hypoxia influenced a number of similar processes, we also identified a unique set of 994 DEPs (34%) that were more strongly dysregulated in WH fish and showed more pronounced impacts on the heat shock response and immune processes. Further, fish exposed to both stressors showed strong correlations between the expression of 19 microarray-identified biomarkers and parameters of growth performance (i.e., weight, length, CF, SGR), which were previously reported to be reduced in these fish [42]. This data reinforces the biological relevance of these genes and pathways, and emphasizes their involvement in phenotypic responses.

### Heat shock response, unfolded protein response and endoplasmic reticulum stress

In stressful conditions, the expression of highly conserved, ubiquitously distributed, molecular chaperones [Heat Shock Proteins (HSPs)] is initiated to maintain cell function and homeostasis, and to protect tissues and cells from structural damage [47–49]. HSPs play a fundamental role in the folding of newly synthesized polypeptide chains, and the refolding and degradation of misfolded proteins to prevent their aggregation and loss of functionality [48, 49]. In addition, they are involved in immune system function (e.g., antigen presentation) [50, 51], apoptosis [52] and protecting cells from oxidative stress [53]. HSPs are essential regulators of the cellular stress response in aquatic ectotherms [54], and their expression is well characterized in teleost species exposed to thermal stress [13–17, 21–23] and hypoxia [26, 29, 30]. After the incremental temperature challenge to 20 °C (alone or in combination with hypoxia), we found enriched pathways related to the heat shock response, protein folding and protein stability (Theme ^#^1) (i.e., ‘chaperone-mediated autophagy processes’, ‘chaperone and protein folding responses’, ‘STIP1(HOP) binds HSP90 and HSP70:HSP40:nascent protein’). Interestingly, we observed a similar magnitude of up-regulation for genes related to chaperone function in WN and WH fish (i.e., *serpinh1, hsp90aa1, hsp90ab1* and *hsp70*) in the microarray. Of these, *serpinh1* (alias *hsp47,* encoding Serpin H1) was one of the most up-regulated target genes (qPCR validated) in comparison to the control group, and this is in agreement with previous studies on over ten fish species [55]. Serpin H1 binds very specifically to collagens and procollagens to facilitate their assembly and stabilization, and plays an important role in collagen biosynthesis [56]. Moreover, Serpin H1 is involved in the breakdown of reactive oxygen species (ROS) produced during oxidative stress as recently shown in rainbow trout (*Oncorhynchus mykiss*) [57]. Hence, the increased expression of *serpinh1* mRNA in the liver may have assisted in the stabilization of collagen molecules within the extracellular matrix (ECM), and further enabled the elimination of ROS and the maintenance of cellular homeostasis during this thermal challenge. Likewise, the expression of the gene *hsp90aa1* (alias *hsp90-alpha,* encoding Heat Shock Protein 90 alpha) which is the inducible form of HSP90, and its constitutive counterpart *hsp90ab1* (alias *hsp90-beta,* encoding Heat Shock Protein 90 beta), were both up-regulated in WN and WH fish as compared to CT fish (although only *hsp90aa1* was qPCR validated). These findings are in line with previous studies reporting higher expression of *hsp90aa1* mRNA, and minor effects on the constitutive expression of *hsp90ab1,* after thermal stress in fish [15, 58]. HSP90AA1 is an abundant molecular chaperone and is implicated in a wide variety of cellular processes including protection of the proteome, the folding and transport of newly synthesized polypeptides, and the activation of proteolysis of misfolded proteins [54, 59, 60]. In our study, *hsp90aa1* was connected with most of the HSP-related GO/pathway terms (e.g., ‘HSF1 activation’ and ‘dissociation of cytosolic HSF1:HSP90 complex’, Figs. 3a and 4a; Additional files 3 and 4), and hence, was an essential component of the unfolded protein response (UPR). Although up-regulation of *hsp70* (Heat Shock Protein 70) in the WN and WH groups was not confirmed by qPCR, it was associated with enriched GO/pathway terms such as ‘HSP70:HSP40:nascent protein’ (Figs. 3a and 4a; Additional files 3 and 4). HSP70 is considered to be a hallmark of the heat shock response, and enhanced transcript expression upon heat stress has been observed in numerous fish species [54, 55]. Thus, in the current study, inducible forms of HSP70 may have been involved in molecular chaperone processes within the liver cells of stress-exposed fish to assist with the folding of nascent polypeptide chains and the repair and degradation of altered or denatured proteins [61]. In addition, we identified similar enriched pathways in WN and WH fish that are important for ER protein processing (Theme ^#^6) (i.e., ‘translational initiation’, ‘protein localization/targeting to ER’), and unique UPR pathways in WH fish, that point to the presence of ER-stress. The ER produces a large number of secretory proteins, and has quality-control systems that ensure the correct folding of proteins and their vesicular cellular transport [62]. The ER-stress response initiates the UPR to prevent the assimilation of unfolded proteins and restore ER function [62].

In summary, we found highly active cellular HSP and chaperone-mediated ER-stress responses in the liver of WN and WH exposed salmon that potentially prevented the accumulation of unfolded proteins and maintained cell homeostasis. However, fish exposed to hypoxia in combination with heat stress had larger and more interconnected clusters associated with the HSP and UPR response (Theme ^#^1) and this indicates that there was a greater induction of these essential cell maintenance processes in the liver of WH fish during this climate change scenario.

### Apoptosis

Apoptosis is a process of programmed cell death that allows for the removal of defective cells without the release of intracellular contents, and prevents local tissue inflammation [63]. High temperature and hypoxia can induce oxidative stress that mediates apoptotic processes in fish tissues [20, 64–67]. Here, we identified similar enriched GO/pathway terms connected to apoptosis (Theme ^#^4) in WN and WH fish that were associated with up-regulated DEGs (i.e., ‘cysteine-type endopeptidase activity involved in apoptotic signaling pathway’ and ‘regulation of nitrosative stress-induced intrinsic apoptotic signaling pathway’). However, the WN fish had more enriched GO/pathways terms related to apoptotic processes, and these had a higher interconnectivity in the functional network (~ 92 terms) as compared to WH fish (~ 10 terms). In addition, WN fish had enriched heat shock response (e.g., unfolded protein binding) and immune (e.g., MHC protein complex binding) pathways that were linked with apoptotic processes. This suggests that enhanced HSP transcript expression (i.e., *hspaa1* and *hspd8*) may have been associated with cell resistance to apoptotic cell death [52].

Nevertheless, in both warming groups, we found several differentially expressed genes from the microarray experiment that were associated with these enriched apoptosis-related pathways (i.e., *casp8, jund* and *jak2*)*.* Amongst them, the gene *casp8* was the most frequently occurring transcript in enriched apoptosis-related GO/pathways (Additional files 3 and 4), and was significantly up-regulated in WN and WH fish as compared to CT fish (and qPCR validated). CASP8 initiates a protease cascade that induces receptor-mediated extrinsic cell death [68]. On the contrary, the significant up-regulation of the transcript *jund* (encoding Transcription factor JunD) in WN and WH fish indicates that anti-apoptotic processes were also activated to protect cells from senescence or apoptosis by acting as a modulator of the p53 signalling cascade [69].

Collectively, these findings suggest that several processes were involved in initiating the apoptosis of cells potentially damaged by oxidative stress in the liver of WN and WH fish, but also that anti-apoptotic pathways likely prevented extensive programmed cell death which could have resulted in hepatic necrosis or impacted liver function [1, 63, 66, 67]. Interestingly, moderate hypoxia combined with high temperature resulted in a less pronounced activation of apoptotic processes (Theme ^#^4) as compared to fish that experienced high temperature alone. At present, we do not have an explanation for this finding.

### Immune response

Several studies have shown that exposure to elevated temperatures [15, 17–19, 22] or hypoxia [6, 70] affects immune-related gene expression in fish. However, these studies focused on the effects of acute or chronic temperature increases, or hypoxia, as individual stressors. Here we report that an incremental increase in temperature under normoxia or moderate hypoxia resulted in the up-regulation of several genes linked to the innate and adaptive immune system (Theme ^#^5) such as *apod, c1ql2, casp8, epx, mhcii, il8, tapbp* and *tnfrsf6b* in the microarray (with *apod, c1ql2* and *casp8* qPCR validated). Interestingly, salmon exposed to moderate hypoxia and high temperature had more up-regulated genes that were associated with enriched GO/pathway terms of the innate immune response (i.e., ‘neutrophil and granulocyte chemotaxis and granulation’, ‘IL-17, IL-4 and IL-13 signalling’), and antiviral responses (i.e., ‘herpes simplex virus 1 infection’). Further, WH fish displayed a more distinct immune-related gene expression profile as compared to CT and WN fish. This shift could be attributed to a stronger differential expression of several genes in WH fish such as *apod, camp-a, c1ql2, il8, mhcii* and *tnfrsf6b*, and some of these correlated strongly with health-related parameters. For instance, *apod* (encoding Apolipoprotein D) was more up-regulated in WH fish as compared to WN fish (WN: 4.4-fold, WH: 7.7-fold; qPCR validated), and was positively correlated with spleen-somatic index. The extracellular glycoprotein APOD has multiple functions that involve the immune response, chemoreception, proteolysis and lipid oxidation [71], and potentially plays a fundamental role in cellular-stress and immune responses. The higher expression of *c1ql2* (encoding C1q-like Protein 2) transcripts (WN: 6.4-fold, WH: 12.3-fold; qPCR validated) suggests that the classical complement system pathway was also activated in these treatment groups. This is an essential innate defence mechanism in teleost fish that detects and destroys invading pathogens by bacteriolysis [72]. For example, acclimation to 20 °C for 57 days increased the lytic activity of the total complement system in the plasma of rainbow trout [73]. Interestingly, the gene *c3* (encoding Complement C3), an important component of the alternative complement pathway, was not significantly affected by exposure to elevated temperatures in the qPCR assessment. Consistent with this finding we found that there was no change in plasma hemolytic activity of the alternative pathway in the same WN and WH fish 24 h after an intraperitoneal injection with bacterial and viral antigens in comparison to CT fish [74]. Oku and colleagues [19] reported that the classical pathway of the complement system was partially activated in the liver of rainbow trout reared at 22 °C vs 14 °C whereas there was no activation of the alternative pathway or induction of final cytotoxic activity. The classical pathway links innate and adaptive immunity as C1q has the ability to bind to aggregated IgG or IgM on immune complexes [75]. Indeed, we found enrichment of ‘MHC complex binding’ processes and a trend for higher expression of *mhcii* (alias *hla-a*, encoding Major Histocompatibility Complex II) in WH fish (WN, 1.0; WH, 1.5-fold; but not qPCR validated). Thus, the applied thermal challenge may have activated adaptive immune responses, on which fish rely more at high temperatures [76]. The GO/pathway term network also indicated that several HSP transcripts were associated with ‘antigen processing and presentation’ (e.g., *hsp90aa1, hsp90ab1, hspa8*) and with ‘MHC class II binding complex’ (e.g., *hsp90aa1* and *hspa8*). HSPs have been shown to act as chaperones for cytosolic peptides involved in MHC-driven antigen presentation to T-lymphocytes [50]. For instance, the cytosolic chaperone HSP90 is associated with peptide binding to MHC-class I and MHC-class II [77, 78]. Consequently, given the observed up-regulation of *hsp90aa1* transcripts in WN and WH fish as compared to CT fish, and this HSP’s relation to ‘antigen processing pathways’, it is possible that MHC peptide complex assembly for antibody production was enhanced [50, 51, 77, 78].

Finally, WN and WH fish had up-regulated genes connected to collagen/ECM degradation (i.e., ‘activation of matrix metalloproteinases’ or ‘collagen degradation by MMPs’) and a trend for higher expression of *mmp9* (encoding Matrix Metalloproteinase 9) (WN, 2.0; WH: 1.5-fold; not qPCR validated). This suggests that these environmental conditions stimulated tissue remodeling processes [79]. Matrix metalloproteinases (MMPs) are endopeptidases that cleave all structural elements of the ECM and are responsible for physiological and pathophysiological tissue remodelling [79, 80]. As such pathological remodeling processes in damaged liver tissues due to apoptosis were likely activated.

In summary, our results suggest that the constitutive expression of immune-related genes was induced to either prepare for more numerous and/or virulent pathogens at warm temperatures [81, 82] as a ‘pre-adaptation’, or to initiate immune defence responses against invading pathogens or existing infections. Further, they show that the combined stressors of high temperature and moderate hypoxia had a greater impact on hepatic immune-relevant transcript expression. However, no significant clinical signs of infection or mortalities were recorded in this experiment [42], and when salmon from both warming scenarios were held at 20 °C for 4 weeks (WN and WH groups) and challenged with a multivalent vaccine (Forte V II; containing both bacterial and viral antigens), their capacity to mount an innate immune response was not impaired and they reached a similar magnitude of antibacterial immune-related gene expression as compared to CT fish [74]. Nonetheless, it is clear that increasing temperatures due to climate change [9] will become more challenging for Atlantic salmon. For example, plasma cortisol levels, which are known to modulate and suppress immune function at higher concentrations [83, 84], were significantly increased (to ~ 30 to 40 ng mL^− 1^) in Atlantic salmon exposed to an incremental temperature elevation to 22 °C (Zanuzzo FS, Peroni EFC, Sandrelli RM, Beemelmanns A, Dixon B, Gamperl AK. The impacts of increasing temperature and moderate hypoxia on the stress physiology of Atlantic salmon (*Salmo salar*) (In prep.)), with approx. 15% mortality at this temperature and 33% when temperatures reached 23 °C [42]. Hence, temperature elevations above 20 °C will likely have a stronger impact on physiological stress and immune competence, and ultimately the disease resistance of Atlantic salmon. Clearly, additional research using live pathogen exposures is needed to determine whether the susceptibility of salmon to infectious diseases is impacted by warmer and more hypoxic environments.

### Oxidative stress

When ectotherms are exposed to warming (hyperthermia) their mitochondrial respiration is increased, and this can result in accelerated mitochondrial ROS formation, oxidative stress and cellular damage [64, 85, 86]. Cellular oxidative stress due to prolonged hyperthermia can cause impaired mitochondrial bioenergetics and structural alterations of cells and tissues [67, 87]. Tolerance to cellular oxidative stress is provided by an effective antioxidant system [86, 88], as well as an HSP response which attempts to maintain protein folding and mitochondrial integrity, and support cell function and survival [52, 53]. In our study, WN fish appeared to have an enhanced oxidative stress response (Theme ^#^3) (i.e., ‘regulation of response to stress’ and ‘response to oxidative stress’) while WH fish showed up-regulated redox-related pathways (i.e., ‘antioxidant activity’, ‘positive regulation of release of cytochrome c from mitochondria’ and ‘thioredoxin reduction’). Thus, the activation of these particular oxidative stress responses, in addition to activated HSP, UPR, and ER-stress responses, appear to be critical in preventing cellular damage and maintaining cell homeostasis during warming.

Surprisingly, all of the measured oxidative stress and hypoxia-sensitive target genes that were selected for qPCR validation (i.e., *cirbp, calm, cyp1a1*, *egln2*, *prdx6*, *rraga*, *ucp2*) were down-regulated in the liver of WN and WH fish at 20 °C as compared to CT fish. Further, some GO/pathway terms connected to cellular oxidative stress (Theme ^#^3) (i.e., ‘oxidoreductase activity’) were associated with down-regulated genes in both warmed groups. Olsvik et al. [14] also reported that the expression of several genes encoding for proteins with an oxidative stress-protective and/or hypoxia sensing function (i.e., *sod1, gr, cyp1a1*, *hif1α*) was significantly reduced in the liver of Atlantic salmon exposed to prolonged high temperature stress (19 °C vs 13 °C for 45 days). In accordance, we also found that the gene *hif1α* (alias *hif-1a*, encoding Hypoxia-Inducible Factor 1 alpha) was significantly down-regulated in WH fish (by 0.57-fold, qPCR validated), while it fell just short of being significant for WN fish (WN: 0.60-fold; *p* = 0.054). The gene *hif1α* encodes for a master hypoxia-responsive transcriptional regulator involved in various cellular processes such as energy metabolism, apoptosis, proliferation and increased oxygen delivery [24, 89, 90]. The expression of *hif1α* is considered to be an evolutionarily conserved hypoxic biomarker due to its up-regulation after acute hypoxia (i.e., hours) in several fish species such as Eurasian perch (*Perca fluviatilis*) [91], Atlantic croaker (*Micropogonias undulatus*) [92] and zebrafish (*Danio rerio*) [28]. However, the data are not as consistent regarding the effects of prolonged hypoxia. For example, while chronic hypoxia induced the up-regulation of *hif1α* transcripts in the ovary of Atlantic croaker (21 days at 55% DO) [92] and in the liver of sea bass (*Dicentrarchus labrax*) (15 days at 51% DO) [93], the expression of this transcript was down-regulated in the muscle and not affected in the liver of perch (15 days at 30% DO) [91]. Further, while this gene’s expression was not altered in the liver of Atlantic salmon exposed to 4–5 mg O_2_ L^− 1^ for 120 days at 12 °C, long-term exposure to 17 °C and 19 °C (45 days) as compared to 13 °C resulted in a lower expression of *hif1α* in the liver of Atlantic salmon [14]. Moreover, Heise [86] showed that while the DNA binding activity of the transcription factor HIF-1 in North Sea eelpout (*Z. viviparous*) liver cells was elevated during mild heat exposure (18 °C), its function appeared to be impaired when this species was exposed to more severe temperature stress (22–26 °C). This author hypothesized that a more oxidized redox state during very high temperatures could interfere (i.e., ‘switch-off’) with the hypoxic signalling response, and thus, prevent the complex HIF-1 induced physiological response. This hypothesis may be supported by the significant down-regulation of *egln2* (alias *phd1*, encoding Egl-9 Family Hypoxia Inducible Factor 2) observed in both warmed groups (qPCR validated) in this study, as it encodes for cellular oxygen sensing enzymes responsible for the post-translational regulation of HIF-1α proteins [94–96]. Three EGL-Nine homologs (EGLN1–3) regulate the abundance of HIF-1α proteins through proline hydroxylation and consequent proteasomal degradation [96], and were shown to be involved in signalling responses in the brain of large yellow croaker (*Larimichthys crocea*) exposed to acute hypoxia [97]. The effects of temperature stress on *egl-9* homolog transcript expression have not previously been reported. Interestingly, we found a down-regulation of *egln2* transcripts in both the WN and WH groups, and this may imply that there was temperature-dependent post-translational regulation of HIF1α in the liver of our Atlantic salmon. In addition, the down-regulation of *calm* (alias *cam*, encoding Calmodulin) in WN and WH fish as compared to CT fish (qPCR validated) suggests that there might have been Ca^2+^/calmodulin kinase-dependent transcriptional regulation of HIF-1 [98]. The Ca^2+^/calmodulin pathway was supressed in the liver of hypoxia tolerant gynogenetic blunt snout bream (*Megalobrama amblycephala*) [31], and thus, may have been important in mediating hypoxia acclimation in salmon. Based on these findings, it is possible that the lower expression of hypoxia sensitive genes (*hif1α, calm, egln2*) in the liver of WH fish may have been caused by: *i*) the moderate level of hypoxia (~ 70% air saturation); *ii*) an acclimation response to prolonged ~ 8–10 weeks of hypoxia/temperature stress; and/or *iii*) a negative feedback loop due to the accumulation of Hif1α proteins. However, further research is needed to gain a better understanding about how the HIF1α pathway in Atlantic salmon is modulated by these two important environmental stressors.

In relation to the above findings, the gene *cyp1a1* which encodes for Cytochrome P450 1A1 was down-regulated in fish from the WN and  WH treatments as compared to CT fish (qPCR validated). Hypoxia and temperature stress can alter transcript levels of CYP1A, which is involved in the oxidation of many substrates and considered to be a vital molecular biomarker for various stressors in the aquatic environment [99]. In line with our results, elevated temperatures (17–18 °C) caused a down-regulation of *cyp1a* mRNA in the liver of Atlantic salmon [14], and chronic hypoxia decreased the expression of this gene in Atlantic cod (six weeks at 46% O_2_ saturation) [100] and Atlantic croaker (four weeks at 1.7 mg DO L^− 1^) [101]. The expression of *ucp2* was also down-regulated in WN and WH fish (qPCR validated) and this gene codes for Mitochondrial Uncoupling Protein 2 (UCP2) which uncouples oxidative phosphorylation from ATP synthesis resulting in energy dissipation [102]. Decreased transcript levels of *ucp2* with increasing temperatures (15–25 °C) were previously reported in the gill and liver of pikeperch (*Sander lucioperca*) [58], and suggest that UCP2 has a thermogenic function [102]. Further, gilthead sea bream (*Sparus aurata*) showed a significant decrease in the expression of *ucp2* in the whole blood after acute (1 h) exposure to 18–20% oxygen saturation [103]. Under stressful conditions, reduced UCP mediated uncoupling (respiration uncoupling) may result in the attenuation of mitochondrial ROS production, and a lower expression of *ucp2* could have been part of a feedback-induced decrease in ROS synthesis for cell protection [104]. Indeed, Gerber and co-workers reported that Atlantic salmon acclimated to 20 °C had reduced cardiac mitochondrial ROS production in comparison to fish acclimated to 12 °C [105]. Thus, alterations in mitochondrial function at high temperatures may be an important mechanism for thermal acclimation and thermal tolerance in this species.

Another highly down-regulated gene in WN and WH fish was *prdx6* (qPCR validated), which encodes for Peroxiredoxin-6. This protein is important for phospholipid homeostasis, lipid peroxidation repair, and inflammatory signalling [106]. Up-regulation of *prdx6* upon heat stress to protect the cell from oxidative stress has been reported in other marine animals [107, 108]. However, while Antarctic emerald rockcod (*Trematomus bernacchii*) exposed to warming temperatures had slightly increased expression of the *prdx6-b* paralog in the liver, expression of the *prdx6-a* paralog was down-regulated [108]. This suggests that *prdx6* paralogs may respond  differently to temperature changes, and that the transcriptional responses of this gene and its paralogs upon warming and exposure to hypoxia deserve further investigation.

Finally, the expression of *cirbp* (which encodes for Cold-Inducible RNA-Binding Protein) was down-regulated to a similar extent in WN and WH fish (qPCR validated), and this transcript was connected with many GO/pathway terms including ‘mRNA stability’ and ‘mRNA catabolic process’. The expression of *cirbp* has been reported to be up-regulated upon cold water exposure, and mild hypoxia, while it is decreased in response to heat stress and chronic hypoxia in vertebrates [55, 109]. The cold-shock protein CIRBP acts as mRNA chaperone, is implicated in multiple cellular processes (i.e., cell proliferation, survival and apoptosis), and is considered to be a general stress-response protein affected by temperature, hypoxia and UV radiation [109]. The lower expression of *cirbp* in this study is in accordance with several heat-stress studies on salmonid fishes [55].

Taken together, the up-regulation and enrichment of pathways related to oxidative stress (Theme ^#^3), HSP-response (Theme ^#^1) and apoptosis (Theme ^#^4) in WN and WH fish suggest that the induction of antioxidant enzymes and redox pathways was an important defense mechanism in these fish. However, the simultaneous down-regulation of several key genes related to the oxidative stress response (Theme ^#^3) also suggests a potential decrease in its effectiveness when salmon are exposed to a slow incremental temperature increase to 20 °C.

### Cellular metabolism

The abiotic factors of temperature and water oxygen level have a profound influence on the allocation of energy to maintenance versus growth in fish [3, 38, 110]. A reduction in metabolic processes can conserve energy during stressful conditions as imposed by thermal challenges [14, 17, 20, 22, 32, 65, 111, 112] or hypoxia [25, 28, 113, 114]. In this study, the expression of genes related to a variety of highly interconnected cellular metabolic processes was suppressed in WN and WH salmon. Amongst many others, the GO/pathway terms of down-regulated genes were connected to aerobic respiration [i.e., ‘tricarboxylic acid cycle (TCA)’], carbohydrate metabolic process (i.e., ‘glucose 6-phosphate metabolic process’), and small-molecule metabolic process (i.e., ‘organic substance biosynthetic process’, ‘fatty acid catabolic process’, ‘lipid metabolic process’). The down-regulation of genes associated with the TCA cycle in the liver of WN and WH fish may indicate a shift from aerobic oxidation to anaerobic glycolysis, as was shown in the Tambaqui (*Colossoma macroppomum*) exposed to predicted IPCC climate scenarios [32]. The decrease in the expression of hepatic *gck* (detected in the microarray), which encodes for the enzyme Glucokinase, suggests that there was also a reduction in glycolytic processes in the liver of WN and WH fish. This result is in agreement with the response of Tambaqui exposed to extreme climate scenarios [112]. In contrast, expression of the gene *pdk3* was up-regulated in WN fish (qPCR validated). This gene codes for Pyruvate Dehydrogenase Kinase 3 (PDK3), which acts together with PDK1, PDK2 and PDK4 isoenzymes to regulate glycolysis and glucose homeostasis during starvation [115].

In our study, WH salmon had reduced food consumption, and a lower feed conversion ratio and growth, as compared to fish of the WN and CT groups [42]. The reduced feed intake and feed conversion efficiency at high temperatures could have had a major impact on the redistribution of energy stores and the amount of glycogen and lipids stored in the liver. Temperature modulates lipid metabolism, and stored lipids in the liver are increasingly used for the maintenance of energy metabolism during thermal stress [38, 110]. For example, Atlantic salmon reared at 17–19 °C for 45 days showed reduced liver lipid and triacylglycerol (TAG) stores as compared to fish maintained at 13 °C, and this suggests that the reallocation and/or depletion of endogenous lipid stores occurs during prolonged high temperature exposure [38]. Furthermore, Atlantic salmon held at 18 °C vs 12 °C for 1 month showed a decline in plasma amino acids (glutamine, tyrosine and phenylalanine) and a decreased lipid status (unsaturated fatty acids, lipids and phospholipids), suggesting that energy stores were mobilized [110]. In the current study, the expression of genes associated with ‘fatty acid and lipid metabolic processes’ was lower in WH fish as compared to CT fish, and thus, it appears that long-term exposure to high temperatures and hypoxia may result in reduced lipid and fatty acid biosynthesis. This hypothesis would be consistent with a recent study on rainbow trout exposed to an incremental temperature increase to 24 °C. These fish showed a temperature-dependent down-regulation of hepatic genes related to energy metabolism [20]. Thus, a down-regulation of pathways related to carbohydrate, protein and fatty acid metabolism in the liver of stressed salmon may reflect the suppression of metabolic processes, and agrees with the important role played by the liver in cellular metabolism and biosynthetic activities in fishes [24, 38, 43].

During hypoxia, metabolic responses to ensure cell survival involve readjustments that decrease ATP demands to match the reduced capacity for ATP production [24]. Moreover, prolonged temperature stress and low oxygen reduce protein synthesis, and this leads to reduced growth and metabolic depression in Atlantic salmon [14]. These findings are consistent with our results. We found positive correlations between the decreased expression of 12 down-regulated genes (e.g., *cirbp, calm, egln2, hif1α, ucp2, gstt1, prdx6, rraga,* etc.) and reduced growth performance (i.e., weight, length and SGR) predominantly in WH fish [42], and this association highlights the biological relevance of these hepatic transcriptional responses.

Collectively, these findings suggest that the combination of high temperature stress and moderate hypoxia resulted in transcriptional responses in the liver that contributed to metabolic suppression in our salmon. This metabolic suppression may have been at least partially needed to balance the energetically costly processes that were invoked to maintain cell homeostasis (i.e., HSP, UPR, ER-stress and apoptosis).

### Transcriptional regulation and epigenetic mechanisms

Temperature stress in the WH and WN groups induced a similar down-regulation of the gene *dnmt1* as compared to CT fish (qPCR validated), and this gene codes for DNA (cytosine-5)-methyltransferase, an enzyme essential for maintaining DNA methylation marks after mitosis [116]. DNA methylation is an important epigenetic regulatory mechanism of transcription (Theme ^#^7), and down-regulation of *dnmt1* indirectly suggests that genome-wide changes in DNA methylation levels may have been involved in regulating these large-scale gene expression responses (i.e., ~ 2894 DEPs). Indeed, Beemelmanns et al. [117] found that the same treatments (i.e., WH and WH) affected the methylation of CpG sites of the microarray-identified genes related to temperature stress (*serpinh1, cribp*), oxidative stress (*prdx6, ucp2*), apoptosis (*jund*) and metabolism (*pdk3*). Several of these changes in CpG methylation were highly correlated with the transcript expression changes reported here, and thus, reinforce their importance as ‘epimarkers’ that regulate transcription upon temperature and hypoxic stress in Atlantic salmon [117].

## Conclusions

We identified numerous transcriptional changes (i.e., 2894 DEPs) in the liver of Atlantic salmon exposed to an incremental temperature increase (12 → 20 °C; at 1 °C week^− 1^) alone or combined with moderate hypoxia (~ 70% of air sat.); the latter simulating summer conditions in salmon aquaculture sea-cages [33, 34, 41]**.** Both these treatments induced biological processes related to the maintenance of cellular homeostasis (i.e., HSP, UPR, ER-stress response) and the apoptosis of damaged cells, and stimulated immune gene expression (both innate and adaptive); but also compromised the oxidative stress response and led to a reduction in the expression of a variety of genes related to metabolic and proteolytic processes. Importantly, we identified a unique set of 994 DEPs (34%) that were strongly dysregulated in WH fish, and showed that this condition had a more distinct and pronounced impact on the heat shock response and immune processes (e.g., more up-regulated genes that were associated with enriched GO/pathway terms of the innate and antiviral immune responses) as compared to that seen for WN fish.

Transcriptomic techniques allow for the high-throughput identification of genes that are sensitive to particular conditions, and can be used as biomarkers for the detection and quantification of stress levels and stress tolerance. In this context, the development of diagnostic biomarkers for quantifying the impact of environmental stressors on an organism’s physiology and health has received increased attention by the aquaculture industry and for use in ecological surveys [55, 58, 118]. Our results agree with recent studies which show that the genes *serpinh1*, *hsp90aa1* and *cirbp* are reliable molecular biomarkers for the detection and quantification of thermal stress in salmonids [55, 58, 119]. However, we found that the expression of *hsp70* was very variable between individuals, and in accordance with previous findings, should not be considered as a stress biomarker alone [120].

We also report that the marked differential expression of 19 microarray-identified genes upon high temperature and hypoxia exposure showed strong associations with important phenotypic characteristics. Hence, these genes may not only be useful as molecular biomarkers of thermal stress, but also as candidate genes for the development of thermal phenotype-relevant genetic markers [e.g., single nucleotide polymorphisms (SNPs) for marker-assisted selection of heat stress resistant broodstock], protein-based diagnostic assays [e.g., Enzyme-linked Immunosorbent Assay (ELISA)] and for the detection of epigenetic markers (‘epimarkers’) that can predict thermotolerance [117].

## Methods

This experiment was performed as part of the ‘Mitigating the Impacts of Climate-Related Challenges on Salmon Aquaculture (MICCSA)’ project, and a detailed description of the experimental protocol and of the data on growth characteristics and mortality are published in Gamperl et al. [42]. All experimental procedures described herein were approved by the Institutional Animal Care Committee of Memorial University (Protocol ^#^16–90-KG) and followed guidelines of the Canadian Council on Animal Care. All sections of this study adhere to the ARRIVE Guidelines for reporting animal research [121].

### Animal husbandry

The experiment was performed from March to August 2017 at the Laboratory for Atlantic Salmon and Climate Change Research (LASCCR), Memorial University, St. John’s, NL, Canada. Post-smolt Atlantic salmon (~ 1.5 years old) of Saint John River (NB, Canada) origin obtained from Northern Harvest Sea Farms Ltd. were implanted with Passive Integrated Transponder (PIT) tags (Loligo® Systems ISO 11784 certified, Viborg, Denmark), then randomly distributed into six 2.2 m^3^ circular indoor fiberglass tanks receiving seawater (32 ppt salinity) at 15 L min^− 1^. The fish were initially acclimated for four weeks under optimal conditions (~ 100–110% air saturation, 12 °C, 32 ppt salinity, 14 h light: 10 h dark photoperiod) and fed a ration of 1% body weight day^− 1^ with a commercial salmon feed (5 mm, EWOS Dynamic S, EWOS Canada Ltd., Surrey, BC, Canada).

### Experimental protocol

Two tanks with 60 fish per tank (average mass 137.6 ± 1.3 g; mean ± S.E.), were randomly assigned to each of three groups as shown in Fig. 1: (1) CT, constant temperature of 12 °C and ~ 100–110% air sat. for the duration of the experiment; (2) WN, incremental temperature increase (12 → 20 °C at 1 °C week^− 1^) at ~ 100–110% air sat.; and (3) WH, decrease in oxygen content to ~ 70% air sat. (daily range ~ 65–75%) over one week, followed by two weeks of acclimation to this oxygen level, and then the same temperature regimen at ~ 70% air sat. The weekly temperature increases in the tanks of the WN and WH treatment groups were 0.3 °C (from days 1 to 3), 0.1 °C on day 4, and then no change from days 5–7. The temperature and dissolved oxygen level in the tanks were monitored daily (YSI, ProODO, Yellow Springs, OH, USA), and ammonia and nitrite levels in the tanks were measured weekly (LaMotte test kits, Chestertown, MD, USA). During the experiment, the salmon were carefully fed by hand to satiation twice daily (at 9:00 and 15:00) with the same commercial salmon feed. See Gamperl et al. [42] for more specific information on these experimental protocols.

In the current study, we sampled eight salmon per treatment group after the fish were exposed to 20 °C for three days (four fish per tank replicate, *n* = 8 per treatment, *N* = 24 fish total). The number of 24 samples was considered sufficient to achieve statistical robustness and power (80%) to detect a significant effect (*p* < 0.05) with an estimated medium-large effect size (f^2^ = 0.43) [122]. All the fish used in this study were euthanized in aerated seawater containing 0.4 g L^− 1^ of MS-222 (tricaine methanesulphonate; Syndel Laboratories, Nanaimo, BC, Canada) followed by cerebral concussion. Fish were dissected and 200–300 mg of liver tissue was collected, flash-frozen in liquid nitrogen and then stored at − 80 °C. The fish’s weight [g], fork length [cm], liver mass [g], spleen mass [g] and ventricle mass [g] were measured, and CF, SGR, HSI, SSI and RVM indices were calculated. These growth and physiological measurements are reported in Gamperl et al. [42] and were used in the current study to perform correlation analyses with the gene expression data.

### RNA extraction, DNase treatment and column purification

For RNA extraction, 100 mg of liver tissue was disrupted and homogenized in 800 μL of QIAzol-Lysis Reagent (QIAGEN, Germantown, MD, USA) for 2 min at 20 Hz using a TissueLyzerII with 5 mm stainless steel beads (QIAGEN, Mississauga, ON, Canada) according to the manufacturer’s instructions. To remove lipid/protein contamination, we performed an additional precipitation step according to the protocol of Xu et al. [123] with minor changes. Briefly, crude RNA samples (100 μg) were mixed with an equal volume of Acid-Phenol:Chloroform (5:1 solution, pH 4.5, Ambion/Life Technologies, Waltham, MA, USA) and centrifuged at 17,000 g and 7 °C for 20 min. Then, 190 μL of the aqueous phase was precipitated with 0.1 volumes of 3 M sodium acetate (pH 5.5; Invitrogen/Thermo Fisher, Vilnius, Lithuania) and 2.2 volumes of ice-cold 100% ethanol (Greenfield Global, Brampton, ON, Canada) at − 80 °C for 12 h, and centrifuged at 17,000 g and 7 °C for 30 min. The RNA pellets were then washed in 1 ml of 70% ethanol, centrifuged at 17,000 g and 7 °C for 20 min, air-dried at room temperature for 10 min, and dissolved in 100 μL of nuclease-free water (Invitrogen/Life Technologies, Grand Island, NY, USA) at 55 °C for 5 min. To remove any remaining genomic DNA, 40 μg of precipitated RNA was incubated for 10 min with 6.8 Kunitz units of DNase I (RNase-Free DNase Set, QIAGEN, Mississauga, ON, Canada) and 1 × of the manufacturer’s buffer. DNase-treated RNA samples were then column-purified using the RNeasy Mini Kit (QIAGEN, Hilden, Germany) according to the manufacturer’s guidelines. RNA integrity was tested with gel electrophoresis (1% agarose) and RNA purity and yields were measured by A260/280 and A260/230 NanoDrop UV spectrophotometry (NanoDrop, Wilmington, DE, USA). All column-purified samples had A260/280 ratios between 2.0 and 2.2 and A260/230 ratios between 1.9 and 2.3.

### Microarray protocol

#### Microarray experimental design and hybridization

Six individual samples from each treatment (three per replicate tank; *n* = 6, *N* = 18 total) were selected to perform a transcriptome microarray study using a common reference design. This sample size number was based on prior microarray studies by our group [70, 124–126], and sample-size and power calculations for microarrays using the *sizepower* function of the *Bioconductor* package in the R-software [127]. Given the experimental design, we estimated that the power of our statistical analysis was 88%, and that we had the ability to detect ~ 2000 DEGs.

An Agilent 44 K Atlantic salmon oligonucleotide array platform (GPL11299; Agilent Technologies, Mississauga, Canada), developed by the consortium for Genomic Research on All Salmonids Project (cGRASP) [44], was used as described in Xue et al. [126] according to the MIAME guidelines [128]. Anti-sense amplified RNA (aRNA) was in vitro transcribed from 1 μg of column-purified RNA using Ambion’s Amino Allyl MessageAmp II aRNA Amplification kit (Invitrogen/Life Technologies, Carlsbad, CA, USA) according to the manufacturer’s instructions. Thereafter, a common reference pool was generated by combining aRNA from all 18 samples (10 μg from each sample), and 20 μg of aRNA per individual sample or reference was precipitated and re-suspended in coupling buffer. The resulting aRNA was labelled with either Cy5 (individual samples) or with Cy3 (common reference) (GE HealthCare, Mississauga, ON, Canada) through a dye-coupling reaction, following the manufacturer’s instructions. The labelling efficiency and concentration of aRNA were determined using spectrophotometry (*microarray* function of NanoDrop), and the efficiencies ranged between 40 and 60 dye molecules per 1000 nt for all samples. Thereafter, 825 ng of labelled aRNA per sample was mixed with an equal amount of labelled common reference aRNA, and the resulting pools were fragmented and co-hybridized to 44 K microarrays at 65 °C for 17 h with 10 rpm rotation using an Agilent hybridization oven (Agilent, Mississauga, ON, Canada). After incubation, the array slides were washed according to the manufacturer’s instructions and dried through centrifugation at 200 g for 5 min at room temperature.

#### Microarray data acquisition

Each 4 × 44 K Agilent microarray slide was scanned at 5 μm resolution with 90% of laser power using a ScanArray Gx Plus scanner and ScanArray Express software (v4.0; Perkin Elmer, Woodbridge, ON, Canada). The Cy3 and Cy5 channel photomultiplier tube (PMT) settings were manually adjusted to balance the fluorescence signal between channels and the four arrays on each slide. The extraction of the resulting raw fluorescence intensity data stored in the TIFF images was performed with Imagene 9.0 (BioDiscovery, El Segundo, CA, USA). Quality controls (background correction and removal of low-quality/flagged spots) and data transformation (log_2_-transformation and Loess normalization) were performed in R using *mArray* from the *Bioconductor* package [129], and with scripts adapted from Booman et al. [130] and Xue et al. [126]. Microarray probes that were absent (non-detected) in more than 25% of the arrays were omitted, and missing values from these undetected probes were imputed using the *LSimpute* package in R [131].

#### Microarray data analysis

To identify significantly up- or down-regulated genes in response to the WN or WH treatments as compared to the CT, we applied a permutational SAM test with a false discovery rate (FDR) of < 5% [132] using the implemented *siggenes* function of the *Bioconductor* package in R [133]. The SAM-identified DEPs were re-annotated using the contiguous sequences (contigs) which contain the 60mer oligonucleotide probe sequences [44]. We performed BLASTx searches of contig sequences against the Swiss-Prot database and BLASTn searches of 60mer probes against the NCBI nr/nt database with E-value < 1 × 1e-5 as filter criteria as described in Umasuthan et al. [124]. The probe annotations were also updated by homology sequence searches in genome annotation databases for *S. salar* and *O. mykiss*, and the assignment of gene symbols for the microarray probes was performed with HUGO Gene Nomenclature Committee (HGNC; https://www.genenames.org/) and/or GeneCards (https://www.genecards.org/) databases according to Umasuthan et al. [124].

A hierarchically clustered heat map was generated with normalized log_2_ ratios for 2894 SAM-identified DEPs (FDR < 5%) using the *pheatmap* R package [134] and applying Pearson correlations and Ward’s agglomerative linkage method (ward.D2) as a cluster algorithm.

### Functionally organized GO/pathway term network analysis

GO/pathway term enrichment and network analyses were conducted for up- and down-regulated DEGs from the WN or WH probe lists (i.e., gene symbol annotation of SAM identified DEPs) using the ClueGO plugin in Cytoscape (v3.5.1) [45]. However, only non-redundant and annotated DEGs were recognized and included (WN group: 377 up-regulated DEGs and 798 down-regulated DEGs; WH group: 735 up-regulated DEGs and 592 down-regulated DEGs). To identify enriched gene clusters related to the WN and WH treatments, an enrichment/depletion analysis was performed using a two-sided hypergeometric test and the Benjamini-Hochberg *p*-value correction method [135]. The genes were mapped to GO-term databases for biological, cellular, molecular and immune processes [136] as well as KEGG [137] and Reactome pathway databases [138] in August 2019. Four functionally organized GO/pathway term networks were created with Kappa-statistics (threshold of 0.4), GO-term fusion strategy, and a medium specificity level to minimize the complexity. Due to discrepancies in complexity between the networks, we selected a *p*-value cut-off of *<* 0.05 for less complex networks (i.e., up-regulated DEGs), while a lower cut-off of *p <* 0.005 was applied for highly complex networks (i.e., down-regulated DEGs). The enriched terms for each functional group were ranked based on their significance level, and the most significant terms were illustrated as a summary label in the produced networks. The determined GO/pathway term groups were categorized into the following functional themes: Heat Shock Response (^#^1), Cellular Stress (^#^2), Oxidative Stress (^#^3), Apoptosis (^#^4), Immune Response (^#^5), Protein Processing & Localization (^#^6), Transcription (^#^7), Proteolysis (^#^8), Catabolic Processes (^#^9) and Cellular Metabolic Processes (^#^10). Finally, the non-redundant significantly enriched GO-terms of biological processes were summarised using the REVIGO cluster algorithm [139] and visualized with dot plots.

### qPCR protocol

#### Gene selection and primer design

The selection of 41 microarray-identified GOIs for validation purposes included significantly up- and down-regulated DEGs that were associated with important functional themes determined by the aforementioned GO/pathway term network analysis (^#^1–5, ^#^7, ^#^10; Table 2). We designed paralog-specific qPCR primers according to specifications detailed in Caballero-Solares et al. [125] using the Primer3web platform (v4.1.0; http://bioinfo.ut.ee/primer3/). Details on qPCR primer sequences, accession numbers, amplicon sizes and amplification efficiencies are represented in Additional file 10. To identify existing paralogs, and to verify the identity of each GOI, we performed BLASTn searches against the non-redundant nucleotide (nr/nt) and the expressed sequence tag (EST) databases of NCBI (year: 2018) (Additional file 11). For nine GOIs (*camp-a, cat, cyp1a1, epx, gck, hif1α, hsp70, il8* and *mmp9*) the paralog-specific primer sequences were obtained from previous studies [140, 141] or the Genome Canada Funded Genomic Applications Partnership Program (GAPP) project ^#^6604 (Biomarker Platform For Commercial Aquaculture Feed Development) database, given that they were targeting the identical probe sequences (Additional files 10 and 11).

#### cDNA synthesis and primer efficiencies

First-strand cDNA templates for qPCR were synthesized in 20 μL reactions from 1 μg of DNaseI-treated, column-purified, total RNA with the QuantiTect®Reverse - Transcription Kit (QIAGEN, Mississauga, ON, Canada) following the manufacturer’s protocol. qPCR primer quality testing was conducted according to previously published protocols [125, 126, 130, 142]. For the evaluation of primer performance and amplification efficiencies for each primer pair, a 5-point 3-fold serial dilution standard curve was generated using cDNA template pools from six samples of the CT and WH treatment groups (*n* = 6, *N* = 12 total). No-template controls (NTC) were included to test for contamination and primer dimers. The 384-well format ViiA 7 Real-Time PCR system (Applied Biosystems/Life Technologies, Foster City, CA, USA) was used to perform amplification reactions in duplicate. qPCR amplifications were completed in 13 μL reactions with 1 × Power SYBR Green PCR Master Mix (Applied Biosystems/Life Technologies), 50 nM of each of the forward and reverse primers, and cDNA representing 10 ng of input total RNA as a starting point of the serial dilution. We applied the following real-time program: 1 cycle of 50 °C for 2 min, 1 cycle of 95 °C for 10 min, and 40 cycles of 95 °C for 15 s and 60 °C for 1 min, including fluorescence detection at the end of each 60 °C step followed by a dissociation curve. Only primer pairs that had efficiencies between 84 and 108% (Additional file 11), and that generated an amplicon with a single melting curve without primer dimers were included for qPCR assays. All amplicons were examined through electrophoresis on 2% agarose gels along with a 1 Kb Plus DNA Ladder (TrackIt™, Invitrogen/Thermo Fisher, Carlsbad, CA, USA) to verify that the correct size fragment was amplified.

#### Selection of normalizer genes

Six normalizer genes [60S ribosomal protein L32 (*rpl32*, BT043656), eukaryotic translation initiation factor 3 subunit D (*eif3d,* GE777139), elongation factor 1-alpha 2 (*ef1a12,* BT058669), polyadenylate-binding protein 1 (*pabpc1*, EG908498), tubulin beta-2C chain (*tubb2c*, XM_014212152) and ATP binding cassette sub-family F member 2 (*abcf2*, BT071904)] from previous salmon transcriptome studies were selected as candidates [125, 126, 142]. The fluorescence threshold cycles (C_T_) of eight samples per treatment group (*n* = 8, *N* = 24 in total) were measured for these six normalizer genes following the previously described method with cDNA representing 5 ng of input total RNA. To identify the most stable normalizers, we conducted a geNorm analysis with the qBase+ software [143] on the C_T_ values obtained from the 24 samples. Based on this analysis, *eif3d* (geNorm M = 0.230), *pabpc1* (geNorm M = 0.232) and *rpl32* (geNorm M = 0.250) were identified as a stable combination (geNorm V = 0.108), and thus, included as normalizer genes in the following qPCR measurements for all experimental samples.

#### qPCR measurements (Fluidigm Biomark™)

The relative transcript expression values of 41 GOIs and the three previously evaluated normalizer genes were assessed for eight individual samples from each treatment group (n = 8, N = 24 in total) using the real-time qPCR Fluidigm Biomark™ HD system (Fluidigm, South San Francisco, CA, USA) based on 96.96 Dynamic Array™ IFC (GE-arrays) according to a protocol developed by Beemelmanns and Roth [144]. Firstly, a pre-amplification step was conducted for each sample by mixing 0.5 μL of a 500 nM STA primer pool (50 μM primer pair mix) with 2.5 μL of TaqMan-PreAmp Mastermix (Applied Biosystems, Waltham, MA, USA), 0.7 μL of nuclease-free water and 1.3 μL of cDNA (representing 200 ng of input total RNA), and using the following thermo-cycle protocol: 10 min at 95 °C; 14 cycles of (15 s at 95 °C, 4 min at 60 °C). Then, the obtained PCR amplicons were diluted 1:10 with low EDTA-TE buffer. For the sample mix preparation, we combined 3.3 μL of pre-amplified cDNA with 3.5 μL of 2 × SsoFast EvaGreen Supermix with Low ROX (Bio-Rad Laboratories, Hercules, CA, USA) and 0.35 μL of 20 × DNA-Binding Dye Sample Loading Reagent (Fluidigm). The primer assay mix was prepared with 0.7 μL of 50 μM primer pair mix, 3.5 μL of Assay Loading Reagent (Fluidigm) and 3.15 μL of low EDTA-TE buffer. In a final step, 5 μL of each sample and assay mix was loaded on the 96.96 GE-array and fluorescence was measured using the Biomark™ HD system by running the GE Fast 96 × 96 PCR + Melt v2 thermal cycling protocol according to the manufacturer’s instructions (Fluidigm). Transcript levels of the 44 GOIs were measured in two technical replicates, and we included two NTCs, two controls for genomic DNA contamination (no-reverse transcription), and two linker samples for inter- and intra-run calibrations.

### qPCR data acquisition

For each technical replicate and sample, the mean threshold cycle (C_T_), standard deviation (SD), and the coefficient of variation (CV) were calculated. As a quality control, C_T_ values with a CV ratio greater than 4% [145] were removed from the dataset due to potential measurement errors. We performed geNorm analysis with the qBase+ software [143] based on the C_T_ values of the three normalizer genes (*eif3d, rpl32, pabpc1*) [125, 126, 142] from all experimental samples. According to the geNorm analysis, the two normalizer genes *rpl32* (geNorm M = 0.302) and *eif3d* (geNorm M = 0.313) were the most stable combination (geNorm V = 0.115). Based on the C_T_ values obtained by the qPCR Fluidigm Biomark™ method, the two normalizer genes were stably expressed and showed mean C_T_ differences below 0.438 ± 0.197 (mean ± SD) when comparing CT vs the WN or WH groups. Finally, the RQs of each gene were determined through normalization to the geometric mean (C_T_ values) of *rpl32* and *eif3d* expression, including the amplification efficiencies (Additional file 10), and setting the sample with the lowest normalized expression level as the calibrator sample (RQ value = 1.0) [143]. The corresponding FC values were calculated for each GOI by dividing its RQs by the mean of the control group.

### Statistical analyses

#### Principal component analysis (PCA)

All statistical tests were performed, and figures generated, in the R environment (v. 3.5.1) [146]. We applied multivariate statistical approaches to infer differences between the transcriptomic expression (log_2_ ratios of microarray) of fish from the CT, WN and WH treatment groups based on: *i*) 17,072 detected probes; *ii*) 1111 DEPs shared between the WH and WN groups; *iii*) 789 DEPs specific for the WN group; and *vi*) 984 DEPs specific for the WH group. In addition, we assessed differential transcript expression patterns of qPCR genes (RQ values) grouped in broader categories of: *i*) 24 stress-related target genes (Themes ^#^1–4); and *ii*) 14 immune-related target genes (Theme ^#^5) (Table 2). To explore differential gene expression patterns based on all of the above-mentioned categories, we performed PCAs for graphical visualization using the *dudi.pca* function of the *ade4* package in R [147]. For each PCA, the first two principal components (PC-1, PC-2) were plotted to obtain a projection of the whole dataset onto a small dimension and to account for the most relevant variance [148]. Then, the scores of PC-1 and PC-2 were extracted, and we fitted linear mixed-effect models for both PCs by applying the *lme* function implemented in the *nmle* package in R [149]. Statistical models were computed with the fixed interaction term ‘treatment’ and the random term ‘tank’ to account for between-tank variation (i.e., tank effect). Each model fit was graphically examined (histogram, qqplots) and residuals tested for normality (Shapiro–Wilk, *p <* 0.05). Finally, significant models were followed by Least-squares means post-hoc tests with Tukey’s *p*-value correction for multiple comparisons by applying the *lsmeans* function in R [150].

#### Gene-by-gene analysis

Differences in transcript expression between treatment groups were determined for each gene by fitting linear mixed-effect models and least-squares means post-hoc tests as explained above. Each model was graphically examined (hisImproved mitochondrial function in salmon togram, qqplots), model residuals were tested for normality (Shapiro–Wilk, *p <* 0.05), and RQ values were log_2_ transformed. Seven outliers were removed (Bonferroni Outlier Test, *p <* 0.05) to fulfill assumptions.

#### Correlation analyses

First, component maps with the epPCA.inference.battery command of the package InPosition in R were computed [151] to: *i*) identify target genes of the highest importance; *ii*) relate the responses between genes; and to iii) assess the relationship between the expression of all 41 target genes and seven phenotypic traits (length, weight, CF, SGR, HSI, SSI and RVM). The incorporated battery of inference permutation tests calculated the component scores for each variable obtained by bootstrap ratios that are visualized in component maps [151]. To validate the gene expression results of the microarray and qPCR analyses, we correlated the mean log_2_ FC values of the 41 target genes using Pearson correlations. Finally, to identify significant correlations between the expression (RQ) of the 41 genes and the seven phenotypic traits, we calculated a Pearson correlation matrix using the *corrplot* function in R [152].

## Supplementary Information

**Additional file 1 **Results of Significance Analysis of Microarrays (SAM, FDR < 5%). Listed are the fold change (FC) values for significantly differentially expressed probes (DEPs) that were identified in the liver of Atlantic salmon exposed to (A) high temperature and normoxia (WN: 20 °C, 100% air sat.) or (B) high temperature and hypoxia (WH: 20 °C, ~ 70% air saturation) as compared to control conditions (CT: 12 °C, 100% air sat.) (*n* = 6, *N* = 18 total).

**Additional file 2 **Enrichment GO-term dot plot for up- and down-regulated differentially expressed genes (DEGs) in Atlantic salmon that were subjected to Warm & Normoxic (WN: 20 °C, 100% air sat.) or Warm & Hypoxic (WH: 20 °C, ~ 70% air sat.) conditions. The dot plots represent non-redundant significantly enriched Gene Ontology (GO) terms of biological processes after application of REVIGO’s redundancy elimination algorithm for (a) up-regulated DEGs of the Warm & Normoxic group; (b) up-regulated DEGs of the Warm & Hypoxic group; (c) down-regulated DEGs of the Warm & Normoxic group; and (d) down-regulated DEGs of the Warm & Hypoxic group. The colour scheme corresponds to the log_10_ adjusted *p*-values (Benjamini and Hochberg method), and the diameter of the dots represents the number of DEGs that were identified to be significantly associated with this specific term.

**Additional file 3 **Results of GO/pathway term network analysis (ClueGO) associated with up-regulated differentially expressed genes (DEGs) of the Warm & Normoxic (WN) treatment group. Listed are the significantly enriched Gene Ontology (GO) terms, and KEGG or Reactome pathways (hypergeometric test *p <* 0.05 with Benjamini-Hochberg correction). GO/pathway term annotations were obtained using the GO database for biological process, cellular component, molecular function, and immune processes, and the KEGG and Reactome pathway databases. The enriched GO/pathway terms are ordered according to ten functional themes: Heat Shock Response (^#^1), Cellular Stress (^#^2), Oxidative Stress (^#^3), Apoptosis (^#^4), Immune Response (^#^5), Protein Processing & Localization (^#^6), Transcription (^#^7), Proteolysis (^#^8), Catabolic Processes (^#^9) and Cellular Metabolic Processes (^#^10). The enriched GO/pathway terms are illustrated in corresponding network Fig. 3a.

**Additional file 4 **Results of GO/pathway term network analysis (ClueGO) associated with up-regulated differentially expressed genes (DEGs) of the Warm & Hypoxic (WH) treatment group. Listed are the significantly enriched Gene Ontology (GO) terms, and KEGG or Reactome pathways (hypergeometric test *p <* 0.05 with Benjamini-Hochberg correction). GO/pathway term anotations were obtained using the GO database for biological process, cellular component, molecular function, and immune processes, and the KEGG and Reactome pathway databases. The enriched GO/pathway terms are ordered according to ten functional themes: Heat Shock Response (^#^1), Cellular Stress (^#^2), Oxidative Stress (^#^3), Apoptosis (^#^4), Immune Response (^#^5), Protein Processing & Localization (^#^6), Transcription (^#^7), Proteolysis (^#^8), Catabolic Processes (^#^9) and Cellular Metabolic Processes (^#^10). The enriched GO/pathway terms are illustrated in corresponding network Fig. 4a.

**Additional file 5 **Results of GO/pathway term network analysis (ClueGO) associated with down-regulated differentially expressed genes (DEGs) of the Warm & Normoxic (WN) treatment group. Listed are the significantly enriched Gene Ontology (GO) terms, and KEGG or Reactome pathways (hypergeometric test *p <* 0.05 with Benjamini-Hochberg correction). GO/pathway term annotations were obtained using the GO database for biological process, cellular component, molecular function, and immune processes, and the KEGG and Reactome pathway databases. The enriched GO/pathway terms are ordered according to ten functional themes: Heat Shock Response (^#^1), Cellular Stress (^#^2), Oxidative Stress (^#^3), Apoptosis (^#^4), Immune Response (^#^5), Protein Processing & Localization (^#^6), Transcription (^#^7), Proteolysis (^#^8), Catabolic Processes (^#^9) and Cellular Metabolic Processes (^#^10). The enriched GO/pathway terms are illustrated in corresponding network Fig. 3b.

**Additional file 6 **Results of GO/pathway term network analysis (ClueGO) associated with down-regulated differentially expressed genes (DEGs) of the Warm & Hypoxic (WH) treatment group. Listed are the significantly enriched Gene Ontology (GO) terms, and KEGG or Reactome pathways (hypergeometric test *p <* 0.05 with Benjamini-Hochberg correction). GO/pathway term annotations were obtained using the GO database for biological process, cellular component, molecular function, and immune processes, and the KEGG and Reactome pathway databases. The enriched GO/pathway terms are ordered according to ten functional themes: Heat Shock Response (^#^1), Cellular Stress (^#^2), Oxidative Stress (^#^3), Apoptosis (^#^4), Immune Response (^#^5), Protein Processing & Localization (^#^6), Transcription (^#^7), Proteolysis (^#^8), Catabolic Processes (^#^9) and Cellular Metabolic Processes (^#^10). The enriched GO/pathway terms are illustrated in corresponding network Fig. 4b.

**Additional file 7 **Pearson correlation between mean log_2_ fold change (FC) values of microarray probes and log_2_ fold change (FC) of 41 target genes measured with qPCR (Fluidigm Biomark™). The relationship between data obtained for the 41 target genes by both methods was estimated using Pearson’s product-moment correlation. The correlation coefficient R and the significance of correlation (*p <* 0.0001***) are presented in the figure.

**Additional file 8.** Comparison of fold change (FC) values for 41 target genes obtained by 44 K microarray (Agilent) and qPCR (Fluidigm Biomark™) approach.

**Additional file 9.** Bootstrap ratios from the first two components (CP-1 and CP-2) of the component maps based on 41 target genes and seven phenotypic traits for the Warm & Normoxic (WN) and Warm & Hypoxic (WH) treatment groups. Bootstrap ratios were performed to identify the variables that contributed significantly to the variance of a given component. Bold values indicate bootstrap ratios whose magnitude exceed +/− 2 and are considered as significant.

**Additional file 10.** Primers used for qPCR validation analyses. Listed are gene symbol, gene name, the sequence of forward and reverse primers, the amplicon size in base pairs, GenBank accession number used for primer design and the determined primer efficiencies. See Additional file 11 for further details about BLASTn hits and primer properties.

**Additional file 11.** Details about target genes and primers used for qPCR validation analyses. Listed are specific details about the qPCR genes and primers, BLASTn results of amplicon sequences, and primer properties for 41 target genes and two normalizer genes.

## Data Availability

The whole microarray dataset consisted of 17,072 detected probes (normalized log_2_ ratios) and is accessible on-line on the NCBI’s Gene Expression Omnibus database through the GEO Series accession number GSE146471 (https://www.ncbi.nlm.nih.gov/geo/query/acc.cgi?acc=GSE146471). The obtained threshold cycle (CT) values for samples from the current study (Fish-ID ^#^73 to ^#^96, sampling time point at 20 °C for 3-days) are accessible on-line at PANGAEA (10.1594/PANGAEA.913696). In the Methods section, and in Additional files 1, 10 and 11, the GenBank transcript accession numbers were included for gene identification purposes.

## Abbreviations

air sat: Air Saturation
BP: Biological Process
CF: Condition Factor
CT: Control
C_T_: Threshold Cycle
CP-1: First Component
CP-2: Second Component
CV: Coefficient of Variation
DO: Dissolved Oxygen
DEG: Differentially Expressed Gene
DEP: Differentially Expressed Probe
ECM: Extracellular Matrix
ER: Endoplasmic Reticulum
EST: Expressed Sequence Tag
ELISA: Enzyme-linked Immunosorbent Assay
FC: Fold Change
FDR: False Discovery Rate
GAPP: Genomic Applications Partnership Program
GEO: Gene Expression Omnibus
GO: Gene Ontology
GOI: Gene of Interest
HSI: Hepato-Somatic Index
HSP: Heat Shock Protein
IL: Interleukin
KEGG: Kyoto Encyclopedia of Genes and Genomes
LASCCR: Laboratory for Atlantic Salmon and Climate Change Research
LME: Linear Mixed-Effect Model
MF: Molecular Function
MICCSA: Mitigating the Impacts of Climate-Related Challenges on Salmon Aquaculture
MMPs: Matrix Metalloproteinases
NTC: Non-Template Control
PCA: Principal Component Analysis
PC: Principal Component
PC-1: First Principal Component
PC-2: Second Principal Component
PIT: Passive Integrated Transponder
PMT: Photomultiplier Tube
qPCR: Quantitative PCR
ROS: Reactive Oxygen Species
RQ: Relative Quantity
RT: Reverse Transcription
RVM: Relative Ventricular Mass
SAM: Significance Analysis of Microarrays
SD: Standard Deviation
SEM: Standard Error of Mean
SGR: Specific Growth Rate
SNP: Single Nucleotide Polymorphism
SSI: Spleen-Somatic Index
TAG: Triacylglycerol
UPR: Unfolded Protein Response
WH: Warm & Hypoxic
WN: Warm & Normoxic

## References

[1] Wade NM, Clark TD, Maynard BT, Atherton S, Wilkinson RJ, Smullen RP (2019). Effects of an unprecedented summer heatwave on the growth performance, flesh colour and plasma biochemistry of marine cage-farmed Atlantic salmon (*Salmo salar*). J Therm Biol.

[2] Motyka R, Norin T, Petersen LH, Huggett DB, Gamperl AK. Long-term hypoxia exposure alters the cardiorespiratory physiology of steelhead trout (*Oncorhynchus mykiss*), but does not affect their upper thermal tolerance. J Therm Biol 2017;68 March 2016:149–161. 10.1016/j.jtherbio.2016.03.007 28797475

[3] Vikeså V, Nankervis L, Hevrøy EM (2017). Appetite, metabolism and growth regulation in Atlantic salmon (*Salmo salar* L.) exposed to hypoxia at elevated seawater temperature. Aquac Res.

[4] McBryan TL, Anttila K, Healy TM, Schulte PM (2013). Responses to temperature and hypoxia as interacting stressors in fish: implications for adaptation to environmental change. Integr Comp Biol.

[5] Currie S, Schulte PM, Evans DH, Claiborne JB, Currie S (2014). Thermal stress. The physiology of fishes.

[6] Kvamme BO, Gadan K, Finne-Fridell F, Niklasson L, Sundh H, Sundell K (2013). Modulation of innate immune responses in Atlantic salmon by chronic hypoxia-induced stress. Fish Shellfish Immunol..

[7] Frölicher TL, Fischer EM, Gruber N (2018). Marine heatwaves under global warming. Nature..

[8] Oliver ECJ, Donat MG, Burrows MT, Moore PJ, Smale DA, Alexander LV (2018). Longer and more frequent marine heatwaves over the past century. Nat Commun.

[9] IPCC. Summary for policymakers. In: Pörtner H-O, Roberts DC, Masson-Delmotte V, Zhai P, Tignor M, Poloczanska E, Mintenbeck K, Alegría A, Nicolai M, Okem A, Petzold J, Rama B, Weyer NM, editors. IPCC special report on the ocean and cryosphere in a changing climate. 2019.

[10] Breitburg D, Levin LA, Oschlies A, Grégoire M, Chavez FP, Conley DJ (2018). Declining oxygen in the global ocean and coastal waters. Science (80).

[11] Claret M, Galbraith ED, Palter JB, Bianchi D, Fennel K, Gilbert D (2018). Rapid coastal deoxygenation due to ocean circulation shift in the Northwest Atlantic. Nat Clim Chang.

[12] Kültz D (2005). Molecular and evolutionary basis of the cellular stress response. Annu Rev Physiol.

[13] Jeffries KM, Hinch SG, Sierocinski T, Clark TD, Eliason EJ, Donaldson MR (2012). Consequences of high temperatures and premature mortality on the transcriptome and blood physiology of wild adult sockeye salmon (*Oncorhynchus nerka*). Ecol Evol.

[14] Olsvik PA, Vikesa V, Lie KK, Hevroy EM (2013). Transcriptional responses to temperature and low oxygen stress in Atlantic salmon studied with next-generation sequencing technology. BMC Genomics.

[15] Rebl A, Verleih M, Köbis JM, Kühn C, Wimmers K, Köllner B (2013). Transcriptome profiling of gill tissue in regionally bred and globally farmed rainbow trout strains reveals different strategies for coping with thermal stress. Mar Biotechnol.

[16] Shi K-P, Dong S-L, Zhou Y-G, Li Y, Gao Q-F, Sun D-J (2019). RNA-seq reveals temporal differences in the transcriptome response to acute heat stress in the Atlantic salmon (*Salmo salar*). Comp Biochem Physiol Part D Genomics Proteomics.

[17] Jeffries KM, Hinch SG, Sierocinski T, Pavlidis P, Miller KM (2014). Transcriptomic responses to high water temperature in two species of Pacific salmon. Evol Appl.

[18] Tomalty KMH, Meek MH, Stephens MR, Rincón G, Fangue NA, May BP (2015). Transcriptional response to acute thermal exposure in juvenile Chinook salmon determined by RNAseq. Genes, Genomes, Genet.

[19] Oku H, Tokuda M, Matsunari H, Furuita H, Murashita K, Yamamoto T (2014). Characterization of differentially expressed genes in liver in response to the rearing temperature of rainbow trout *Oncorhynchus mykiss* and their heritable differences. Fish Physiol Biochem.

[20] Li Y, Huang J, Liu Z, Zhou Y, Xia B, Wang Y (2017). Transcriptome analysis provides insights into hepatic responses to moderate heat stress in the rainbow trout (*Oncorhynchus mykiss*). Gene..

[21] Liu S, Wang X, Sun F, Zhang J, Feng J, Liu H (2013). RNA-Seq reveals expression signatures of genes involved in oxygen transport, protein synthesis, folding, and degradation in response to heat stress in catfish. Physiol Genomics.

[22] Jesus TF, Grosso AR, Almeida-Val VMF, Coelho MM (2016). Transcriptome profiling of two Iberian freshwater fish exposed to thermal stress. J Therm Biol.

[23] Guo L, Wang Y, Liang S, Lin G, Chen S, Yang G (2016). Tissue-overlapping response of half-smooth tongue sole (*Cynoglossus semilaevis*) to thermostressing based on transcriptome profiles. Gene..

[24] Richards JG. Chapter 10 Metabolic and molecular responses of fish to hypoxia. In: Fish physiology. Vol. 27. Richards JG, Farrell AP, Brauner CJ, editors. Academic Press; 2009. p. 443–485.

[25] Gracey AY, Troll JV, Somero GN (2001). Hypoxia-induced gene expression profiling in the euryoxic fish *Gillichthys mirabilis*. Proc Natl Acad Sci U S A.

[26] van der Meer DLM, van den Thillart GEEJM, Witte F, de Bakker MAG, Besser J, Richardson MK (2005). Gene expression profiling of the long-term adaptive response to hypoxia in the gills of adult zebrafish. Am J Physiol Integr Comp Physiol.

[27] Qi D, Chao Y, Wu R, Xia M, Chen Q, Zheng Z (2018). Transcriptome analysis provides insights into the adaptive responses to hypoxia of a schizothoracine fish (*Gymnocypris eckloni*). Front Physiol.

[28] Ton C, Stamatiou D, Liew C-C (2003). Gene expression profile of zebrafish exposed to hypoxia during development. Physiol Genomics.

[29] Wang Q-F, Shen W-L, Hou C-C, Liu C, Wu X-F, Zhu J-Q (2017). Physiological responses and changes in gene expression in the large yellow croaker *Larimichthys crocea* following exposure to hypoxia. Chemosphere..

[30] Chen B-X, Yi S-K, Wang W-F, He Y, Huang Y, Gao Z-X (2017). Transcriptome comparison reveals insights into muscle response to hypoxia in blunt snout bream (*Megalobrama amblycephala*). Gene..

[31] Gong D, Xu L, Li W, Shang R, Chen J, Hu F (2020). Comparative analysis of liver transcriptomes associated with hypoxia tolerance in the gynogenetic blunt snout bream. Aquaculture..

[32] Prado-Lima M, Val AL (2016). Transcriptomic characterization of tambaqui (*Colossoma macropomum*, Cuvier, 1818) exposed to three climate change scenarios. PLoS One.

[33] Burke H, Gardner I, Farrell AP. A review of the 2019 Newfoundland and Labrador South Coast cultured Atlantic salmon mortality event. Department of Fisheries and Land Resources, Government of Newfoundland and Labrador, Special Studies and Reports. 2020. Available online at: https://www.gov.nl.ca/ffa/files/publications-pdf-2019-salmon-review-final-report.pdf

[34] Burt K, Hamoutene D, Mabrouk G, Lang C, Puestow T, Drover D (2012). Environmental conditions and occurrence of hypoxia within production cages of Atlantic salmon on the south coast of Newfoundland. Aquac Res.

[35] Gunderson AR, Armstrong EJ, Stillman JH (2016). Multiple stressors in a changing world: the need for an improved perspective on physiological responses to the dynamic marine environment. Annu Rev Mar Sci.

[36] Stehfest KM, Carter CG, McAllister JD, Ross JD, Semmens JM (2017). Response of Atlantic salmon *Salmo salar* to temperature and dissolved oxygen extremes established using animal-borne environmental sensors. Sci Rep.

[37] Handeland SO, Imsland AK, Stefansson SO (2008). The effect of temperature and fish size on growth, feed intake, food conversion efficiency and stomach evacuation rate of Atlantic salmon post-smolts. Aquaculture..

[38] Hevrøy EM, Hunskår C, de Gelder S, Shimizu M, Waagbø R, Breck O (2013). GH-IGF system regulation of attenuated muscle growth and lipolysis in Atlantic salmon reared at elevated sea temperatures. J Comp Physiol B Biochem Syst Environ Physiol.

[39] Solstorm D, Oldham T, Solstorm F, Klebert P, Stien LH, Vågseth T (2018). Dissolved oxygen variability in a commercial sea-cage exposes farmed Atlantic salmon to growth limiting conditions. Aquaculture..

[40] Oldham T, Oppedal F, Dempster T (2018). Cage size affects dissolved oxygen distribution in salmon aquaculture. Aquac Environ Interact.

[41] Oppedal F, Vågseth T, Dempster T, Juell J-E, Johansson D (2011). Fluctuating Sea-cage environments modify the effects of stocking densities on production and welfare parameters of Atlantic salmon (*Salmo salar* L.). Aquaculture..

[42] Gamperl AK, Ajiboye OO, Zanuzzo FS, Sandrelli RM, Peroni EFC, Beemelmanns A (2020). The impacts of increasing temperature and moderate hypoxia on the production characteristics, cardiac morphology and haematology of Atlantic salmon (*Salmo salar*). Aquaculture..

[43] Tierney KB, Farrell AP, Brauner CJ. Organic chemical toxicology of fishes. In: Fish physiology. Vol. 33. Richards JG, Farrell AP, Brauner CJ, editors. Academic Press; 2013. p. 453–455.

[44] Jantzen SG, Sanderson DS, von Schalburg KR, Yasuike M, Marass F, Koop BF (2011). A 44K microarray dataset of the changing transcriptome in developing Atlantic salmon (*Salmo salar* L.). BMC Res Notes.

[45] Bindea G, Mlecnik B, Hackl H, Charoentong P, Tosolini M, Kirilovsky A (2009). ClueGO: a Cytoscape plug-in to decipher functionally grouped gene ontology and pathway annotation networks. Bioinformatics..

[46] Reid G, Gurney-Smith H, Marcogliese D, Knowler D, Benfey T, Garber A (2019). Climate change and aquaculture: considering biological response and resources. Aquac Environ Interact..

[47] Mohanty BP, Mahanty A, Mitra T, Parija SC, Mohanty S. Regulation of heat shock protein responses. In: Asea A, Kaur P, editors. Regulation of heat shock protein responses. Vol. 13. Springer International Publishing; 2018. p. 71–94.

[48] Hartl FU, Hayer-Hartl M (2002). Protein folding. Molecular chaperones in the cytosol: from nascent chain to folded protein. Science (80).

[49] Parsell DA, Lindquist S (1993). The function of heat-shock proteins in stress tolerance: degradation and reactivation of damaged proteins. Annu Rev Genet.

[50] Srivastava P (2002). Interaction of heat shock proteins with peptides and antigen presenting cells: chaperoning of the innate and adaptive immune responses. Annu Rev Immunol.

[51] Callahan MK, Garg M, Srivastava PK (2008). Heat-shock protein 90 associates with N-terminal extended peptides and is required for direct and indirect antigen presentation. Proc Natl Acad Sci U S A.

[52] Lanneau D, Brunet M, Frisan E, Solary E, Fontenay M, Garrido C (2008). Heat shock proteins: essential proteins for apoptosis regulation. J Cell Mol Med.

[53] Ghosh S, Sarkar P, Basak P, Mahalanobish S, Sil PC, Asea A, Kaur P (2018). Role of heat shock proteins in oxidative stress and stress tolerance. Heat shock proteins and stress.

[54] Roberts RJ, Agius C, Saliba C, Bossier P, Sung YY (2010). Heat shock proteins (chaperones) in fish and shellfish and their potential role in relation to fish health: a review. J Fish Dis.

[55] Akbarzadeh A, Günther OP, Houde AL, Li S, Ming TJ, Jeffries KM (2018). Developing specific molecular biomarkers for thermal stress in salmonids. BMC Genomics.

[56] Ishida Y, Nagata K. Hsp47 as a collagen-specific molecular chaperon. In: Whisstock JC, Bird PI, editors. Methods in enzymology. Vol. 499. Academic Press; 2011. p. 167–182. 10.1016/B978-0-12-386471-0.00009-2 21683254

[57] Wang Y, Liu Z, Li Z, Shi H, Kang Y, Wang J (2016). Effects of heat stress on respiratory burst, oxidative damage and SERPINH1 (HSP47) mRNA expression in rainbow trout *Oncorhynchus mykiss*. Fish Physiol Biochem.

[58] Swirplies F, Wuertz S, Baßmann B, Orban A, Schäfer N, Brunner RM (2019). Identification of molecular stress indicators in pikeperch *Sander lucioperca* correlating with rising water temperatures. Aquaculture..

[59] Jackson SE. Hsp90: structure and function. In: Jackson S, editor. Molecular chaperones. Topics in current chemistry. Vol. 328. Springer Berlin Heidelberg; 2013. p. 155–240. 10.1007/128_2012_356 22955504

[60] Buchner J, Jing L (2013). Structure, function and regulation of the hsp90 machinery. Biom J.

[61] Kiang JG, Tsokos GC (1998). Heat shock protein 70 kDa: molecular biology, biochemistry, and physiology. Pharmacol Ther.

[62] Sitia R, Braakman I (2003). Quality control in the endoplasmic reticulum protein factory. Nature..

[63] D’Arcy MS (2019). Cell death: a review of the major forms of apoptosis, necrosis and autophagy. Cell Biol Int.

[64] Cheng CH, Yang FF, Liao SA, Miao YT, Ye CX, Wang AL (2015). High temperature induces apoptosis and oxidative stress in pufferfish (*Takifugu obscurus*) blood cells. J Therm Biol.

[65] Li B, Sun S, Zhu J, Yanli S, Wuxiao Z, Ge X (2019). Transcriptome profiling and histology changes in juvenile blunt snout bream (*Megalobrama amblycephala*) liver tissue in response to acute thermal stress. Genomics..

[66] Poon WL, Hung CY, Nakano K, Randall DJ (2007). An in vivo study of common carp (*Cyprinus carpio* L.) liver during prolonged hypoxia. Comp Biochem Physiol - Part D Genomics Proteomics..

[67] Liu B, Xu P, Brown PB, Xie J, Ge X, Miao L (2016). The effect of hyperthermia on liver histology, oxidative stress and disease resistance of the Wuchang bream, *Megalobrama amblycephala*. Fish Shellfish Immunol.

[68] Kruidering M, Evan G (2000). Caspase-8 in apoptosis: the beginning of “the end”?. IUBMB Life.

[69] Weitzman JB, Fiette L, Matsuo K, Yaniv M (2000). JunD protects cells from p53-dependent senescence and apoptosis. Mol Cell.

[70] Hori TS, Gamperl a K, Nash G, Booman M, Barat A, Rise ML. The impact of a moderate chronic temperature increase on spleen immune-relevant gene transcription depends on whether Atlantic cod (*Gadus morhua*) are stimulated with bacterial versus viral antigens. Genome. 2013;56:567–76. 10.1139/gen-2013-0090 24237337

[71] Crespo-Sanjuán J, Zamora-Gonzalez N, Calvo-Nieves MD, Andres-Ledesma C (2017). Apolipoprotein D.

[72] Holland MCH, Lambris JD (2002). The complement system in teleosts. Fish Shellfish Immunol..

[73] Nikoskelainen S, Kjellsen O, Lilius EM, Schrøder MB (2006). Respiratory burst activity of Atlantic cod (*Gadus morhua* L.) blood phagocytes differs markedly from that of rainbow trout. Fish Shellfish Immunol.

[74] Zanuzzo FS, Beemelmanns A, Hall JR, Rise ML, Gamperl AK (2020). The innate immune response of Atlantic salmon (*Salmo salar*) is not negatively affected by high temperature and moderate hypoxia. Front Immunol.

[75] Nayak A, Pednekar L, Reid KB, Kishore U (2012). Complement and non-complement activating functions of C1q: a prototypical innate immune molecule. Innate Immun.

[76] Bowden TJ, Thompson KD, Morgan AL, Gratacap RML, Nikoskelainen S (2007). Seasonal variation and the immune response: a fish perspective. Fish Shellfish Immunol..

[77] Kunisawa J, Shastri N (2006). Hsp90α chaperones large C-terminally extended proteolytic intermediates in the MHC class I antigen processing pathway. Immunity..

[78] Rajagopal D, Bal V, Mayor S, George A, Rath S (2006). A role for the Hsp90 molecular chaperone family in antigen presentation to T lymphocytes via major histocompatibility complex class II molecules. Eur J Immunol.

[79] Yabluchanskiy A, Ma Y, Iyer RP, Hall ME, Lindsey ML (2013). Matrix metalloproteinase-9: many shades of function in cardiovascular disease. Physiology..

[80] Caley MP, Martins VLC, O’Toole EA (2015). Metalloproteinases and wound healing. Adv Wound Care.

[81] Kimes NE, Grim CJ, Johnson WR, Hasan NA, Tall BD, Kothary MH (2012). Temperature regulation of virulence factors in the pathogen *Vibrio coralliilyticus*. ISME J.

[82] Vezzulli L, Previati M, Pruzzo C, Marchese A, Bourne DG, Cerrano C (2010). *Vibrio* infections triggering mass mortality events in a warming Mediterranean Sea. Environ Microbiol.

[83] Zanuzzo FS, Sabioni RE, Marzocchi-Machado CM, Urbinati EC (2019). Modulation of stress and innate immune response by corticosteroids in pacu (*Piaractus mesopotamicus*). Comp Biochem Physiol Part A Mol Integr Physiol.

[84] Pérez-Casanova JC, Rise ML, Dixon B, Afonso LOB, Hall JR, Johnson SC (2008). The immune and stress responses of Atlantic cod to long-term increases in water temperature. Fish Shellfish Immunol..

[85] Banh S, Wiens L, Sotiri E, Treberg JR (2016). Mitochondrial reactive oxygen species production by fish muscle mitochondria: potential role in acute heat-induced oxidative stress. Comp Biochem Physiol Part B Biochem Mol Biol..

[86] Heise K (2006). Oxidative stress during stressful heat exposure and recovery in the North Sea eelpout *Zoarces viviparus* L. J Exp Biol.

[87] Belhadj Slimen I, Najar T, Ghram A, Dabbebi H, Ben Mrad M, Abdrabbah M (2014). Reactive oxygen species, heat stress and oxidative-induced mitochondrial damage. A review Int J Hyperth.

[88] Machado C, Zaleski T, Rodrigues E, Carvalho C dos S, Cadena SMSC, Gozzi GJ, et al. Effect of temperature acclimation on the liver antioxidant defence system of the Antarctic nototheniids *Notothenia coriiceps* and *Notothenia rossii*. Comp Biochem Physiol Part - B Biochem Mol Biol 2014;172–173:21–28. 10.1016/j.cbpb.2014.02.003 24607634

[89] Wenger RH (2002). Cellular adaptation to hypoxia: O_2_-sensing protein hydroxylases, hypoxia-inducible transcription factors, and O_2_-regulated gene expression. FASEB J.

[90] Kilic M, Kasperczyk H, Fulda S, Debatin K-M (2007). Role of hypoxia inducible factor-1 alpha in modulation of apoptosis resistance. Oncogene..

[91] Rimoldi S, Terova G, Ceccuzzi P, Marelli S, Antonini M, Saroglia M (2012). HIF-1α mRNA levels in Eurasian perch (*Perca fluviatilis*) exposed to acute and chronic hypoxia. Mol Biol Rep.

[92] Rahman MS, Thomas P (2017). Molecular and biochemical responses of hypoxia exposure in Atlantic croaker collected from hypoxic regions in the northern Gulf of Mexico. PLoS One.

[93] Terova G, Rimoldi S, Corà S, Bernardini G, Gornati R, Saroglia M (2008). Acute and chronic hypoxia affects HIF-1α mRNA levels in sea bass (*Dicentrarchus labrax*). Aquaculture..

[94] Semenza GL (2001). HIF-1, O_2_, and the 3 PHDs. Cell..

[95] Del Peso L, Castellanos MC, Temes E, Martín-Puig S, Cuevas Y, Olmos G (2003). The von Hippel Lindau/hypoxia-inducible factor (HIF) pathway regulates the transcription of the HIF-proline hydroxylase genes in response to low oxygen. J Biol Chem.

[96] Taylor MS (2001). Characterization and comparative analysis of the EGLN gene family. Gene..

[97] Liu W, Liu X, Wu C, Jiang L (2018). Transcriptome analysis demonstrates that long noncoding RNA is involved in the hypoxic response in *Larimichthys crocea*. Fish Physiol Biochem.

[98] Yuan G, Nanduri J, Bhasker CR, Semenza GL, Prabhakar NR (2005). Ca^2+^/calmodulin kinase-dependent activation of hypoxia inducible factor 1 transcriptional activity in cells subjected to intermittent hypoxia. J Biol Chem.

[99] Bistolas N, Wollenberger U, Jung C, Scheller FW (2005). Cytochrome P450 biosensors—a review. Biosens Bioelectron.

[100] Olsvik PA (2006). Effects of hypo- and hyperoxia on transcription levels of five stress genes and the glutathione system in liver of Atlantic cod *Gadus morhua*. J Exp Biol.

[101] Rahman MS, Thomas P (2012). Effects of hypoxia exposure on hepatic cytochrome P450 1A (CYP1A) expression in Atlantic croaker: molecular mechanisms of CYP1A down-regulation. PLoS One.

[102] Brand MD, Esteves TC (2005). Physiological functions of the mitochondrial uncoupling proteins UCP2 and UCP3. Cell Metab.

[103] Bermejo-Nogales A, Calduch-Giner JA, Pérez-Sánchez J (2014). Tissue-specific gene expression and functional regulation of uncoupling protein 2 (UCP2) by hypoxia and nutrient availability in gilthead sea bream (*Sparus aurata*): implications on the physiological significance of UCP1–3 variants. Fish Physiol Biochem.

[104] Laskowski M, Augustynek B, Kulawiak B, Koprowski P, Bednarczyk P, Jarmuszkiewicz W (1857). What do we not know about mitochondrial potassium channels?. Biochim Biophys Acta Bioenerg.

[105] Gerber L, Clow KA, Mark FC, Gamperl AK (2020). Improved mitochondrial function in salmon (*Salmo salar*) following high temperature acclimation suggests that there are cracks in the proverbial ‘ceiling’. Sci Rep.

[106] Arevalo J, Vázquez-Medina J (2018). The role of peroxiredoxin 6 in cell signaling. Antioxidants..

[107] Park H, Ahn I-Y, Kim H, Cheon J, Kim M (2008). Analysis of ESTs and expression of two peroxiredoxins in the thermally stressed Antarctic bivalve *Laternula elliptica*. Fish Shellfish Immunol..

[108] Tolomeo AM, Carraro A, Bakiu R, Toppo S, Place SP, Ferro D (2016). Peroxiredoxin 6 from the Antarctic emerald rockcod: molecular characterization of its response to warming. J Comp Physiol B.

[109] Zhong P, Huang H (2017). Recent progress in the research of cold-inducible RNA-binding protein. Futur Sci OA.

[110] Kullgren A, Jutfelt F, Fontanillas R, Sundell K, Samuelsson L, Wiklander K (2013). The impact of temperature on the metabolome and endocrine metabolic signals in Atlantic salmon (*Salmo salar*). Comp Biochem Physiol Part A Mol Integr Physiol..

[111] Chen X, Liu X, Li B, Zhang Q, Wang J, Zhang W (2017). Cold inducible RNA binding protein is involved in chronic hypoxia induced neuron apoptosis by down-regulating HIF-1α expression and regulated by microRNA-23a. Int J Biol Sci.

[112] Fé-Gonçalves LM, Araújo JDA, dos Santos CH dos A, Val AL, Almeida-Val VMF. How will farmed populations of freshwater fish deal with the extreme climate scenario in 2100? Transcriptional responses of *Colossoma macropomum* from two Brazilian climate regions J Therm Biol 2020;89:102487. 10.1016/j.jtherbio.2019.102487 32364997

[113] Zhong X-P, Wang D, Zhang Y-B, Gui J-F (2009). Identification and characterization of hypoxia-induced genes in *Carassius auratus* blastulae embryonic cells using suppression subtractive hybridization. Comp Biochem Physiol Part B Biochem Mol Biol.

[114] Li M, Wang X, Qi C, Li E, Du Z, Qin JG (2018). Metabolic response of Nile tilapia (*Oreochromis niloticus*) to acute and chronic hypoxia stress. Aquaculture..

[115] Kuntz MJ, Harris RA, Choi S (2018). Pyruvate dehydrogenase kinase. Encyclopedia of signaling molecules.

[116] Edwards JR, Yarychkivska O, Boulard M, Bestor TH (2017). DNA methylation and DNA methyltransferases. Epigenetics Chromatin.

[117] Beemelmanns A, Ribas L, Anastasiadi D, Moraleda-Prados J, Zanuzzo FS, Rise ML, Gamperl AK. DNA methylation dynamics in Atlantic salmon (*Salmo salar*) challenged with high temperature and moderate hypoxia. Front Mar Sci. 2021;7:604878.

[118] Eissa N, Wang H-P (2016). Transcriptional stress responses to environmental and husbandry stressors in aquaculture species. Rev Aquac.

[119] Houde ALS, Akbarzadeh A, Günther OP, Li S, Patterson DA, Farrell AP (2019). Salmonid gene expression biomarkers indicative of physiological responses to changes in salinity and temperature, but not dissolved oxygen. J Exp Biol.

[120] Webb D, Gagnon MM (2009). The value of stress protein 70 as an environmental biomarker of fish health under field conditions. Environ Toxicol.

[121] Kilkenny C, Browne WJ, Cuthill IC, Emerson M, Altman DG (2010). Improving bioscience research reporting: the ARRIVE guidelines for reporting animal research. PLoS Biol.

[122] Champely S, Ekstrom C, Dalgaard P, Gill J, Weibelzahl S, Anandkumar A, et al. Package ‘pwr.’ R Packag version 2018;1.

[123] Xu Q, Feng CY, Hori TS, Plouffe DA, Buchanan JT, Rise ML (2013). Family-specific differences in growth rate and hepatic gene expression in juvenile triploid growth hormone (GH) transgenic Atlantic salmon (*Salmo salar*). Comp Biochem Physiol Part D Genomics Proteomics..

[124] Umasuthan N, Xue X, Caballero-Solares A, Kumar S, Westcott JD, Chen Z (2020). Transcriptomic profiling in fins of Atlantic salmon parasitized with sea lice: evidence for an early imbalance between chalimus-induced immunomodulation and the host’s defense response. Int J Mol Sci.

[125] Caballero-Solares A, Hall JR, Xue X, Eslamloo K, Taylor RG, Parrish CC (2017). The dietary replacement of marine ingredients by terrestrial animal and plant alternatives modulates the antiviral immune response of Atlantic salmon (*Salmo salar*). Fish Shellfish Immunol..

[126] Xue X, Hixson SM, Hori TS, Booman M, Parrish CC, Anderson DM (2015). Atlantic salmon (*Salmo salar*) liver transcriptome response to diets containing *Camelina sativa* products. Comp Biochem Physiol Part D Genomics Proteomics..

[127] Qiu W, Lee M-LT, Whitmore GA. Sample Size and Power Calculation in Microarray Studies Using the *sizepower* Package. R Packag version 132. 2008;10.

[128] Brazma A, Hingamp P, Quackenbush J, Sherlock G, Spellman P, Stoeckert C (2001). Minimum information about a microarray experiment (MIAME)-toward standards for microarray data. Nat Genet.

[129] Yang YH, Paquet AC. Preprocessing two-color spotted arrays. In: Gentleman R, Carey VJ, Huber W, Irizarry RA, Dudoit S, editors. Bioinformatics and computational biology solutions using R and bioconductor. Springer; 2005. p. 49–69.

[130] Booman M, Xu Q, Rise ML (2014). Evaluation of the impact of camelina oil-containing diets on the expression of genes involved in the innate anti-viral immune response in Atlantic cod (*Gadus morhua*). Fish Shellfish Immunol..

[131] Bø TH, Dysvik B, Jonassen I (2004). LSimpute: accurate estimation of missing values in microarray data with least squares methods. Nucleic Acids Res.

[132] Tusher VG, Tibshirani R, Chu G (2001). Significance analysis of microarrays applied to the ionizing radiation response. Proc Natl Acad Sci U S A.

[133] Schwender H, Krause A, Ickstadt K (2006). Identifying interesting genes with siggenes. Newsl R Proj.

[134] Kolde R, Kolde MR (2015). Package ‘pheatmap’. R Packag.

[135] Benjamini Y, Hochberg Y (1995). Controlling the false discovery rate: a practical and powerful approach to multiple testing. J R Stat Soc Ser B.

[136] Ashburner M, Ball CA, Blake JA, Botstein D, Butler H, Cherry JM (2000). Gene ontology: tool for the unification of biology. Nat Genet.

[137] Kanehisa M, Goto S, Kawashima S, Nakaya A (2002). The KEGG databases at GenomeNet. Nucleic Acids Res.

[138] Joshi-Tope G, Gillespie M, Vastrik I, D’Eustachio P, Schmidt E, de Bono B (2005). Reactome: A knowledgebase of biological pathways. Nucleic Acids Res.

[139] Supek F, Bošnjak M, Škunca N, Šmuc T (2011). REVIGO summarizes and visualizes long lists of gene ontology terms. PLoS One.

[140] Caballero-Solares A, Xue X, Parrish CC, Foroutani MB, Taylor RG, Rise ML (2018). Changes in the liver transcriptome of farmed Atlantic salmon (*Salmo salar*) fed experimental diets based on terrestrial alternatives to fish meal and fish oil. BMC Genomics.

[141] Soto-Dávila M, Valderrama K, Inkpen SM, Hall JR, Rise ML, Santander J (2020). Effects of vitamin D2 (Ergocalciferol) and D3 (Cholecalciferol) on Atlantic salmon (*Salmo salar*) primary macrophage immune response to *Aeromonas salmonicida subsp. salmonicida* infection. Front Immunol.

[142] Eslamloo K, Xue X, Hall JR, Smith NC, Caballero-Solares A, Parrish CC (2017). Transcriptome profiling of antiviral immune and dietary fatty acid dependent responses of Atlantic salmon macrophage-like cells. BMC Genomics.

[143] Hellemans J, Mortier G, De Paepe A, Speleman F, Vandesompele J (2007). qBase relative quantification framework and software for management and automated analysis of real-time quantitative PCR data. Genome Biol.

[144] Beemelmanns A, Roth O (2016). Biparental immune priming in the pipefish *Syngnathus typhle*. Zoology..

[145] Bookout AL, Mangelsdorf DJ. Quantitative real-time PCR protocol for analysis of nuclear receptor signaling pathways. Nucl Recept Signal. 2003;1:nrs.01012. 10.1621/nrs.01012 PMC1402222 16604184

[146] R Core Team. R: A Language And Environment For Statistical Computing. 2018. Available online at: http://www.r-project.org/ (Accessed January, 2019).

[147] Dray S, Dufour AB, Thioulouse J. ade4: analysis of ecological data: exploratory and Euclidean methods in environmental sciences. R package version 1.7–2. 2015.

[148] Nguyen LH, Holmes S (2019). Ten quick tips for effective dimensionality reduction. PLoS Comput Biol.

[149] Pinheiro J, Bates D, DebRoy S, Sarkar D (2011). Linear and nonlinear mixed effects models. R Packag version..

[150] Lenth RV (2016). Least-squares means: the R package *lsmeans*. J Stat Sofw.

[151] Beaton D, Chin Fatt CR, Abdi H (2014). An ExPosition of multivariate analysis with the singular value decomposition in R. Comput Stat Data Anal.

[152] Friendly M (2002). Corrgrams: exploratory displays for correlatigon matrices. Am Stat.

