# Supplementary figures and images for: The transcriptomic responses of Atlantic salmon (Salmo salar) to high temperature stress alone, and in combination with moderate hypoxia
Source: BMC Genomics. 2021 Apr 12;22:261. doi: 10.1186/s12864-021-07464-x (PMC8042886; doi:10.1186/s12864-021-07464-x)

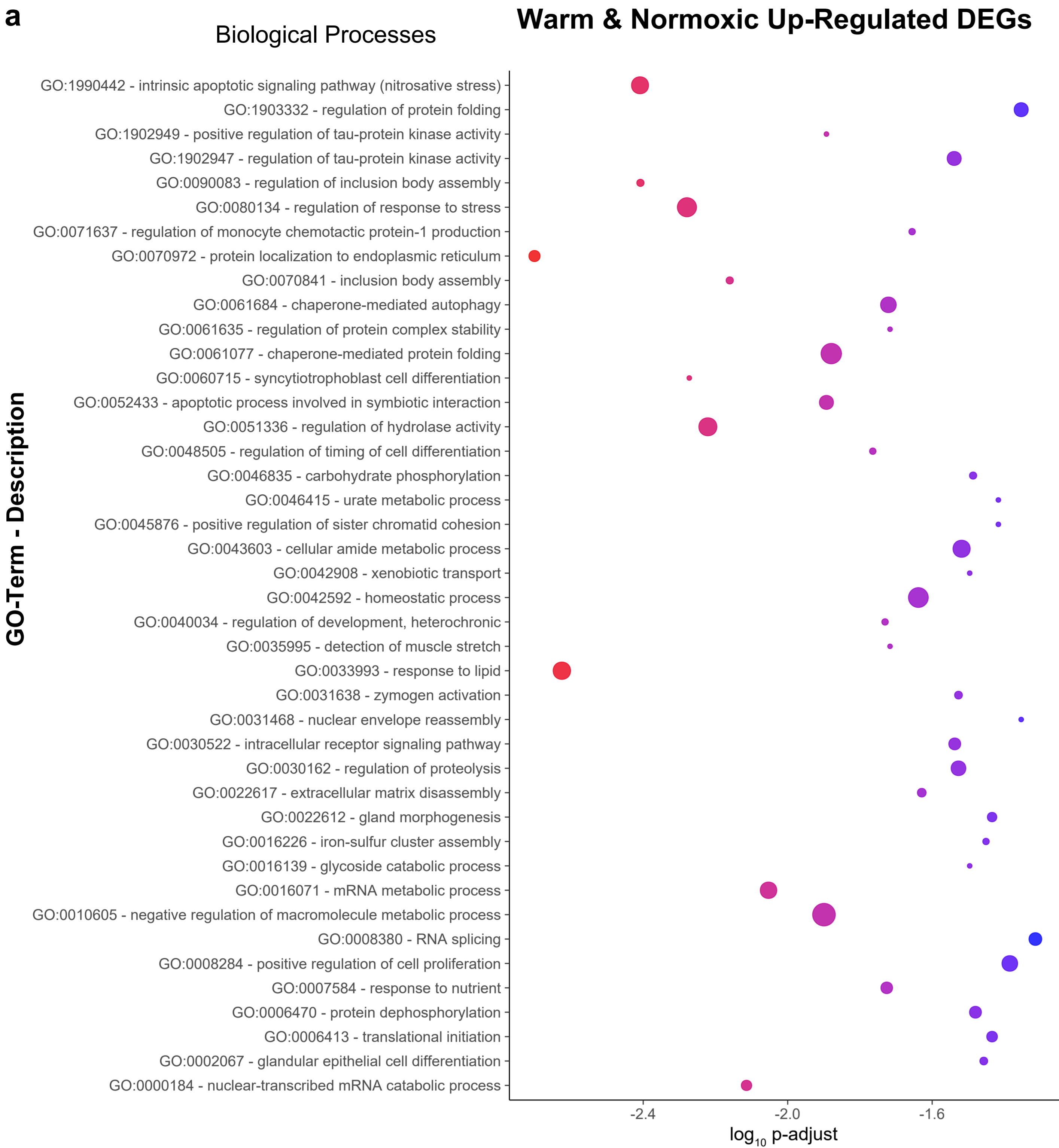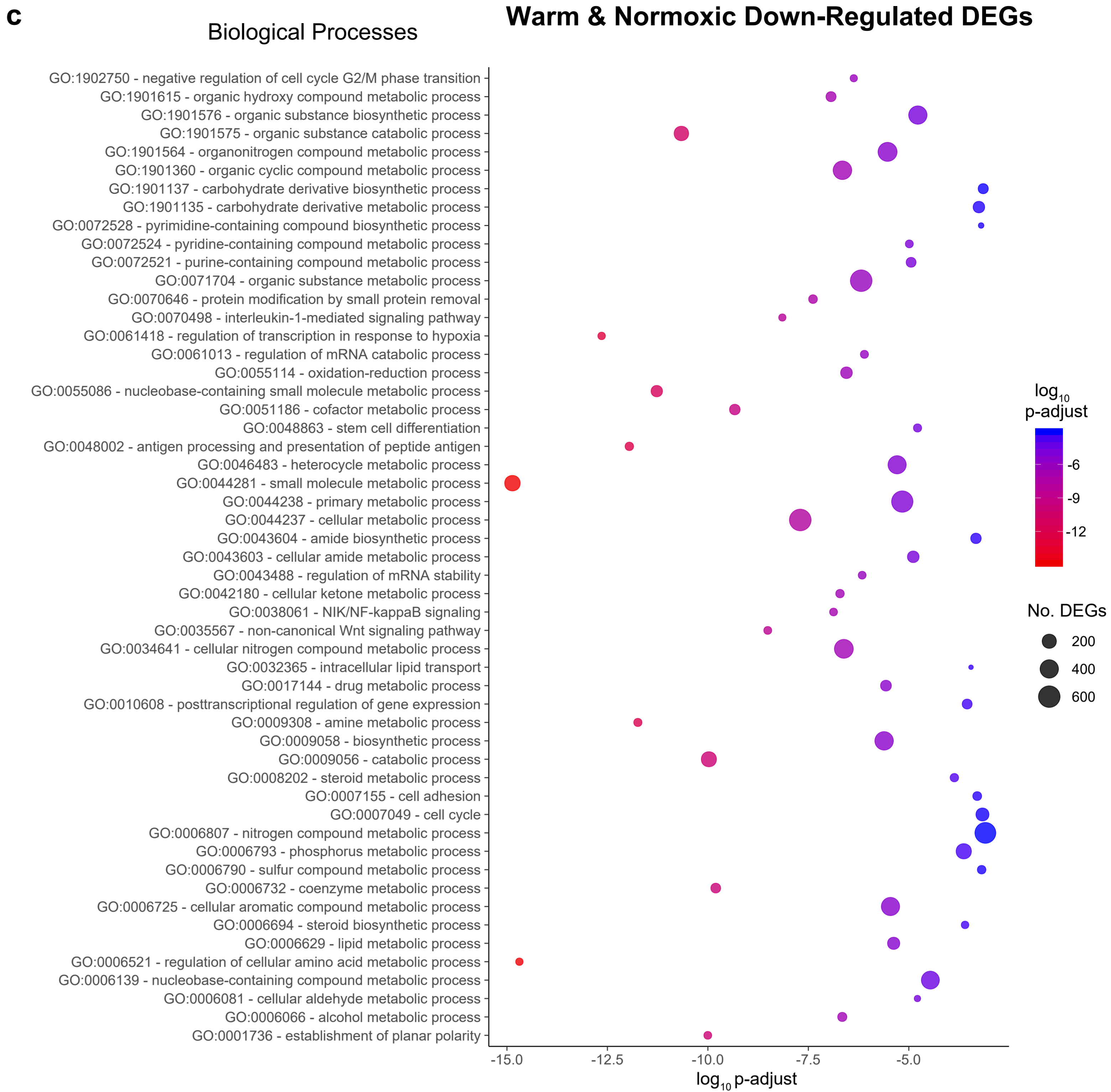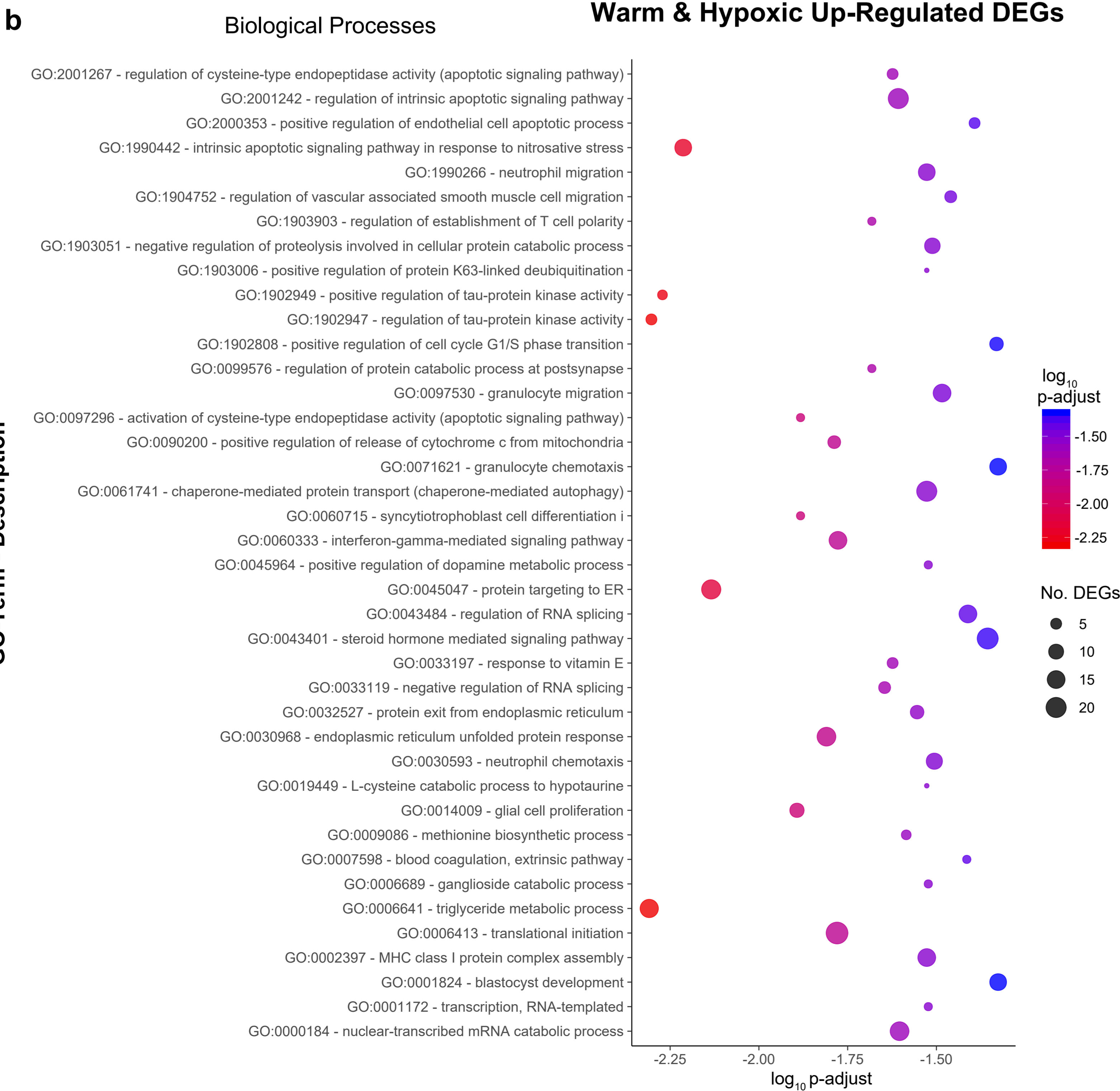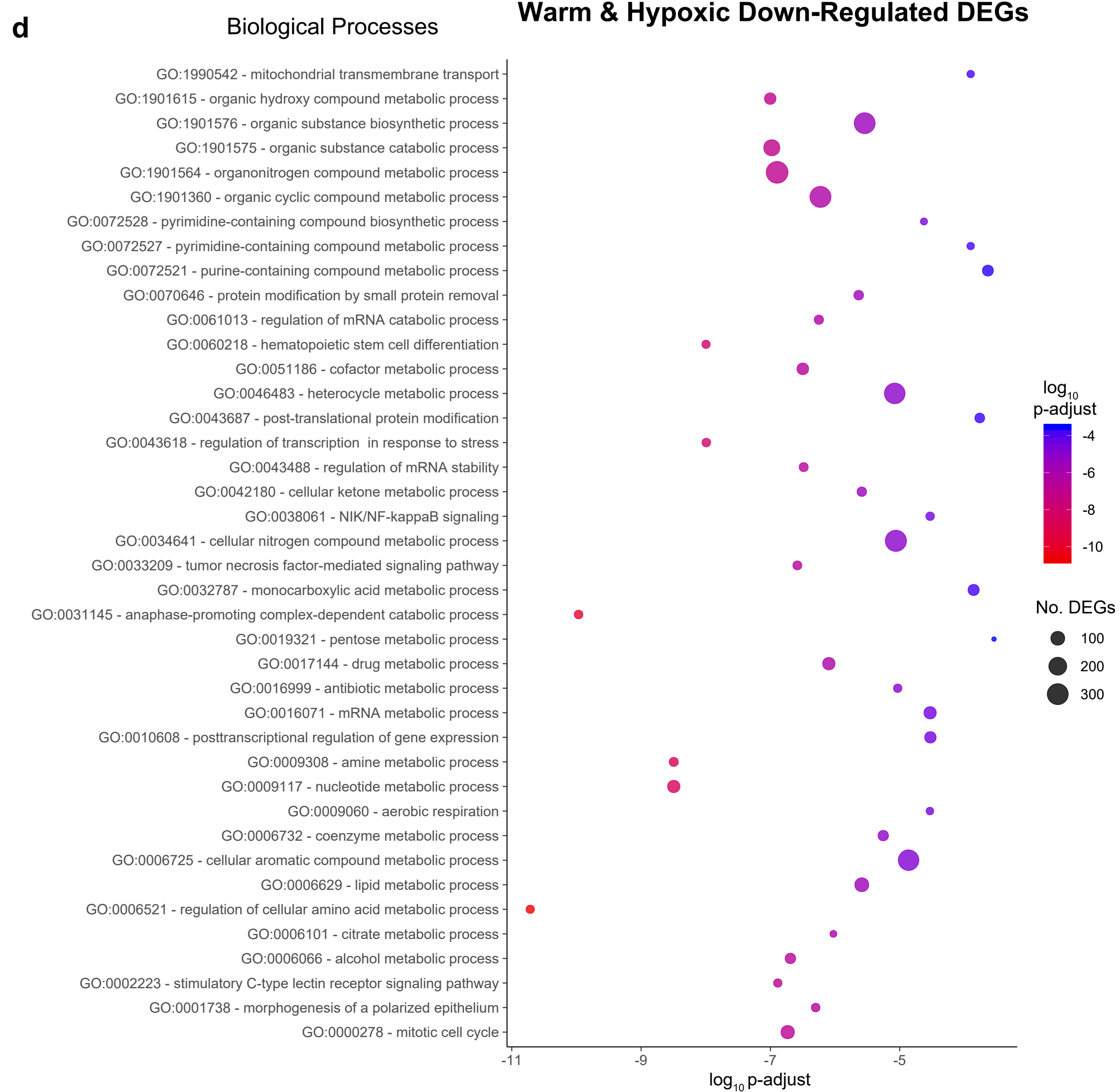

Supplement: Supplementary file 2 — Additional file 2 Enrichment GO-term dot plot for up- and down-regulated differentially expressed genes (DEGs) in Atlantic salmon that were subjected to Warm & Normoxic (WN: 20 °C, 100% air sat.) or Warm & Hypoxic (WH: 20 °C, ~ 70% air sat.) conditions. The dot plots represent non-redundant significantly enriched Gene Ontology (GO) terms of biological processes after application of REVIGO’s redundancy elimination algorithm for (a) up-regulated DEGs of the Warm & Normoxic group; (b) up-regulated DEGs of the Warm & Hypoxic group; (c) down-regulated DEGs of the Warm & Normoxic group; and (d) down-regulated DEGs of the Warm & Hypoxic group. The colour scheme corresponds to the log10 adjusted p-values (Benjamini and Hochberg method), and the diameter of the dots represents the number of DEGs that were identified to be significantly associated with this specific term. [file 12864_2021_7464_MOESM2_ESM.pdf]

# Validation: Microarray vs qPCR-Fluidigm (41 Genes)

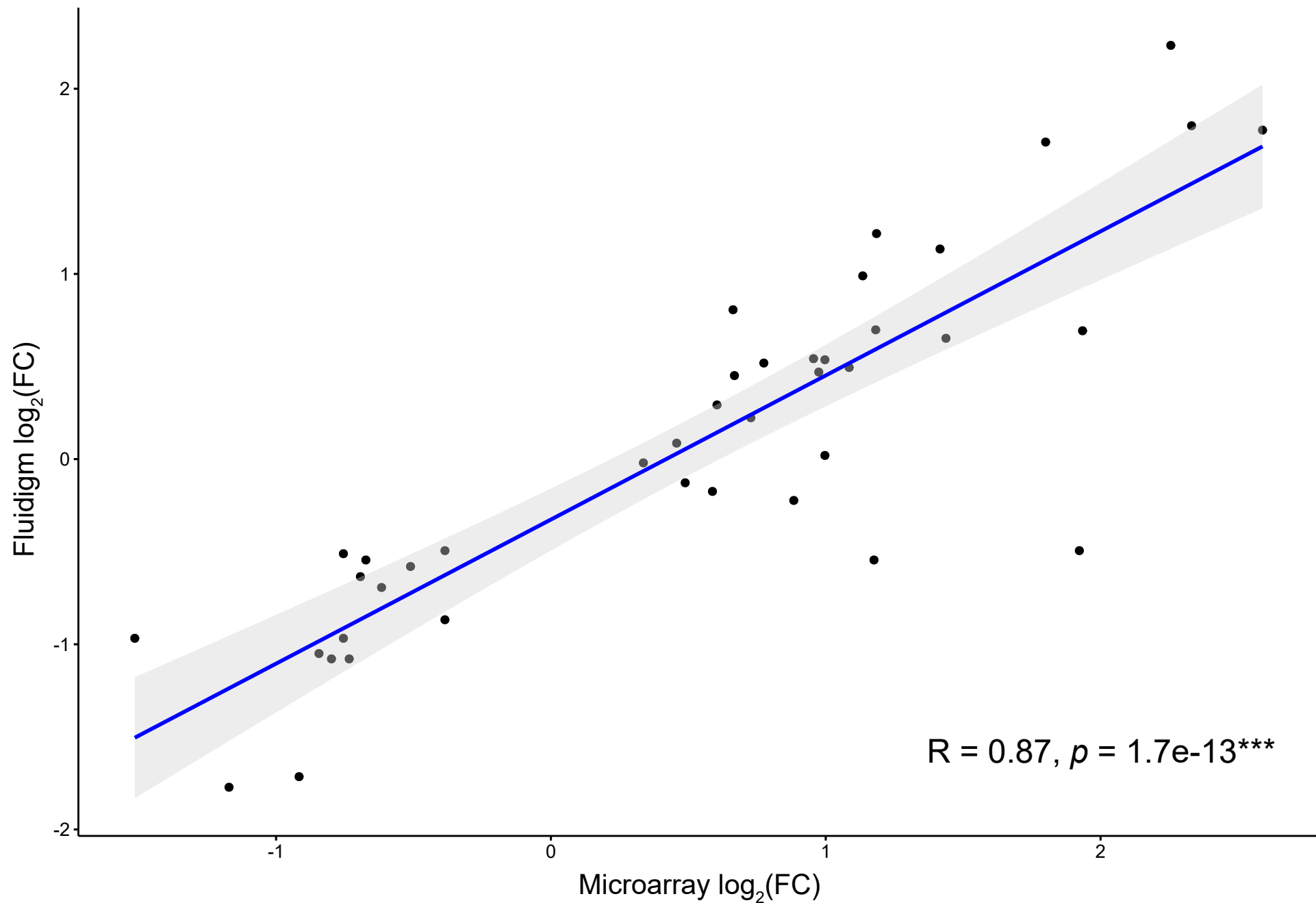

Supplement: Supplementary file 7 — Additional file 7 Pearson correlation between mean log2 fold change (FC) values of microarray probes and log2 fold change (FC) of 41 target genes measured with qPCR (Fluidigm Biomark™). The relationship between data obtained for the 41 target genes by both methods was estimated using Pearson’s product-moment correlation. The correlation coefficient R and the significance of correlation (p < 0.0001***) are presented in the figure. [file 12864_2021_7464_MOESM7_ESM.pdf]
