# Supplementary material for: The transcriptomic responses of Atlantic salmon (Salmo salar) to high temperature stress alone, and in combination with moderate hypoxia
Source: BMC Genomics. 2021 Apr 12;22:261. doi: 10.1186/s12864-021-07464-x (PMC8042886; doi:10.1186/s12864-021-07464-x)
Supplement: Supplementary file 8 — Additional file 8. Comparison of fold change (FC) values for 41 target genes obtained by 44 K microarray (Agilent) and qPCR (Fluidigm Biomark™) approach. [file 12864_2021_7464_MOESM8_ESM.docx]

**Additional file 8:** **Comparison of fold change (FC) values for 41 target genes obtained by 44K microarray (Agilent) and qPCR (Fluidigm Biomark^TM^) approach.**

|  |  | **Microarray (FC)** | | | **qPCR (FC)** | | | **Microarray vs qPCR** | | | |
| --- | --- | --- | --- | --- | --- | --- | --- | --- | --- | --- | --- |
| **Probe ID^1^** | **Gene Symbol** | **WN^2^** | **WH^3^** | **Direction^4^** | **WN^2^** | **WH^3^** | **Direction^4^** | **Mean FC Microarray^5^** | **Mean FC qPCR^5^** | **Direction^4^** | **Delta^6^** |
| C038R071 | ***apod*** | 6.32 | 14.27 | UP | 4.37 | 7.73 | UP | 10.29 | 6.05 | UP | 4.2 |
| C071R068 | ***c1ql2*** | 7.82 | 11.26 | UP | 6.39 | 12.29 | UP | 9.54 | 9.34 | UP | 0.2 |
| C097R144 | ***c3*** | 0.58 | 0.79 | DN | 0.60 | 0.62 | DN | 0.68 | 0.61 | DN | 0.1 |
| C219R132 | ***calm*** | 0.60 | 0.75 | DN | 0.42 | 0.42 | DN | 0.68 | 0.42 | DN | 0.3 |
| C234R046 | ***camp-a*** | 2.00 | 6.42 | UP | 0.87 | 2.97 | DN-UP | 4.21 | 1.92 | UP | 2.3 |
| C214R054 | ***casp8*** | 2.96 | 2.96 | UP | 1.71 | 1.57 | UP | 2.96 | 1.64 | UP | 1.3 |
| C004R029 | ***cat*** | 1.42 | 1.38 | UP | 1.01 | 0.95 | UP-DN | 1.40 | 0.98 | DIFF | 0.4 |
| C107R130 | ***cirbp*** | 0.47 | 0.39 | DN | 0.37 | 0.34 | DN | 0.43 | 0.35 | DN | 0.1 |
| C031R082 | ***cldn3*** | 1.29 | 1.98 | UP | 0.75 | 1.01 | DN-UP | 1.63 | 0.88 | DIFF | 0.8 |
| C194R156 | ***ctsh*** | 0.46 | 0.49 | DN | 0.33 | 0.35 | DN | 0.48 | 0.34 | DN | 0.1 |
| C043R081 | ***cul3*** | 1.68 | 1.48 | UP | 1.17 | 1.05 | UP | 1.58 | 1.09 | UP | 0.5 |
| C161R028 | ***cyp1a1*** | 0.25 | 0.37 | DN | 0.19 | 0.15 | DN | 0.31 | 0.17 | DN | 0.1 |
| C139R132 | ***dnmt1*** | 0.75 | 0.25 | DN | 0.53 | 0.52 | DN | 0.50 | 0.53 | DN | 0.0 |
| C079R030 | ***egln2*** | 0.47 | 0.46 | DN | 0.39 | 0.37 | DN | 0.47 | 0.38 | DN | 0.1 |
| C152R082 | ***epx*** | 3.12 | 3.09 | UP | 3.09 | 2.28 | UP | 3.11 | 2.69 | UP | 0.4 |
| C035R008 | ***gck*** | 0.36 | 0.57 | DN | 0.48 | 0.71 | DN | 0.47 | 0.60 | DN | -0.1 |
| C202R025 | ***gstt1*** | 0.61 | 0.60 | DN | 0.59 | 0.53 | DN | 0.60 | 0.56 | DN | 0.0 |
| C044R099 | ***hcn1*** | 5.09 | 7.00 | UP | 5.40 | 5.68 | UP | 6.05 | 5.54 | UP | 0.5 |
| C120R146 | ***hif1α*** | 0.57 | 0.45 | DN | 0.60 | 0.57 | DN | 0.51 | 0.58 | DN | -0.1 |
| C007R083 | ***hsp70*** | 3.94 | 2.59 | UP | 2.04 | 1.98 | UP | 3.26 | 2.01 | UP | 1.3 |
| C182R027 | ***hsp90aa1*** | 2.54 | 3.99 | UP | 3.08 | 3.68 | UP | 3.27 | 3.38 | UP | -0.1 |
| C021R073 | ***hsp90ab1*** | 1.56 | 2.10 | UP | 1.29 | 1.39 | UP | 1.83 | 1.34 | UP | 0.5 |
| C126R097 | ***hspd1*** | 0.45 | 0.46 | DN | 0.29 | 0.38 | DN | 0.45 | 0.34 | DN | 0.1 |
| C206R122 | ***igfbp2b1*** | 11.13 | 2.55 | UP | 0.61 | 0.60 | DN | 6.84 | 0.61 | DIFF | 6.2 |
| C183R028 | ***il8*** | 3.48 | 10.35 | UP | 1.38 | 2.63 | UP | 6.92 | 2.00 | UP | 4.9 |
| C064R147 | ***irf2*** | 2.32 | 2.52 | UP | 0.80 | 0.81 | DN | 2.42 | 0.80 | DIFF | 1.6 |
| C127R086 | ***jak2*** | 2.27 | 3.03 | UP | 1.73 | 1.47 | UP | 2.65 | 1.60 | UP | 1.0 |
| C015R051 | ***jund*** | 4.04 | 4.19 | UP | 3.18 | 3.03 | UP | 4.12 | 3.11 | UP | 1.0 |
| C098R076 | ***mhcii*** | 1.63 | 2.51 | UP | 1.04 | 1.46 | UP | 2.07 | 1.25 | UP | 0.8 |
| C081R049 | ***mmp9*** | 2.57 | 2.63 | UP | 2.01 | 1.43 | UP | 2.60 | 1.72 | UP | 0.9 |
| C020R060 | ***nckap1l*** | 2.64 | 3.84 | UP | 0.60 | 0.55 | DN | 3.24 | 0.58 | DIFF | 2.7 |
| C013R092 | ***ndufa1*** | 3.92 | 1.50 | UP | 1.02 | 1.02 | UP | 2.71 | 1.02 | UP | 1.7 |
| C097R143 | ***ndufa4*** | 3.92 | 1.50 | UP | 2.16 | 1.26 | UP | 2.71 | 1.71 | UP | 1.0 |
| C107R097 | ***pdk3*** | 1.96 | 1.95 | UP | 1.80 | 1.34 | UP | 1.95 | 1.57 | UP | 0.4 |
| C212R138 | ***prdx6*** | 0.26 | 0.18 | DN | 0.38 | 0.38 | DN | 0.22 | 0.38 | DN | -0.2 |
| C251R053 | ***rraga*** | 0.51 | 0.58 | DN | 0.53 | 0.48 | DN | 0.54 | 0.50 | DN | 0.0 |
| C236R132 | ***serpinh1*** | 10.45 | 16.18 | UP | 5.51 | 6.32 | UP | 13.32 | 5.91 | UP | 7.4 |
| C027R139 | ***tapbp*** | 1.62 | 1.97 | UP | 0.75 | 0.93 | DN | 1.80 | 0.84 | DIFF | 1.0 |
| C056R147 | ***tnfrsf6b*** | 1.71 | 2.17 | UP | 1.65 | 2.83 | UP | 1.94 | 2.24 | UP | -0.3 |
| C182R133 | ***txn*** | 2.19 | 2.15 | UP | 1.88 | 1.48 | UP | 2.17 | 1.68 | UP | 0.5 |
| C013R092 | ***ucp2*** | 0.43 | 0.36 | DN | 0.18 | 0.17 | DN | 0.40 | 0.18 | DN | 0.2 |

^1^ Refers to the unique Agilent 44K salmonid oligonucleotide microarray probe identification number.

^2^ Fold change (FC) of microarray probe or gene expression (qPCR) in Warm & Normoxic (WN) group as

compared to Control (CT) group.

^3^ Fold change (FC) of microarray probe or gene expression (qPCR) in Warm & Hypoxic (WH) group as

compared to Control (CT) group.

^4^ The direction refers to either up-regulated (UP) or down-regulated (DN) microarray probe or qPCR gene

expression for the expression (qPCR) for the respective WN or WH groups in comparison to

the CT group. The abbreviation ‘DIFF’ indicates a difference in the direction of expression change when

comparing the mean FC values obtained by the microarray or qPCR approach.

**^5^** The mean FC were calculated considering either the microarray or qPCR expression values of the WN

and WH treatment groups.

**^6^** Delta refers to the difference of mean FC between the microarray and qPCR approach per target gene.
