# Supplementary material for: The transcriptomic responses of Atlantic salmon (Salmo salar) to high temperature stress alone, and in combination with moderate hypoxia
Source: BMC Genomics. 2021 Apr 12;22:261. doi: 10.1186/s12864-021-07464-x (PMC8042886; doi:10.1186/s12864-021-07464-x)
Supplement: Supplementary file 9 — Additional file 9. Bootstrap ratios from the first two components (CP-1 and CP-2) of the component maps based on 41 target genes and seven phenotypic traits for the Warm & Normoxic (WN) and Warm & Hypoxic (WH) treatment groups. Bootstrap ratios were performed to identify the variables that contributed significantly to the variance of a given component. Bold values indicate bootstrap ratios whose magnitude exceed +/− 2 and are considered as significant. [file 12864_2021_7464_MOESM9_ESM.docx]

**Additional file 9:** **Bootstrap ratios from the first two components (CP-1 and CP-2) of the component maps based on 41 target genes and seven phenotypic traits for the Warm & Normoxic (WN) and Warm & Hypoxic (WH) treatment groups.**

|  | **Warm & Normoxic** | | **Warm &**  **Hypoxic** | |
| --- | --- | --- | --- | --- |
| **Variables** | **CP-1^1^** | **CP-2^1^** | **CP-1^1^** | **CP-2^1^** |
| **Length** | 1.0 | 0.7 | **2.0** | -0.1 |
| **Weight** | 1.4 | 1.1 | **3.1** | -0.4 |
| **SGR** | 0.3 | **2.6** | **2.7** | -0.3 |
| **CF** | 0.6 | 1.6 | **3.1** | -0.9 |
| **HSI** | 1.5 | -0.8 | 0.9 | -0.7 |
| **SSI** | 1.8 | -1.0 | -1.1 | 1.0 |
| **RVM** | 1.1 | 1.2 | -1.5 | 1.1 |
| **apod** | -1.4 | 1.1 | **-2.5** | 0.7 |
| **c1ql2** | **-6.3** | -0.5 | **-2.7** | -1.1 |
| **c3** | **2.7** | **2.8** | **2.3** | **3.0** |
| **calm** | **6.9** | 0.9 | **7.4** | 1.6 |
| **camp-a** | 0.2 | -0.8 | **-2.0** | -0.4 |
| **casp8** | **-5.2** | 0.8 | **-4.6** | 1.4 |
| **cat** | 0.4 | **2.5** | 0.5 | **2.3** |
| **cirbp** | **8.5** | 0.3 | **7.9** | 0.4 |
| **cldn3** | **2.8** | 0.9 | 0.2 | **3.0** |
| **ctsh** | **8.3** | 0.3 | **8.0** | 1.1 |
| **cul3** | **-2.0** | 1.1 | 0.3 | -0.2 |
| **cyp1a1** | **5.1** | 0.8 | **5.2** | 1.1 |
| **dnmt1** | **7.8** | -0.2 | **5.9** | 0.2 |
| **egln2** | **8.9** | -1.0 | **7.0** | -0.5 |
| **epx** | **-2.3** | 1.9 | **-2.3** | **2.0** |
| **gck** | 1.2 | 0.8 | 0.6 | 1.5 |
| **gstt1** | **4.3** | 0.6 | **4.6** | 1.2 |
| **hcn1** | **-3.6** | 0.9 | **-4** | 0.5 |
| **hif1α** | **4.3** | 1.8 | **4.1** | 1.6 |
| **hsp70** | **-2.6** | **2.6** | **-3.0** | **2.2** |
| **hsp90aa1** | **-5.3** | 1.9 | **-3.2** | 2.0 |
| **hsp90ab1** | -0.9 | **3.0** | -1.7 | **2.8** |
| **hspd1** | **8.5** | -0.3 | **7.0** | 0.1 |
| **igfbp2b1** | **2.3** | **2.5** | **2.0** | **2.7** |
| **il8** | -1.0 | -0.2 | **-2.1** | 1.2 |
| **irf2** | 1.4 | **2.9** | 1.0 | **3.1** |
| **jak2** | **-3.1** | **2.3** | **-2.9** | **2.5** |
| **jund** | **-3.6** | 0.4 | **-4.5** | 0.5 |
| **mhcii** | -0.2 | 1.4 | -1.6 | 1.8 |
| **mmp9** | -1.7 | 1.6 | -1.7 | **2.7** |
| **nckap1l** | **3.2** | 1.4 | **3.9** | 1.4 |
| **ndufa1** | 0.0 | **3.6** | 0.1 | 1.7 |
| **ndufa4** | **-2.3** | 0.1 | -0.6 | -0.2 |
| **pdk3** | **-4.3** | 1.4 | **-2.1** | -0.1 |
| **prdx6** | **11.6** | -0.4 | **10.3** | 0.2 |
| **rraga** | **7.2** | 1.1 | **8.6** | 1.2 |
| **serpinh1** | **-5.0** | **2.2** | **-6.8** | 1.5 |
| **tapbp** | 1.3 | **2.7** | 0.4 | **2.9** |
| **tnfrsf6b** | -1.3 | -0.1 | **-2.3** | 0.5 |
| **txn** | **-2.1** | -0.2 | -1.4 | 1.4 |
| **ucp2** | **5.7** | 1.0 | **6.1** | 1.3 |

^1^ Bootstrap ratios were obtained by battery of inference permutation tests to identify the response variables

that contribute significantly to the variance of the two main components (CP-1 and CP-2). Bold values

indicate bootstrap ratios whose magnitude exceed +/-2 and are considered as significant.
