# Supplementary material for: The transcriptomic responses of Atlantic salmon (Salmo salar) to high temperature stress alone, and in combination with moderate hypoxia
Source: BMC Genomics. 2021 Apr 12;22:261. doi: 10.1186/s12864-021-07464-x (PMC8042886; doi:10.1186/s12864-021-07464-x)
Supplement: Supplementary file 10 — Additional file 10. Primers used for qPCR validation analyses. Listed are gene symbol, gene name, the sequence of forward and reverse primers, the amplicon size in base pairs, GenBank accession number used for primer design and the determined primer efficiencies. See Additional file 11 for further details about BLASTn hits and primer properties. [file 12864_2021_7464_MOESM10_ESM.docx]

**Additional file 10:** **Primers used for qPCR validation analyses.**

| **Gene**  **Symbol** | **Gene**  **Name** | **Forward Primer**  **(Sequence 5'-3')** | **Reverse Primer**  **(Sequence 5'-3')** | **Size^5^**  **(bp)** | **GenBank^6^**  **Accession^#^** | **Eff ^7^**  **(%)** |
| --- | --- | --- | --- | --- | --- | --- |
| **apod** | Apolipoprotein D-like | GGAGAAACTGGAGGAGCTGTAC | ACTGAATCATGTCCCTTAGGCC | 130 | XM_014189629.1 | 97.5 |
| **c1ql2** | Complement C1q-like protein | AACCTGAGCACCTTCAATGGAA | GCGTTGCCATAATTCCCATCAA | 137 | XM_014172088.1 | 91.0 |
| **c3** | Complement C3-like | TTATTGAAGGAGTGGGCAAAGC | TCTGCTTTCACCATCTCACTCC | 142 | XM_014131265.1 | 91.5 |
| **calm (cam)** | Calmodulin | GAGATGATCAGAGAAGCCGACA | GCCAGGAGAAGGGTAGGAGA | 104 | BT058660.1 | 92.5 |
| **camp-a^1^** | Cathelicidin - paralog a | AAGCCAGAAAATGCTCCAGA | ACCCTCAGGACGACCAATTA | 107 | GQ870278.1 | 84.0 |
| **casp8** | Caspase 8 | TCCTGTCTATATAAGTGGGCGTTC | CTTTCCCGAGTGAGCTAACAGT | 80 | BT072408.1 | 89.5 |
| **cat^1^** | Catalase | CGACGATAACGTCACACAGG | GCCTGGACCCCGTTTCCATA | 177 | NM_001140302.1 | 86.0 |
| **cirbp** | Cold-inducible RNA-binding protein | TTGAGTACACAGCGGTGAATT | ACCAATCTGATGCTATGACGAGA | 132 | BT059171.1 | 98.8 |
| **cldn3** | Claudin 3 | GCTCACGTTTTTCCCACAGT | CAGTTTGGTGTTGTTCCTCTTCA | 189 | XM_014214348.1 | 89.5 |
| **ctsh** | Cathepsin H precursor | AAATGGGTATGGTGGATGCAGT | ACTGTGTCTGTGGTGTTGTGAC | 130 | BT047094.1 | 84.0 |
| **cul3** | Cullin 3 | TCAGGCCCACTACTCTTCTTACT | GCATTCACATCTTTCCCAAGGG | 127 | XM_014192069.1 | 89.5 |
| **cyp1a1^1^** | Cytochrome P450 1A1 | AGGTGGGAATGACTCGTACTC | GATGTATCCTTGACTGTGCAGT | 136 | BT045666.1 | 94.0 |
| **dnmt1** | DNA (cytosine-5)-methyltransferase 1 | TGTGTCTTAGAGCGGATCAAGG | TAGTCCGACACCTCCATCTTCT | 83 | XM_014203933.1 | 93.5 |
| **egln2** | Egl nine homolog 2 | TAGTTAAATGTGTGTGTGCGCA | TCTTACCCAGTCTATCTCACACAC | 90 | NM_001165346.1 | 89.5 |
| **epx^1^** | Eosinophil peroxidase-like | CCGTGACCCCTTCAGAATC | TCTGCTGTTTGGCCTCTGTA | 116 | BT072012.1 | 108.0 |
| **gck^2^** | Glucokinase | CTTTGGAGCCAACGGAGA | GCACCAGCTCTCCCATGTA | 131 | XM_014171080.1 | 87.0 |
| **gstt1** | Glutathione S-transferase theta 1 | CAAAGACAAGATGGATGGAGCC | CATGATCTCAACAATGGCCACC | 139 | BT046590.1 | 87.0 |
| **hcn1** | Hyperpolarization-activated cyclic nucleotide-gated channel 1 | TGCACAACAGGACAAGGAAGAT | ACCAGTCTACACAGTACCAAGTC | 105 | XM_014164789.1 | 91.0 |
| **hif1α^1^** | Hypoxia inducible factor 1 alpha | CCCATGTTCACAACAACAGC | AATGAGAAGGGGCTGAACCT | 100 | NM_001140022.1 | 88.0 |
| **hsp70^1^** | Heat shock protein HSP 70 | AGTGATCAACGACTCGACACG | CACTGCATTGGTTATAGTCTTG | 151 | BT045715.1 | 90.0 |
| **hsp90aa1** | Heat shock protein HSP 90-alpha | CGAGGACATGAAGAAGAGGCAT | ACACTGTCACCTTCTCCACTTT | 104 | KC150878.1 | 92.5 |
| **hsp90ab1** | Heat shock protein HSP 90-beta | AGGATTCCAAGGACAAGAAGAAGA | GTCAGGCTCTTGTAGAACTCCC | 145 | KC150883.1 | 93.5 |
| **hspd1** | 60 kDa heat shock protein | CGGTCCAAGCCACAAGTTAATG | CAACACTTGAATGCACGGTAGT | 133 | BT071923.1 | 87.5 |
| **igfbp2b1** | Insulin-like growth factor-binding protein 2 - paralog b1 | CTGCTTATGTTACTTGGTCTGCC | TACAGTTGATGGTGAGCTCAGG | 112 | NM_001123648.1 | 87.5 |
| **il8^3^** | Interleukin-8 | GAAAGCAGACGAATTGGTAGAC | GCTGTTGCTCAGAGTTGCAAT | 99 | BT046706 | 90.7 |
| **irf2** | Interferon regulatory factor 2 | TTCATCAGAGCAGTCACAGTCC | GACTGACTGCTCCTCATTCTCC | 107 | NM_001252351.1 | 107.5 |
| **jak2** | Tyrosine-protein kinase JAK2-like | ACCGCTCAGATATGTAAGGGTATG | AGCTCACTCTCTACCAGGATGT | 95 | XM_014171549.1 | 92.0 |
| **jund** | Transcription factor Jun-D-like | CTGGATTAGGGTAGAATAGGTGCA | ACATTGAGGGATGCAGTTCAGT | 134 | XM_014190066.1 | 86.5 |
| **mhcii** | MHC class ii antigen alpha chain | GGGCACTGGACTAGAGAGATCT | TGGCGTACATTGTTGACTGTGA | 110 | XM_014137044.1 | 94.0 |
| **mmp9^1^** | Matrix metalloproteinase 9 | GGACCTTGTGACCAGGAAAA | TGGAGGACAGACCCAGTTTC | 110 | NM_001140457.1 | 90.5 |
| **nckap1l** | Nck-associated protein 1-like | TCTGCGTCTATGTGTGACCTTC | GGTCATTGAGGTTGCACTGTTC | 95 | XM_014134027.1 | 100.0 |
| **ndufa1** | NADH dehydrogenase 1 alpha subcomplex subunit 1 | TGATGGAGAGAGACAGACGAGT | AGGTGAGATCTGGGATTAGTGGA | 89 | BT046880.1 | 91.0 |
| **ndufa4** | Cytochrome c oxidase subunit NDUFA4 | GCCTCTTTCAACACACAACACT | TCACTTTAGGGTTGGAGAGGGT | 96 | XR_001319779.1 | 86.0 |
| **pdk3** | Pyruvate dehydrogenase [lipoamide] kinase isozyme 3 | AGTACATTATTTCCCGTGGTGTCA | CCACAGTTTCCATGGTAGCAGA | 116 | NM_001139694.1 | 88.5 |
| **prdx6** | Peroxiredoxin 6 | GCGTTCATGTTGCATTTGTTGT | CGCAATTAGAAGTAAGGCAGCA | 126 | BT047207.1 | 92.5 |
| **rraga** | Ras-related GTP binding protein A | GTTGTAGTAAACTGGCAGCCTC | TTAATGAGTGTGGCTGCAAAGG | 145 | XM_014137442.1 | 89.5 |
| **serpinh1** | Serpin H1 | GACCATTCAAAAATCAACCTCA | CATGGCTCCATCAGCATTCT | 129 | XM_014214963.1 | 92.0 |
| **tapbp** | Tapasin | ATTCCCTGCTGCAATGATCCA | CCTGCTGTGACTTGGTTTCTTC | 83 | BT045317.1 | 90.0 |
| **tnfrsf6b** | Tumor necrosis factor receptor superfamily member 6b | CAACTGTCCTAGACCTTCTCACA | TGTCCAGGATATCATGCAATCGA | 87 | XM_014136455.1 | 100.5 |
| **txn** | Thioredoxin | AGGATTCCTTCTTCATTGCCCT | TTCCGACAGCCCTTTGAAGAA | 119 | BT125521.1 | 103.5 |
| **ucp2** | Mitochondrial uncoupling protein 2 | CTGATCTCTGCCGTCACCAT | AGAAGACTGATGAGGTGAAGACA | 89 | XM_014196911.1 | 87.5 |
| **eif3d^4^** | Eukaryotic translation initiation factor 3 subunit D | CTCCTCCTCCTCGTCCTCTT | GACCCCAACAAGCAAGTGAT | 105 | GE777139 | 91.5 |
| **rpl32^4^** | 60S ribosomal protein 32 | AGGCGGTTTAAGGGTCAGAT | TCGAGCTCCTTGATGTTGTG | 119 | BT043656 | 95.5 |

^1^ Primers established within the Genomic Applications Partnership Program (GAPP ^#^6604) and quality-tested again using the reference cDNA

template of the present study (see Additional file 11).

^2^ Primers previously published in Caballero-Solares et al. [1] and quality-tested again using the reference cDNA template of the present study.

^3^ Primers previously published in Soto-Dávila et al. [2] and quality-tested again using the reference cDNA template of the present study.

^4^ Primers for normalization from previous salmon transcriptome studies [3–5] and amplification efficiencies were determined using the reference

cDNA template of the present study (see Additional file 11).

^5^ Amplicon size in base pairs (bp).

^6^ Nucleotide sequence obtained from GenBank used for primer design (see Additional file 11 for BLASTn hits).

^7^ Amplification efficiencies were calculated using a 5-point 1:3 dilution series starting with cDNA representing 10 ng of input total RNA. Presented

are mean values determined from cDNA templates of the CT and WH treatment groups (n=6, N=12 total).

**References:**

1. Caballero-Solares A, Xue X, Parrish CC, Foroutani MB, Taylor RG, Rise ML. Changes in the liver transcriptome of farmed Atlantic salmon (*Salmo salar*) fed experimental diets based on terrestrial alternatives to fish meal and fish oil. BMC Genomics. 2018;19:796.

2. Soto-Dávila M, Valderrama K, Inkpen SM, Hall JR, Rise ML, Santander J. Effects of Vitamin D2 (Ergocalciferol) and D3 (Cholecalciferol) on Atlantic Salmon (*Salmo salar*) Primary Macrophage Immune Response to *Aeromonas salmonicida* subsp. *salmonicida* Infection. Front Immunol. 2020;10:3011.

3. Xue X, Hixson SM, Hori TS, Booman M, Parrish CC, Anderson DM, et al. Atlantic salmon (*Salmo salar*) liver transcriptome response to diets containing Camelina sativa products. Comp Biochem Physiol Part D Genomics Proteomics. 2015;14:1–15.

4. Caballero-Solares A, Hall JR, Xue X, Eslamloo K, Taylor RG, Parrish CC, et al. The dietary replacement of marine ingredients by terrestrial animal and plant alternatives modulates the antiviral immune response of Atlantic salmon (*Salmo salar*). Fish Shellfish Immunol. 2017;64:24–38.

5. Eslamloo K, Xue X, Hall JR, Smith NC, Caballero-Solares A, Parrish CC, et al. Transcriptome profiling of antiviral immune and dietary fatty acid dependent responses of Atlantic salmon macrophage-like cells. BMC Genomics. 2017;18:706.
